# Supplementary material for: Prevalence and correlates of paediatric guideline non-adherence for initial empirical care in six low and middle-income settings: a hospital-based cross-sectional study
Source: BMJ Open. 2024 Mar 8;14(3):e078404. doi: 10.1136/bmjopen-2023-078404 (PMC10928745; doi:10.1136/bmjopen-2023-078404)
Supplement: Supplementary data [file bmjopen-2023-078404supp001.pdf]

BMJ Publishing Group Limited (BMJ) disclaims all liability and responsibility arising from any reliance placed on this supplemental material which has been supplied by the author(s)

Prevalence and correlates of pediatric guideline non-adherence across six low and middle-income countries

SUPPLEMENTAL TABLES

Shawon RA, *et al. BMJ Open* 2024; 14:e078404. doi: 10.1136/bmjopen-2023-078404

Prevalence and correlates of pediatric guideline non-adherence across six low and middle-income countries

Supplemental Table 1: Comparison of country/institution/WHO guidelines for diagnosis of pneumonia, diarrhea and SM sub-conditions

Variables	Bangladesh	Burkina Faso	Kenya	Malawi	Pakistan	Uganda	WHO
Guideline used in site	Institutional guideline used for pneumonia[1], Diarrhea[2] and SM[3]	WHO guideline used for pneumonia and diarrhea<br><br>National guideline used for SM[4]	National guideline used for pneumonia, diarrhea,[5] and SM[6]	Institutional guidelines used for pneumonia,[7] diarrhea[8], and SM[9]	WHO guideline used for pneumonia<br><br>Institutional guideline used for diarrhea[10]<br><br>National guideline used for SM[11]	National guidelines used for pneumonia, diarrhea, and SM[12]	Pocket Book of Hospital Care for Children[13]
**Pneumonia**							
Severe Pneumonia	Cough or difficulty breathing PLUS at least one of the following:<br><br>-central cyanosis or oxygen saturation <90%<br><br>-severe respiratory distress (e.g. grunting, very severe chest indrawing)<br><br>-signs of pneumonia with a general danger sign (inability to breastfeed or drink, lethargy or unconscious,<br><br>convulsions)	Cough or difficulty breathing PLUS at least one of the following:<br><br>-central cyanosis or oxygen saturation <90%<br><br>-severe respiratory distress (e.g., grunting, very severe chest indrawing)<br><br>-signs of pneumonia with a general danger sign (inability to breastfeed or drink, lethargy or unconscious, convulsions)	Cough or difficulty breathing<br><br>PLUS, one of the danger signs:<br><br>-oxygen saturation <90%,<br><br>-cyanosis<br><br>-inability to drink/breastfeed-AVPU =V/P/U, -grunting	Cough or difficulty breathing PLUS<br><br>-central cyanosis /oxygen saturation <90%,<br><br>-severe respiratory distress (e.g., grunting, severe indrawing, head bobbing, nasal flaring), -convulsion/ loss of consciousness/ feeding/ lethargy	Cough or difficulty breathing PLUS at least one of the following:<br><br>-central cyanosis or oxygen saturation <90%<br><br>-severe respiratory distress (e.g., grunting, very severe chest indrawing)<br><br>-signs of pneumonia with a general danger sign (inability to breastfeed or drink, lethargy or unconscious,<br><br>convulsions)	Cough, fast breathing, AND at least one of the following-<br><br>-central cyanosis or<br><br>-oxygen saturation <90%<br><br>-inability to feed, vomiting everything*<br><br>-convulsions, lethargy, decreased level of consciousness<br><br>-severe respiratory distress (severe chest indrawing, grunting, nasal flaring)<br><br>-extrapulmonary features, e.g., confusion or disorientation	Cough or difficulty breathing PLUS at least one of the following:<br><br>-central cyanosis or oxygen saturation <90%<br><br>-severe respiratory distress (e.g., grunting, very severe chest indrawing)<br><br>-signs of pneumonia with a general danger sign (inability to breastfeed or drink, lethargy or unconscious, convulsions)

Prevalence and correlates of pediatric guideline non-adherence across six low and middle-income countries

Non-severe Pneumonia	Cough or difficult breathing plus at least one of the following:<br>-fast breathing<br>-lower chest wall indrawing	Cough or difficult breathing plus at least one of the following:<br>-fast breathing<br>-lower chest wall indrawing	Lower chest wall indrawing OR fast breathing	Fast breathing	Cough or difficult breathing plus at least one of the following:<br>-fast breathing<br>-lower chest wall indrawing	Cough, and fast breathing, and mild chest indrawing	Cough or difficult breathing plus at least one of the following:<br>-fast breathing<br>-lower chest wall indrawing
**Diarrhea**							
Acute watery diarrhea with severe dehydration	Acute watery diarrhea and signs of some dehydration PLUS any one of the following:<br>-lethargic/comatose<br>-unable to drink<br>-skin pinch goes back very slowly<br>-radial pulse uncountable or absent*	Diarrhea PLUS any two signs or symptoms of severe dehydration with diarrhea:<br>-lethargic, drowsy or unconscious<br>-sunken eyes<br>-sunken fontanelle<br>-drinks poorly or not able to drink<br>-skin pinch goes back very slowly<br>(>2 seconds)	Diarrhea PLUS unable to drink or AVPU<A, plus sunken eyes, or return of skin pinch >=2 secs	Diarrhea PLUS two or more of the following signs:<br>-lethargy/ unconsciousness<br>-sunken Eyes<br>-unable to drink/drinks poorly<br>-skin pinch goes back very slowly (≥ 2 seconds)	Diarrhea PLUS any two signs or symptoms of severe dehydration<br>-lethargic, drowsy or unconscious<br>-sunken eyes<br>-sunken fontanelle<br>-drinks poorly or not able to drink<br>-skin pinch goes back very slowly<br>(>2 seconds)	Diarrhea PLUS clinical features of severe dehydration<br>-lethargic, drowsy or unconscious<br>-sunken eyes<br>-sunken fontanelle<br>-drinks poorly or not able to drink<br>-skin pinch goes back very slowly<br>(>2 seconds)	Diarrhea PLUS any two signs or symptoms of severe dehydration<br>-lethargic, drowsy or unconscious<br>-sunken eyes<br>-sunken fontanelle<br>-drinks poorly or not able to drink<br>-skin pinch goes back very slowly<br>(>2 seconds)
Acute watery diarrhea with Some dehydration	Acute watery diarrhea PLUS at least two signs including one (*) sign<br>-irritable/less active*<br>-sunken eyes<br>-dry tongue<br>-thirsty (drinks eagerly)<br>-skin pinch goes back slowly*<br>-reduced radial pulse*	Diarrhea PLUS if the child has two or more of the following signs, he or she has some dehydration:<br>-restlessness or irritability<br>-thirsty and drinks eagerly<br>-sunken eyes<br>-skin pinch goes back slowly.<br>If a child has only one of the above	Diarrhea PLUS able to drink adequately but 2 or more of: Sunken eyes, return of skin pinch 1-2 secs, restlessness/ irritability	Diarrhea PLUS two or more of the following signs:<br>-restlessness/ irritability<br>-sunken eyes<br>-drinks eagerly/thirsty<br>-skin pinch goes back slowly	Diarrhea PLUS if the child has two or more of the following signs, he or she has some dehydration:<br>-restlessness or irritability<br>-thirsty and drinks eagerly<br>-sunken eyes<br>-skin pinch goes back slowly<br>If a child has only one of the above signs and one of the signs of	Diarrhea PLUS clinical features of some dehydration:<br>-restless, irritable<br>-sunken Eyes<br>-sunken fontanelle<br>- drinks eagerly, thirsty<br>-skin pinch goes back slowly; <2 seconds	Diarrhea PLUS if the child has two or more of the following signs, he or she has some dehydration:<br>-restlessness or irritability<br>-thirsty and drinks eagerly<br>-sunken eyes<br>-skin pinch goes back slowly.<br>If a child has only one of the above signs and one of the signs of<br>severe dehydration (e.g. restlessness or irritable and drinking poorly), then the<br>child also has some dehydration

Prevalence and correlates of pediatric guideline non-adherence across six low and middle-income countries

		signs and one of the signs of<br>severe dehydration (e.g., restlessness or irritable and drinking poorly), then the<br>child also has some dehydration			severe dehydration (e.g., restlessness or irritable and drinking poorly), then the<br>child also has some dehydration		
Acute watery diarrhea with no dehydration	Acute watery diarrhea PLUS normal awareness condition, eyes, tongue, thirst, skin pinch, radial pulse	Diarrhea PLUS no sign of dehydration	Diarrhea PLUS fewer than 2 of the signs of dehydration	Diarrhea PLUS not enough signs to classify as some or severe dehydration	Diarrhea PLUS no sign of dehydration	Diarrhea PLUS clinical features of no dehydration:<br>-alert<br>-eyes not sunken<br>-fontanelle not sunken<br>-drinks normally<br>-skin pinch goes back immediately	Diarrhea PLUS no sign of dehydration
Persistent diarrhea	-	Diarrhea, with or without blood, that begins acutely and lasts for ≥ 14 days with no signs of dehydration	Diarrhea, with or without blood, that begins acutely and lasts for ≥ 14 days with no signs of dehydration	Diarrhea, with or without blood, that begins acutely and lasts for ≥ 14 days with no signs of dehydration	Diarrhea, with or without blood, that begins acutely and lasts for ≥ 14 days with no signs of dehydration	Diarrhea, with or without blood, that begins acutely and lasts for ≥ 14 days with no signs of dehydration	Diarrhea, with or without blood, that begins acutely and lasts for ≥ 14 days
Severe Persistent diarrhea	-	When there is some or severe dehydration, persistent diarrhea is classified as 'severe'	When there is some or severe dehydration, persistent diarrhea is classified as 'severe'	When there is some or severe dehydration, persistent diarrhea is classified as 'severe'	When there is some or severe dehydration, persistent diarrhea is classified as 'severe'	When there is some or severe dehydration, persistent diarrhea is classified as 'severe'	When there is some or severe dehydration, persistent diarrhea is classified as 'severe'
Dysentery	Diarrhea PLUS<br>-febrile≥ 38°C<br>-passes bloody or mucoid stools*<br>-has straining or tenesmus.	Blood mixed with the stools	Blood mixed with the stools	Frequent loose stools with visible red blood	Blood mixed with the stools	Bloody diarrhea, visible blood and mucus	Blood mixed with the stools

Prevalence and correlates of pediatric guideline non-adherence across six low and middle-income countries

**SM**							
SM	Bilateral oedema of both feet OR WFH/L <-3 z scores OR MUAC<125mm for children>=12 months<br><br>For <12 months, WLZ<-3 OR bipedal oedema	Bilateral pitting oedema OR severe wasting (WHZ< -3 z scores OR MUAC<115mm)<br><br>For <6 months, WLZ<-3 OR bipedal oedema OR visible wasting	Bilateral pitting oedema OR severe wasting (WHZ< -3 z scores OR MUAC<115mm)<br><br>For <6 months, WLZ<-3 OR bipedal oedema OR visible wasting	Bilateral pitting oedema OR severe wasting (WHZ< -3 z scores OR MUAC<115mm)<br><br>For <6 months, WLZ<-3 OR bipedal oedema	Bilateral oedema of both feet OR WFH/L <-3 z scores OR MUAC<115mm OR visible severe wasting<br><br>For <6 months, WLZ<-3 OR bipedal oedema OR visible wasting	Oedema of both feet OR WFH/L <-3 z scores OR MUAC<115mm OR visible severe wasting<br><br>For <6 months, WLZ<-3 OR bipedal oedema OR visible wasting	Bilateral oedema of both feet OR WFH/L <-3 z scores OR MUAC<115mm OR visible severe wasting<br><br>For <6 months, WLZ<-3 OR bipedal oedema OR visible wasting
Hypoglycemia in SM	Blood sugar <3 mmol/L	Blood sugar <3 mmol/L	Blood sugar <3 mmol/L	Blood sugar <3 mmol/L	Blood sugar <3 mmol/L	Blood sugar <3 mmol/L	Blood sugar <3 mmol/L
Hypothermia in SM	Axillary temperature <35c and rectal temperature <35·5c	Axillary temperature <35c and rectal temperature <35·5c	Axillary temperature <35c and rectal temperature <35·5c	Axillary temperature <35c	Axillary temperature <35c and rectal temperature <35·5c	Axillary temperature <35c and rectal temperature <35·5c	Axillary temperature <35c and rectal temperature <35·5c
Dehydration in SM	Signs of some or severe dehydration	Recent fluid loss, recent change in gaze, sunken eye, absence of superficial veins	All children with watery diarrhea or reduced urine output	Signs of some or severe dehydration	All children with watery diarrhea or reduced urine output	All children with watery diarrhea or reduced urine output	All children with watery diarrhea or reduced urine output
Severe Anemia in SM	Hb<5g/dl	Hb<4g/dl	Hb<4g/dl	Hb<4 OR Hb<6 with respiratory distress	Hb<4g/dl OR Hb 4-6g/dl with respiratory distress	Hb<4g/dl OR Hb 4-6g/dl with respiratory distress	Hb<4g/dl OR Hb 4-6g/dl with respiratory distress
Skin lesion in SM	Weeping skin lesions	Skin lesions in kwashiorkor	Dermatosis (patches of skin abnormally light or dark, shedding of skin, ulceration of skin and/or weeping<br><br>lesions)	No information	Hypo-or hyperpigmentation, Desquamation, Ulceration, Exudative lesions often with secondary infections, including Candida	Skin lesions in kwashiorkor	Hypo-or hyperpigmentation, Desquamation, Ulceration, Exudative lesions often with secondary infections, including Candida
Infection in SM	Most children with SM in in-patient care have infections and all are routinely treated	All children with SM should be treated for infection even in absence of clinical signs	Nearly all children with SM have bacterial infections. (all children should routinely receive antibiotic)	Typical signs of infections are absent. (so, antibiotics recommended in all children)	Most of the children with SM in in-patient care have infections and all are routinely treated	Assume children with SM have infection	Assume children with SM have infection
SM with measles	SM with measles	SM with measles	SM with measles	-	SM with measles	SM with measles	SM with measles

1. Dhaka Hospital. icddr,b Treatment Guidelines for Community Acquired Pneumonia. :1.

2. Nutrition & Clinical Services Division , icddr , b Treatment for Cholera and other Diarrheas.

Prevalence and correlates of pediatric guideline non-adherence across six low and middle-income countries

3. Ahmed T. Manual for Standardized Management of Severely Malnourished Children. 1–49 p.

4. Ministère de la Santé. Protocole National: Prise En Charge Integree De La Malnutrition Aiguë (Pcima), Burkina Faso. 2014;154.

5. Ministry of Health Kenya. Basic Paediatric Protocols. 2016.

6. Ministry of Health Kenya. National Guidelines for Integrated Management of Acute Malnutrition. 2009.

7. Queen Elizabeth Central Hospital. Electronic protocols for the management of common childhood illnesses in Malawi: Pneumonia. 2018.

8. Queen Elizabeth Central Hospital. Electronic protocols for the management of common childhood illnesses in Malawi: Diarrhea. 2018.

9. Voskuijl W. Queen Elizabeth Central Hospital. Electronic protocols for the management of common childhood illnesses in Malawi: Malnutrition. 2018;

10. Pakistan Pediatric Association. Diarrhea in Children. 2010. p. 1–14.

11. Ministry of Health Pakistan. National Guidelines for the management of acute malnutrition among children under five and pregnant and lactating women Ministry of Health. 2009.

12. Ministry of Health Uganda. Uganda Clinical Guidelines. 2016.

13. WHO. Pocket Book of Hospital Care for Children. World Health Organization; 2013.

* These variables were not available in the dataset. The coding was done with available variables only

Note: All of the diagnosis of conditions and sub-conditions were determined based on signs and symptoms and guideline criteria with the exception of two conditions: diarrhea, pneumonia, and one sub-condition: measles (in SM). These three diagnoses were determined based upon clinician diagnosis

6

Prevalence and correlates of pediatric guideline non-adherence across six low and middle-income countries

Supplemental Table 2: Comparison of country/institution/WHO guidelines for management of pneumonia, diarrhea and SM sub-conditions

Variables	Bangladesh	Burkina Faso	Kenya	Malawi	Pakistan	Uganda	WHO
Guideline used in site	Institutional guideline used for pneumonia[1], Diarrhea[2] and SM[3]	WHO guideline used for pneumonia and diarrhea<br>National guideline used for SM[4]	National guideline used for pneumonia, diarrhea,[5] and SM[6]	Institutional guidelines used for pneumonia,[7] diarrhea[8], and SM[9]	WHO guideline used for pneumonia<br>Institutional guideline used for diarrhea[10]<br>National guideline used for SM[11]	National guidelines used for pneumonia, diarrhea, and SM[12]	Pocket Book of Hospital Care for Children[13]
**Pneumonia**							
Severe Pneumonia	Ampicillin + Gentamicin	Ampicillin (or Benzylpenicillin) and Gentamicin<br>Give oxygen if saturation <90%<br>If fever: paracetamol<br>If wheeze: bronchodilator	Penicillin and Gentamicin<br>Give oxygen<br>If wheeze: bronchodilators	X-pen + Gentamicin<br>Give oxygen<br>If fever: paracetamol<br>If wheeze: salbutamol	Ampicillin (or Benzylpenicillin) and Gentamicin<br>Give oxygen if saturation <90%<br>If fever: paracetamol<br>If wheeze: salbutamol	Ampicillin (or Benzylpenicillin) and Gentamicin<br>Give oxygen if saturation <90%<br>If wheeze: salbutamol<br>If fever- paracetamol<br>If convulsion: diazepam or phenobarbital	Ampicillin (or Benzylpenicillin) and Gentamicin<br>Give oxygen if saturation <90%<br>If fever: paracetamol<br>If wheeze: salbutamol
Non-severe Pneumonia	Amoxicillin	Amoxicillin	Amoxicillin<br>If wheeze: bronchodilators	Amoxicillin<br>If fever: paracetamol<br>If wheeze: salbutamol	Amoxicillin	Amoxicillin<br>If fever- paracetamol<br>If wheeze: salbutamol	Amoxicillin
**Diarrhea**							
Diarrhea with severe dehydration	IV fluid I/V solution containing sodium, potassium, chloride and bicarbonate (e.g. Ringer's Lactate/ cholera saline)<br>Zinc	IV Ringers lactate or normal saline<br>Zinc (once the child starts to improve sufficiently)	IV Ringers' lactate or NG rehydration<br>Zinc<br>Antimicrobials are not indicated unless there is	IV Ringers lactate or normal saline<br>Zinc (when severe dehydration is corrected)	IV Ringers lactate or normal saline<br>Zinc (once the child starts to improve sufficiently)	IV fluid or ORS by NG<br>Zinc	IV Ringers lactate or normal saline<br>Zinc (once the child starts to improve sufficiently)

Prevalence and correlates of pediatric guideline non-adherence across six low and middle-income countries

	Antibiotic only if necessary		dysentery or proven amoebiasis or giardiasis.			Avoid unnecessary antibiotic	
Diarrhea with some dehydration	ORS	ORS<br>After reassessment, if there is no dehydration, give zinc supplement	ORS, zinc<br>Antimicrobials are not indicated unless there is<br>dysentery or proven amoebiasis or giardiasis.	ORS, zinc	ORS<br>After reassessment, if there is no dehydration, give zinc supplement	ORS, zinc	ORS<br>After reassessment, if there is no dehydration, give zinc supplement
Diarrhea with no dehydration	ORS	ORS, zinc	ORS, zinc	ORS or clean water, zinc	ORS, zinc	ORS, zinc	ORS, zinc
Persistent diarrhea with no dehydration	-	Fluid, zinc, vitamin A, minerals	ORS, zinc	ORS, zinc, vitamin A, folate, multimineral	Fluid, zinc, vitamin A, minerals	ORS, zinc, vitamin A	Fluid, zinc, vitamin A, minerals
Persistent diarrhea with some or severe dehydration	-	Fluid, zinc, vitamin A, folate, multimineral	Fluid, zinc	Fluid, zinc, vitamin A, folate, multimineral	Fluid, zinc, vitamin A, folate, multimineral	Fluid, zinc, vitamin A	Fluid, zinc, vitamin A, folate, multimineral
Dysentery	Azithromycin if Shigellosis; Metronidazole if Amoebiasis<br>Continue Ampicillin, Ciprofloxacin or Ceftriaxone if indicated for other causes	Ciprofloxacin if antibiotic sensitivity<br>is unknown. If local antimicrobial sensitivity is known, follow<br>local guidelines.<br>ceftriaxone to severely ill children	Ciprofloxacin	Azithromycin or Ciprofloxacin	Ciprofloxacin or Cefixime	Ciprofloxacin	Ciprofloxacin if antibiotic sensitivity<br>is unknown. If local antimicrobial sensitivity is known, follow<br>local guidelines.<br>ceftriaxone to severely ill children
**SM**							
Hypoglycemia in SM	Oral or IV glucose	Feed or 10% glucose/ sucrose	All children with SM are at risk and should be given a feed or 10% glucose/ sucrose immediately on admission	If hypoglycemia, give a feed and oral or 10% IV glucose immediately on admission	Assume all children with SM are hypoglycemic. Give a feed or 10% glucose/ sucrose immediately on admission	All children with SM are at risk and should be given a feed or 10% glucose/ sucrose immediately on admission	All children with SM are at risk and should be given a feed or 10% glucose/ sucrose immediately on admission

Shawon RA, *et al. BMJ Open* 2024; 14:e078404. doi: 10.1136/bmjopen-2023-078404

Prevalence and correlates of pediatric guideline non-adherence across six low and middle-income countries

Hypothermia in SM	Keep warm<br>Ensure feeding	Give a feed or 10% glucose/ sucrose, warm the child, give antibiotics	Keep warm	Warm the child	Warm the child, feed 2 hourly	Feed immediately as in hypoglycemia, warm the child, give antibiotics	Feed immediately as in hypoglycemia, warm the child, give antibiotics
Dehydration in SM	ORS/IV fluid	ReSoMal except in case of shock/ unconscious	ReSoMal except in case of shock/ unconscious	ReSoMal/ORS	ReSoMal except in case of shock/ unconscious	ReSoMal except in case of shock/ unconscious	ReSoMal except in case of shock/ unconscious
Severe Anemia in SM	Blood transfusion<br>or<br>packed blood cell*	Blood transfusion + furosemide	Blood transfusion	Blood transfusion	Blood transfusion + furosemide	Blood transfusion + furosemide	Blood transfusion + furosemide
Skin lesion in SM	Potassium permanganate solution*	No zinc	No zinc	Cloxacillin or Flucloxacillin if infected skin lesion	Zinc supplementation	Zinc supplementation + ointments	Zinc supplementation
Infection in SM *(applicable to all inpatients for prevention or management of infection)*	Broad spectrum antibiotic	Ceftriaxone OR Ceftriaxone + Gentamicin OR Ceftriaxone + Metronidazole	Penicillin OR Ampicillin PLUS Gentamicin	Benzylpenicillin PLUS Gentamicin	Penicillin OR Ampicillin PLUS Gentamicin	Benzylpenicillin OR Ampicillin PLUS Gentamicin	Penicillin OR Ampicillin PLUS Gentamicin
Micronutrient deficiency in SM (*applicable to all children with SM)*	Vit A, zinc, folate, multivitamin, potassium*, magnesium*	If clinical anemia, give folic acid	If child not on premixed food the give folic acid	No information	Give folic acid, multivitamin, zinc, copper to all children with SM	If child not on premixed food the give folic acid, multivitamin, zinc, copper	If child not on premixed food the give folic acid, multivitamin, zinc, copper
SM requiring nutritional stabilization *(applicable to all* children with SM*)*	Liquid diet such as milk suji	F-75,<br>(for children<6 months, F-75 OR diluted F-100 OR breastmilk)	F-75,<br>(for children<6 months, F-75 OR diluted F-100 OR breastmilk)	F-75, to all children	F-75,<br>(for children<6 months, F-75 OR diluted F-100 OR breastmilk)	F-75,<br>(for children<6 months, F-75 OR diluted F-100 OR breastmilk)	F-75,<br>(for children<6 months, F-75 OR diluted F-100 OR breastmilk)
SM with measles	Not mentioned	Vitamin A	Vitamin A	No information	Vitamin A	Vitamin A	Vitamin A

1. Dhaka Hospital. icddr,b Treatment Guidelines for Community Acquired Pneumonia. :1.
2. Nutrition & Clinical Services Division , icddr , b Treatment for Cholera and other Diarrheas.
3. Ahmed T. Manual for Standardized Management of Severely Malnourished Children. 1–49 p.
4. Ministère de la Santé. Protocole National: Prise En Charge Integree De La Malnutrition Aiguë (Pcima), Burkina Faso. 2014;154.
5. Ministry of Health Kenya. Basic Paediatric Protocols. 2016.

Prevalence and correlates of pediatric guideline non-adherence across six low and middle-income countries

6. Ministry of Health Kenya. National Guidelines for Integrated Management of Acute Malnutrition. 2009.
7. Queen Elizabeth Central Hospital. Electronic protocols for the management of common childhood illnesses in Malawi: Pneumonia. 2018.
8. Queen Elizabeth Central Hospital. Electronic protocols for the management of common childhood illnesses in Malawi: Diarrhea. 2018.
9. Voskuijl W. Queen Elizabeth Central Hospital. Electronic protocols for the management of common childhood illnesses in Malawi: Malnutrition. 2018;
10. Pakistan Pediatric Association. Diarrhea in Children. 2010. p. 1–14.
11. Ministry of Health Pakistan. National Guidelines for the management of acute malnutrition among children under five and pregnant and lactating women Ministry of Health. 2009.
12. Ministry of Health Uganda. Uganda Clinical Guidelines. 2016.
13. WHO. Pocket Book of Hospital Care for Children. World Health Organization; 2013.

* These variables were not available in the dataset. The coding was done with available variables only

Prevalence and correlates of pediatric guideline non-adherence across six low and middle-income countries

Supplemental Table 3: Overlap between pneumonia, diarrhea, SM and other clinical diagnoses

Overlapping diagnoses	Conditions n (%)		
	Pneumonia (N=1095)	Diarrhea (N=1014)	SM (N=1096)
Pneumonia	-	418 (32)	384 (35)
Diarrhea	418 (38)	-	524 (48)
SM	384 (35)	524 (41)	-
Bronchiolitis	77 (7)	14 (1)	21 (2)
Upper respiratory tract infection	11 (1)	59 (5)	58 (5)
Tuberculosis	18 (2)	4 (0·3)	30 (3)
Otitis media	7 (0·6)	4 (0·3)	7 (0·6)
Asthma	4 (0·4)	-	1 (0·1)
Anemia	203 (19)	179 (14)	211 (19)
Sickle cell disease	5 (0·5)	1 (0·1)	4 (0·4)
Thalassemia	1 (0·1)	1 (0·1)	1 (0·1)
Renal impairment	-	1 (0·1)	3 (0·3)
Nephrotic syndrome	-	-	2 (0·2)
Liver disease	-	-	1 (0·1)
Ileus	2 (0·2)	2 (0·2)	1 (0·1)
Congenital cardiac disease	1 (0·1)	-	1 (0·1)
Sepsis	91 (8)	138 (11)	165 (15)
Malaria	108 (10)	96 (7)	137 (13)
Soft tissue infection	2 (0·2)	2 (0·2)	4 (0·4)
Urinary tract infection	3 (0·3)	8 (0·7)	3 (0·3)
Measles	48 (4)	13 (1)	17 (2)
Osteomyelitis	-	-	1 (0·1)
Febrile illness	1 (0·1)	21 (2)	4 (0·4)
Enteric fever	10 (0·9)	6 (0·7)	28 (3)
Epilepsy	1 (0·1)	-	-
Meningitis	32 (3)	14 (1)	25 (2)
Encephalopathy	2 (0·2)	4 (0·3)	2 (0·2)
Hydrocephalous	2 (0·2)	-	2 (0·2)
Developmental delay	2 (0·2)	3 (0·2)	10 (1)
Cerebral Palsy	4 (0·4)	2 (0·2)	5 (0·5)
Note: The denominator is all cases of the syndrome in column for all proportions			

Prevalence and correlates of pediatric guideline non-adherence across six low and middle-income countries

Supplemental Table 4: Guideline adherence for conditions and sub-conditions, by country

Conditions and sub-conditions	Bangladesh n %			Burkina Faso n %			Kenya n %			Malawi n %			Pakistan n %			Uganda n %			Overall n %		
	Full	Partial	None	Full	Partial	None	Full	Partial	None	Full	Partial	None	Full	Partial	None	Full	Partial	None	Full	Partial	None
**PNEUMONIA**[1]	208<br>*75*	-	71<br>*25*	36<br>*36*	50<br>*50*	15<br>*15*	40<br>*21*	147<br>*78*	1<br>*0·5*	13<br>*18*	61<br>*82*	-	17<br>*9*	157<br>*82*	18<br>*9*	101<br>*71*	42<br>*29*	-	415<br>*42*	457<br>*47*	105<br>*11*
Non-severe	101<br>*73*	-	37<br>*27*	8<br>*35*	-	15<br>*65*	4<br>*11*	33<br>*87*	1<br>*3*	10<br>*37*	17<br>*63*	-	17<br>*50*	-	17<br>*50*	14<br>*64*	8<br>*36*	-	154<br>*54*	58<br>*21*	70<br>*24*
Severe	107<br>*76*	-	34<br>*24*	28<br>*36*	50<br>*64*	-	36<br>*24*	114<br>*76*	-	3<br>*6*	44<br>*94*	-	-	158<br>*100*	1<br>*1*	87<br>*72*	34<br>*28*	-	261<br>*38*	400<br>*57*	35<br>*5*
**DIARRHEA**[2]	259<br>*66*	132<br>*33*	4<br>*1*	36<br>*51*	9<br>*13*	26<br>*37*	2<br>*2*	49<br>*46*	56<br>*52*	16<br>*27*	24<br>*40*	20<br>*33*	6<br>*12*	18<br>*37*	25<br>*51*	4<br>*5*	23<br>*30*	49<br>*64*	323<br>*32*	255<br>*25*	435<br>*43*
AWD w/ severe dehydration	6<br>*19*	22<br>*71*	3<br>*10*	12<br>*57*	3<br>*14*	6<br>*29*	-	2<br>*100*	-	-	6<br>*60*	4<br>*40*	3<br>*27*	5<br>*46*	3<br>*27*	3<br>*16*	6<br>*32*	10<br>*53*	24<br>*26*	44<br>*47*	26<br>*28*
AWD w/ some dehydration	35<br>*70*	15<br>*30*	-	3<br>*60*	-	2<br>*40*	-	25<br>*54*	21<br>*46*	1<br>*20*	2<br>*40*	2<br>*40*	1<br>*10*	2<br>*20*	7<br>*70*	1<br>*7*	7<br>*50*	6<br>*43*	41<br>*33*	51<br>*39*	38<br>*29*
AWD w/ no dehydration	218<br>*69*	95<br>*30*	1<br>*0·3*	21<br>*47*	6<br>*13*	18<br>*40*	-	21<br>*39*	33<br>*61*	15<br>*38*	13<br>*33*	12<br>*30*	2<br>*13*	2<br>*13*	12<br>*75*	-	9<br>*26*	25<br>*74*	256<br>*51*	146<br>*29*	101<br>*20*
Persistent				-	-	-	2<br>*50*	-	2<br>*50*	-	2<br>*67*	1<br>*33*	-	4<br>*80*	1<br>*20*	-	-	6<br>*100*	2<br>*11*	6<br>*33*	10<br>*56*
Severe persistent				-	-	-	-	-	-	-	1<br>*50*	1<br>*50*	-	3<br>*75*	1<br>*25*	-	-	2<br>*100*	-	4<br>*50*	4<br>*50*
Dysentery	-	-	1<br>*100*	-	-	-	-	1<br>*100*	-	-	-	-	-	2<br>*67*	1<br>*33*	-	1<br>*100*	-	-	4<br>*67*	2<br>*33*
**SM**[3]	136<br>51	133<br>*49*	-	14<br>*8*	167<br>*92*	-	80<br>*37*	138<br>*63*	-	49<br>34	34<br>*41*	-	-	118<br>*100*	-	23<br>10	204<br>90	-	302<br>*28*	794<br>*72*	-
W/ hypoglycemia[4]	-	2<br>*100*	-	181<br>*100*	-	-	90<br>*41*	114<br>*52*	14<br>*6*	-	-	-	-	24<br>*20*	94<br>*80*	81<br>*36*	134<br>*59*	12<br>*5*	352<br>*47*	274<br>*37*	120<br>*16*
W/ hypothermia	-	-	-	-	-	-	1<br>*25*	-	3<br>*75*	1<br>*50*	-	1<br>*50*	-	-	-	-	4<br>*100*	-	2<br>*20*	4<br>40	4<br>40

Prevalence and correlates of pediatric guideline non-adherence across six low and middle-income countries

SM with dehydration	71<br>*93*	-	5<br>*7*	16<br>*94*	-	1<br>*6*	10<br>*50*	-	10<br>*50*	35<br>*81*	-	8<br>*19*	8<br>*50*	-	8<br>*50*	1<br>50	-	1<br>*50*	141<br>*81*		33<br>*19*
SM with severe anemia	-	-	-	21<br>*78*	-	6<br>*22*	3<br>*100*	-	-	-	-	-	1<br>*100*	-	-	-	-	-	25<br>*81*		6<br>*19*
SM with active measles				-	-	-	-	-	-				2<br>*25*	-	6<br>*75*	1<br>*14*	-	6<br>*86*	3<br>*20*		12<br>*80*
W/ micronutrient deficiency[5]	143<br>*53*	81<br>30	45<br>*17*	1<br>*0·9*	-	113<br>*99*	114<br>*52*	-	104<br>*48*				22<br>*19*	6<br>*5*	90<br>*76*	72<br>*32*	-	155<br>*68*	352<br>*37*	87<br>*9*	507<br>*54*
W/ infection[5]	238<br>*88*	-	31<br>*12*	41<br>*23*	48<br>*27*	92<br>*51*	161<br>*74*	36<br>*16*	21<br>*10*	62<br>*75*	-	21<br>*25*	2<br>*2*	1<br>*1*	115<br>*97*	206<br>*91*	7<br>*3*	14<br>*6*	710<br>*65*	92<br>*8*	294<br>*27*
Nutritional stabilization[5]	262<br>*97*	1<br>*0·4*	6<br>*2*	153<br>*85*	-	28<br>*16*	184<br>*84*	-	34<br>*16*	62<br>*75*	-	21<br>*25*	58<br>*49*	-	60<br>*51*	147<br>*65*	-	80<br>*35*	866<br>*79*	1<br>*0·1*	229<br>*21*
W/ skin lesion	[6]	[6]	[6]										8<br>*16*	-	42<br>*84*	-	-	109<br>*100*	8<br>*5*	-	151<br>*95*

[1] For pneumonia, comorbid SM was excluded as per Kenyan pneumonia treatment guidelines
[2] For diarrhea w/dehydration, comorbid SM was excluded, per guidelines
[3] Total SM cases in any adherence category may not be equal to the summation of all SM sub-conditions in that category since these are not mutually exclusive.
[4] Hypoglycemia treatment applies to all children with SM in Uganda, Kenya, and Pakistan as per guidelines irrespective of blood glucose level.
[5] Per guidelines, all children with SM should be treated for Infection and micronutrient deficiency and all children require nutritional stabilization. Micronutrients include Vit A, Zinc, Multivitamin, & Folic acid if therapeutic feed not given
[6] Variable for skin lesion treatment in Bangladesh not available in the data

Notes: (1) The denominator for all percentages is the total number of children with a specific condition, (2) AWD= Acute Watery Diarrhea, (3) A "-" indicates that there were no cases in the sample with the diagnosis (4) a blank cell indicates there are no relevant recommendation for a condition in the country, (5) In case of a tie, comparatively higher adherence level between the tie categories is assumed to be the majority

Shawon RA, *et al. BMJ Open* 2024; 14:e078404. doi: 10.1136/bmjopen-2023-078404

Prevalence and correlates of pediatric guideline non-adherence across six low and middle-income countries

Supplemental Table 5: Guideline adherence for specific recommendations, by sub-conditions

Recommendations	Bangladesh<br>n<br>%		Burkina Faso<br>n<br>%		Kenya<br>n<br>%		Malawi<br>n<br>%		Pakistan<br>n<br>%		Uganda<br>n<br>%		Overall<br>n<br>%	
	Full adherence	Not adherent	Full adherence	Not adherent	Full adherence	Not adherent	Full adherence	Not adherent	Full adherence	Not adherent	Full adherence	Not adherent	Full adherence	Not adherent
**PNEUMONIA[1]**														
Antibiotics for severe pneumonia	107<br>*76*	34<br>*24*	36<br>*46*	42<br>*54*	102<br>*68*	48<br>*32*	42<br>*89*	5<br>*11*	-	158<br>*100*	98<br>*81*	23<br>*19*	329<br>*52*	303<br>*48*
Antibiotics for non-severe pneumonia	101<br>*73*	37<br>*27*	8<br>*35*	15<br>*65*	5<br>*13*	33<br>*87*	12<br>*44*	15<br>*56*	17<br>*50*	17<br>*50*	16<br>*73*	6<br>*27*	129<br>*54*	112<br>*46*
Oxygen for severe pneumonia			78<br>*100 [3]*	-	71<br>*47 [4]*	79<br>*53 [4]*	6<br>*13 [4]*	41<br>*87 [4]*	158<br>*100 [3]*	-	121<br>*100 [3]*	-	434<br>*78*	120<br>*22*
Paracetamol for fever (Severe Pneumonia)			74<br>*95*	4<br>*5*			47<br>*100*	-	156<br>*99*	2<br>*1*	119<br>*98*	2<br>*2*	396<br>*98*	8<br>*2*
Paracetamol for fever (non-severe Pneumonia)							27<br>*100*	-			22<br>*100*	-	49<br>*100*	-
Bronchodilator for wheeze (severe pneumonia)			67<br>*86*	11<br>*14*	134<br>*89*	16<br>*11*	39<br>*83*	8<br>*17*	128<br>*81*	30<br>*19*	109<br>*90*	12<br>*10*	477<br>*86*	77<br>*14*
Bronchodilator for wheeze (non-severe pneumonia)					36<br>*95*	2<br>*5*	20<br>*74*	7<br>*26*			20<br>*91*	2<br>*9*	76<br>*87*	11<br>*13*
**DIARRHEA[2]**														
Rehydration for AWD with severe dehydration	9<br>*29*	22<br>*71*	14<br>*67*	7<br>*33*	2<br>*100*	-	3<br>*30*	7<br>*70*	8<br>*73*	3<br>*27*	9<br>*47*	10<br>*53*	45<br>*48*	49<br>*52*
Zinc for AWD with severe dehydration	22<br>*71*	9<br>*29*	13<br>*62*	8<br>*38*	1<br>*50*	1<br>*50*	4<br>*40*	6<br>*60*	3<br>*27*	8<br>*73*	3<br>*16*	16<br>*84*	46<br>*49*	47<br>*51*
Rehydration for AWD with some dehydration	49<br>*98*	1<br>*2*	3<br>*60*	2<br>*40*	17<br>*37*	29<br>*63*	3<br>*60*	2<br>*40*	1<br>*10*	9<br>*90*	8<br>*57*	6<br>*43*	81<br>*80*	20<br>*20*
Zinc for AWD with some dehydration			3<br>*60*	2<br>*40*	22<br>*48*	24<br>*52*	1<br>*20*	4<br>*80*	3<br>*30*	7<br>*70*	1<br>*7*	13<br>*93*	30<br>*28*	76<br>*72*

Shawon RA, *et al. BMJ Open* 2024; 14:e078404. doi: 10.1136/bmjopen-2023-078404

Prevalence and correlates of pediatric guideline non-adherence across six low and middle-income countries

Rehydration for AWD with no dehydration	300	14	23	22	15	39	25	15	4	12	8	26	375	89
	96	*4*	*51*	*49*	*28*	*72*	*63*	*38*	*25*	*75*	*24*	*77*	*81*	*19*
No antibiotic in AWD	291	104	70	1	82	20	43	12	33	4	44	23	651	154
	74	*26*	*99*	*1*	*80*	*20*	*78*	*22*	*89*	*11*	*66*	*34*	*81*	*20*
Zinc for AWD with no dehydration			26	19	14	40	21	19	2	14	3	31	66	397
			58	*42*	*26*	*74*	*53*	*47*	*12*	*88*	*9*	*91*	*14*	*86*
Rehydration for persistent diarrhea			-	-	2	2	2	1	3	2	-	6	7	9
					50	*50*	*67*	*33*	*60*	*40*		*100*	*44*	*56*
Zinc for persistent diarrhea			-	-	2	2	1	2	1	4	-	6	4	12
					50	*50*	*33*	*67*	*20*	*80*		*100*	*25*	*75*
Vitamin for persistent diarrhea[5]			-	-			-	3			-	6	-	9
								100				*100*		*100*
Folate for persistent diarrhea			-	-			-	3					-	3
								100						*100*
Microminerals for persistent diarrhea			-	-			-	3					-	3
								100						*100*
Rehydration for severe persistent diarrhea			-	-			1	1	3	1	-	2	4	4
							50	50	*75*	*25*		*100*	*50*	*50*
Zinc for severe persistent diarrhea			-	-			-	2	-	4	-	2	-	8
								100		*100*		*100*		*100*
Vitamin for severe persistent diarrhea[5]			-	-			-	2			-	2	-	4
								100				*100*		*100*
Folate for severe persistent diarrhea			-	-			-	2					-	2
								100						*100*
Microminerals for severe persistent diarrhea			-	-			-	2					-	2
								100						*100*
Rehydration for dysentery			-	-	-	1	-	-	2	1	-	1	2	4
						100			*67*	*33*		*100*	*33*	*67*
Zinc for dysentery			-	-	-	1	-	-	1	2	-	1	1	5
						100			*33*	*67*		*100*	17	*83*
Antibiotics for dysentery	-	1	-	-	1	-	-	-	-	3	1	-	2	4
		100			*100*					*100*	*100*		*33*	*67*
**SM**														
Antibiotics in SM	236	33	41	140	161	57	62	21	2	116	206	21	710	386
	88	*12*	*23*	*77*	*74*	*26*	*75*	*25*	*2*	*98*	*91*	*9*	*65*	*35*
Fluid for dehydration and SM	71	5	16	1	10	10	35	8	8	8	1	1	141	33
	93	7	*94*	*6*	*50*	*50*	*81*	*19*	*50*	*50*	*50*	*50*	*81*	*19*

Shawon RA, *et al. BMJ Open* 2024; 14:e078404. doi: 10.1136/bmjopen-2023-078404

Prevalence and correlates of pediatric guideline non-adherence across six low and middle-income countries

Blood transfusion in SM with severe anemia	-	-	21	6	3	-	-	-	1	-	-	-	25	6
			78	*22*	*100*				*100*				*81*	*19*
Vitamin A in SM with measles			-	-	-	-			2	6	1	6	3	12
									25	*75*	*14*	*86*	*20*	*80*
Micronutrients in SM	143	126	68	113	114	104			22	96	72	155	467	629
	53	*47*	*38*	*62*	*52*	*48*			*19*	*81*	*32*	*68*	*43*	*57*
Fortified food in SM	262	7	153	28	184	34	62	21	58	60	147	80	866	230
	97	*3*	*85*	*16*	*84*	*16*	*74*	*25*	*49*	*51*	*65*	*35*	*79*	*21*
Zinc for skin lesion in SM									8	42	-	109	8	151
									16	*84*		*100*	*5*	*95*

[1] For pneumonia, comorbid SM was excluded as per Kenyan pneumonia treatment guidelines
[2] For diarrhea, SM cases are excluded from all countries
[3] Oxygen is recommended for severe pneumonia cases with SpO2<90% only
[4] Oxygen is recommended for all severe pneumonia cases
[5] Vitamins include vitamin A, folate, both, and/or multivitamin
Notes: (1) The denominator for all percentages is the total number of children with a specific condition, (2) AWD= Acute Watery Diarrhea, (3) A "-" indicates that there were no cases in the sample with the diagnosis (4) a blank cell indicates there are no relevant recommendation for a condition in the country

Supplemental Table 6: Adherence to pneumonia and associated sub-conditions, excluding children with SM (sensitivity analysis)

Pneumonia sub-conditions and specific recommendations	Total N	Full adherence n (%)	Partial adherence n (%)	No adherence n (%)
**Pneumonia**	659	187 (28)	385 (58)	87 (13)
**Non-severe Pneumonia**	169	54 (32)	56 (33)	59 (34)
Antibiotics	169	57 (34)		112 (66)
Paracetamol if fever	33	33 (100)		
Bronchodilator if wheeze	71	62 (87)		9 (13)
**Severe Pneumonia**	490	133 (27)	329 (67)	28 (6)
Antibiotics	490	242 (49)		248 (51)
Oxygen	430[1]	320 (74)		110 (26)
Paracetamol if fever	280	274 (98)		6 (2)
Bronchodilator if wheeze	430	372 (87)		58 (13)

[1] Oxygen is only given when low saturation in sever pneumonia in some countries

Prevalence and correlates of pediatric guideline non-adherence across six low and middle-income countries

Supplemental Table 7: Overall guideline adherence for conditions and sub-conditions without comorbidities

Conditions and sub-conditions	Overall n %		
	Full	Partial	None
**PNEUMONIA**	51 (27)	120 (64)	17 (9)
Non-severe	8 (21)	18 (47)	12 (32)
Severe	43 (29)	102 (68)	5 (3)
**DIARRHEA**	111 (37)	148 (49)	45 (15)
AWD w/ severe dehydration	1 (5)	14 (64)	7 (32)
AWD w/ some dehydration	4 (11)	28 (74)	6 (16)
AWD w/ no dehydration	106 (47)	105 (46)	16 (7)
Persistent	-	1 (33)	2 (67)
Severe persistent	-	-	1 (100)
Dysentery	-	4 (67)	2 (33)
**SM**	22 (22)	78 (78)	
W/ hypoglycemia	2 (67)	1 (33)	-
W/ hypothermia	-	3 (100)	-
SM with dehydration	2 (33)		4 (67)
SM with severe anemia	-	-	-
SM with active measles	-	-	-
W/ micronutrient deficiency	54 (54)	-	46 (46)
W/ infection	75 (75)	9 (9)	16 (16)
SM requiring nutritional stabilization	76 (76)	-	24 (24)
W/ skin lesion	1 (3)	-	34 (97)

Notes: (1) The denominator for all percentages is the total number of children with a specific condition without comorbidities, (2) AWD= Acute Watery Diarrhea, (3) A "-" indicates that there were no cases in the sample with the diagnosis and without comorbidities

Shawon RA, *et al. BMJ Open* 2024; 14:e078404. doi: 10.1136/bmjopen-2023-078404
