# Supplementary material for: Prevalence and correlates of paediatric guideline non-adherence for initial empirical care in six low and middle-income settings: a hospital-based cross-sectional study
Source: BMJ Open. 2024 Mar 8;14(3):e078404. doi: 10.1136/bmjopen-2023-078404 (PMC10928745; doi:10.1136/bmjopen-2023-078404)
Supplement: Author's manuscript [file bmjopen-2023-078404.draft_revisions.pdf]

BMJ Open is committed to open peer review. As part of this commitment we make the peer review history of every article we publish publicly available.

When an article is published we post the peer reviewers' comments and the authors' responses online. We also post the versions of the paper that were used during peer review. These are the versions that the peer review comments apply to.

The versions of the paper that follow are the versions that were submitted during the peer review process. They are not the versions of record or the final published versions. They should not be cited or distributed as the published version of this manuscript.

BMJ Open is an open access journal and the full, final, typeset and author-corrected version of record of the manuscript is available on our site with no access controls, subscription charges or pay-per-view fees (http://bmjopen.bmj.com).

If you have any questions on BMJ Open's open peer review process please email info.bmjopen@bmj.com

BMJ Open

Prevalence and correlates of pediatric guideline non-adherence for initial empiric care in six low and middle-income settings

Journal:	*BMJ Open*
Manuscript ID	bmjopen-2023-078404
Article Type:	Original research
Date Submitted by the Author:	02-Aug-2023

Complete List of Authors:	Shawon , R.A; University of Washington,<br>Denno, Donna; University of Washington<br>Tickell, Kirkby; University of Washington, Global Health; Childhood Acute Illness and Nutrition Network,<br>Atuhairwe, Michael; Makerere University<br>Bandsma, Robert; Centre for Global Child Health<br>Mupere, Ezekiel ; Makerere University, Paediatrics and Child Health<br>Voskuijl, Wieger ; Amsterdam University Medical Centres, Pediatrics<br>Mbale, Emmie; University of Malawi, Department of Pediatrics<br>Ahmed, Tahmeed; International Centre for Diarrhoeal Disease Research Bangladesh<br>Chisti, Md.; International Centre for Diarrhoeal Disease Research Bangladesh<br>Saleem, Ali; Aga Khan University Hospital<br>Ngari, Moses; Clinical Trials Facility<br>DIALLO, Hama; Universite Joseph Ki-Zerbo<br>Berkley, James ; KEMRI/Wellcome Trust Research Programme, Clinical Research<br>Walson, Judd; University of Washington<br>Means, Arianna; University of Washington

Keywords:	PAEDIATRICS, INFECTIOUS DISEASES, PUBLIC HEALTH

SCHOLARONE™
Manuscripts

I, the Submitting Author has the right to grant and does grant on behalf of all authors of the Work (as defined in the below author licence), an exclusive licence and/or a non-exclusive licence for contributions from authors who are: i) UK Crown employees; ii) where BMJ has agreed a CC-BY licence shall apply, and/or iii) in accordance with the terms applicable for US Federal Government officers or employees acting as part of their official duties; on a worldwide, perpetual, irrevocable, royalty-free basis to BMJ Publishing Group Ltd ("BMJ") its licensees and where the relevant Journal is co-owned by BMJ to the co-owners of the Journal, to publish the Work in this journal and any other BMJ products and to exploit all rights, as set out in our licence.

The Submitting Author accepts and understands that any supply made under these terms is made by BMJ to the Submitting Author unless you are acting as an employee on behalf of your employer or a postgraduate student of an affiliated institution which is paying any applicable article publishing charge ("APC") for Open Access articles. Where the Submitting Author wishes to make the Work available on an Open Access basis (and intends to pay the relevant APC), the terms of reuse of such Open Access shall be governed by a Creative Commons licence – details of these licences and which Creative Commons licence will apply to this Work are set out in our licence referred to above.

Other than as permitted in any relevant BMJ Author's Self Archiving Policies, I confirm this Work has not been accepted for publication elsewhere, is not being considered for publication elsewhere and does not duplicate material already published. I confirm all authors consent to publication of this Work and authorise the granting of this licence.

For peer review only

TITLE

Prevalence and correlates of pediatric guideline non-adherence for initial empiric care in six low and middle-income settings

AUTHORS

Riffat Ara Shawon, MPH[1,2]
Donna M. Denno, MD[1,3,4]
Kirkby D. Tickell, PhD[1,2,4]
Michael Atuhairwe, MBChB [4,8]
Robert H J Bandsma, PhD[4,13]
Ezekiel Mupere, PhD[4,8,9]
Wieger Voskuijl, PhD[4,10,11,12]
Emmie Mbale, MBBS[4, 14]
Tahmeed Ahmed, PhD[15]
Md. Jobayer Chisti, PhD[15]
Ali Faisal Saleem, MBBS[16]
Moses Ngari, PhD[5]
Abdoulaye Hama Diallo, PhD[17]
James A. Berkley, FRCPCH[4,5,6]
Judd L. Walson, MD[1,2,3,4,7]
Arianna Rubin Means, PhD[1]

CORRESPONDING AUTHOR INFORMATION

Name of the Corresponding Author: Riffat Ara Shawon
Affiliations:
1. Department of Global Health, University of Washington, Seattle, WA, USA.
2. Department of Epidemiology, University of Washington, Seattle, WA, USA.
Email Address: riasha47@gmail.com; shawon@uw.edu

For peer review only

AUTHOR AFFILIATIONS:

1. Department of Global Health, University of Washington, Seattle, WA, USA.
2. Department of Epidemiology, University of Washington, Seattle, WA, USA.
3. Departments of Pediatrics, University of Washington, Seattle, WA, USA.
4. The Childhood Acute Illness & Nutrition (CHAIN) Network, Nairobi, Kenya.
5. KEMRI/Wellcome Trust Research Programme, Kilifi, Kenya.
6. Centre for Tropical Medicine & Global Health, Nuffield Department of Medicine, University of Oxford, Oxford, UK.
7. Department of Medicine, University of Washington, Seattle, WA, USA.
8. Uganda-Case Western Reserve University Research Collaboration, Makerere University, Kampala, Uganda.
9. Department of Paediatrics and Child Health School of Medicine College of Health Sciences, Makerere University.
10. Amsterdam Centre for Global Child Health, Emma Children's Hospital, Amsterdam University Medical Centres.
11. Amsterdam Institute for Global Health and Development, Amsterdam University Medical Centres.
12. Kamuzu University for Health Sciences (formerly College of Medicine), Blanytre, Malawi.
13. Hospital for Sick Children, Translational Medicine Program and Centre for Global Child Health, Toronto, Canada.
14. Department of Pediatrics, College of Medicine, University of Malawi, Blantyre, Malawi.
15. International Centre for Diarrhoeal Disease Research, Bangladesh (icddr,b), Mohakhali, Dhaka, Bangladesh.
16. Aga Khan University Hospital, Karachi, Sindh, Pakistan.
17. University Joseph KI-ZERBO, Ouagadougou, Burkina Faso.

For peer review only

FULL ADDRESSES OF AUTHORS

Riffat Ara Shawon
Address: Hans Rosling Center for Population Health, Box 351620
3980 15th Ave NE, Seattle, WA 98105
email: shawon@uw.edu

Donna M. Denno
Address: Hans Rosling Center for Population Health, Box 351620
3980 15th Ave NE, Seattle, WA 98105
email: ddenno@uw.edu

Kirkby D. Tickell
Address: Hans Rosling Center for Population Health, Box 351620
3980 15th Ave NE, Seattle, WA 98105
email: kirkbt@uw.edu

Michael Atuhairwe, MBChB
Address: Uganda-Case Western Reserve University Research Collaboration, Makerere University, Upper Mulago Hill Road. Box 7072 Kampala, Uganda
email: michaelatu@gmail.com

Robert H J Bandsma, PhD
Address: Hospital for Sick Children, 555 University Avenue, Toronto, Ontario, Canada
email: robert.bandsma@sickkids.ca

Ezekiel Mupere, PhD
Address: Uganda-Case Western Reserve University Research Collaboration, Makerere University, Upper Mulago Hill Road. Box 7072 Kampala, Uganda.
Email: mupez@yahoo.com

Wieger P. Voskuijl, PhD
Address: Meibergdreef 9, 1105 AZ Amsterdam, Netherlands

For peer review only

email: w.p.voskuijl@amsterdamumc.nl

Emmie Mbale, MBBS
Address: University of Malawi, Chirunga Road, Zomba, Malawi
email: emmiembale@gmail.com

Tahmeed Ahmed. PhD
Address: Dhaka Hospital, Nutrition and Clinical Services Division, icddr,b, Dhaka, Bangladesh
email: tahmeed@icddrb.org

Md. Jobayer Chisti, PhD
Address: Dhaka Hospital, Nutrition and Clinical Services Division, icddr,b, Dhaka, Bangladesh
email: chisti@icddrb.org

Ali Faisal Saleem, MBBS
Address: Aga Khan University Hospital, National Stadium Road, Bahadurabad, Karachi, Sindh, Pakistan.
email: ali.saleem@aku.edu

Moses Ngari
Address: KEMRI/Wellcome Trust Research Programme, Clinical Trials Facility, Hospital road, Kilifi, 80108, Kenya
email: mngari@kemri-wellcome.org

Abdoulaye Hama Diallo
Address: Avenue Charles De Gaulle, Ouagadougou, Burkina Faso
email: hamadial@yahoo.fr

James A. Berkley
Address: Oxford OX1 2JD, United Kingdom
email: jberkley@kemri-wellcome.org

For peer review only

Judd L. Walson
Address: Hans Rosling Center for Population Health, Box 351620
3980 15th Ave NE, Seattle, WA 98105
email: walson@uw.edu

Arianna Rubin Means, PhD
Address: Hans Rosling Center for Population Health, Box 351620
3980 15th Ave NE, Seattle, WA 98105
email: aerubin@uw.edu

For peer review only

ABSTRACT

Objectives: This study evaluated the prevalence and correlates of guideline non-adherence for common childhood illnesses in low-resource settings.

Design and setting: We used secondary cross-sectional data from eight healthcare facilities in six Asian and African countries.

Participants: A total of 2,796 children aged 2-23 months hospitalized between November 2016, and January 2019 with pneumonia, diarrhea, or severe malnutrition and without HIV infection were included in this study.

Primary outcome measures: We identified children treated with full, partial, or non-adherent initial inpatient care according to site-specific standard-of-care guidelines for pneumonia, diarrhea, and severe malnutrition (SM) within the first 24-hours of admission. Correlates of guideline non-adherence were identified using generalized estimating equations.

Results: Fully adherent care was delivered to 32% of children admitted with diarrhea, 34% of children with pneumonia, and 28% of children with SM when a strict definition of adherence was applied. Non-adherence to recommendations was most common for oxygen and antibiotics for pneumonia; fluid, zinc, and antibiotics for diarrhea; and vitamin A and zinc for SM. Non-adherence varied by site. Pneumonia guideline non-adherence was more likely among patients with severe disease (OR:1·82; 95% CI:1·38, 2·34) compared to non-severe disease. Diarrhea guideline non-adherence was more likely among lower asset quintile groups (OR:1·16; 95% CI:1·01, 1·35), older children (OR:1·10; 95% CI:1·06, 1·13) and children presenting with wasting (OR:6·44; 95% CI:4·33, 9·57) compared to those with higher assets, younger age, and not wasted.

For peer review only

157 *Conclusions*: Non-adherence to pediatric guidelines was common and associated with older age,
158 disease severity, and comorbidities, and lower household economic status. These findings
159 highlight opportunities to improve guidelines by adding clarity to specific recommendations.

For peer review only

STRENGTHS AND LIMITATIONS OF THIS STUDY

- To our knowledge, this is the largest multi-country study conducted to date evaluating the prevalence of guideline non-adherence for three major causes of pediatric mortality and their sub-conditions: pneumonia, diarrhea, and severe malnutrition.
- Strict interpretation of guidelines with the support of a clinical advisory group revealed contradictions, complexities and gaps within guidelines, including challenges in applying guidelines when children have multiple comorbidities.
- This study uniquely investigated patient-level (ex. socioeconomic status), provider-level (ex. clinician perceived risk at admission that the child may die during hospitalization), and facility-level correlates (ex. patient arrival during weekdays vs weekends/holidays) of guideline non-adherence.
- Non-adherence in this study was measured in hospitals with highly trained and monitored staff which may not be generalizable in all settings.
- Observed non-adherence could be driven by purposeful deviations from guidelines, resulting from resource limitations, environmental factors (e.g., local antibiotic resistance pattern) and vague or unclear guidelines. It was not possible to differentiate purposeful and accidental guideline non-adherence.

For peer review only

INTRODUCTION

Globally, an estimated 14,000 under-five children die every day.[1] Leading causes of mortality in the post-neonatal period include pneumonia (13%) and diarrhea (8%), and undernutrition is an underlying cause in nearly half of child deaths under the age of 5 years.[2,3] Low-resource settings bear a disproportionate burden of child mortality, with over 50% of these deaths occurring in sub-Saharan Africa and one-third in South Asia.[4]

World Health Organization (WHO) normative guidelines offer informed decision support for managing undernutrition, diarrhea, pneumonia and other conditions. These guidelines are often adapted to national contexts for adoption at the country-level. When applied with high fidelity, clinical guidelines can improve health outcomes of targeted populations and reduce case fatality.[5] Yet, guidelines for reducing mortality in children under five, such as the Integrated Management of Childhood Illness and other related WHO guidelines, are inconsistently applied.[6] Guideline non-adherent case management can be driven by inadequate assessment, treatment, and monitoring, local clinical culture, limitations in infrastructure, supply chain challenges, political context, differences in patient-practitioner goals, disease trajectory, patient comorbidities and clarity and applicability of recommendations. Thus, in some circumstances guideline non-adherence may be clinically justified. [7–11]

Evidence from multiple settings indicates that training alone does not affect adherence to guidelines.[12] However, there is minimal information regarding the patient, health service provider and institution specific factors that influence fidelity to clinical guidelines in low-and-middle-income countries (LMICs).[13] We aim to identify the frequency of guideline non-adherence and factors influencing non-adherence during inpatient pediatric treatment for pneumonia, diarrhea,

For peer review only

and severe malnutrition (SM) across eight facilities in six LMICs. This information will be helpful in identifying specific opportunities to support healthcare workers in their daily patient care.

METHODS

Study setting

This analysis utilized de-identified data from the Childhood Acute Illness and Nutrition (CHAIN) study, a multi-site prospective cohort study that identifies risk factors for mortality in hospitalized acutely ill children and after discharge.[14] The CHAIN study took place between November 2016, and January 2019. We include children enrolled in CHAIN aged 2-23 months, hospitalized with pneumonia, diarrhea, or SM (referred to within the guidelines as severe acute malnutrition or SAM) and without HIV infection. Children with malnutrition were deliberately oversampled in CHAIN.

Urban CHAIN sites included hospitals in Karachi (Pakistan), Dhaka (Bangladesh), Kampala (Uganda), Blantyre (Malawi), and Nairobi (Kenya), and rural sites were in Banfora (Burkina Faso), Migori (Kenya), and Matlab (Bangladesh). The sites included public, private non-profit, referral, and district hospitals and often receive patients with complex or severe disease compared to lower-level facilities. Further information about hospital characteristics can be found elsewhere.[15] One CHAIN site, Kilifi County Hospital, Kenya, was omitted from the analysis, as there was a potential conflict between this analysis and ongoing internal guideline adherence auditing processes. Participating Bangladesh facilities were diarrhea specialty centers, also admitting children with other conditions such as pneumonia and SM. In the CHAIN study, sites received a pre-study resource audit, and ongoing performance audit and feedback. Sites were also supported by trained

For peer review only

research clinicians to ensure high standards of clinical care.[16] Thus, in this secondary analysis, we assume clinical staff were highly trained and aware of ongoing monitoring.

Key informants from each site identified institutional or national guidelines available for each condition, and if none, WHO guidelines were used for this analysis (Supplemental Table 1). All sites within the same country reported using the same guidelines. Some facilities might draw from multiple guidelines to manage a child, but for the purposes of this analysis a single and most primary guideline was used as the standard-of-care to abstract relevant diagnostic criteria and treatment recommendations for each condition within each site. To identify children with pneumonia, diarrhea, SM, and sub-conditions we used CHAIN records of clinician diagnoses or, if necessary, we applied guideline diagnostic criteria (Supplemental Table 1).

Conditions and sub-conditions

We evaluated guideline adherence for initial treatment of pneumonia, diarrhea, and SM, the most common conditions across all sites. We also assessed adherence to sub-conditions, including non-severe and severe pneumonia, acute watery diarrhea (AWD) with severe, some and no dehydration, non-severe and severe persistent diarrhea, dysentery, and SM complicated by hypoglycemia, hypothermia, dehydration, severe anemia, measles, micronutrient deficiency, infection, and skin lesions.

We reviewed diagnoses and initial management provided within the first 24-hours of admission. However, we reviewed and counted antibiotic management up to 48-hours to allow for emergence of new clinical information.

For peer review only

Defining adherence to guidelines

We formed a clinical advisory group comprising three CHAIN clinicians, two pediatricians and one general practitioner, simulating a real-world scenario of clinical decision-making with peer support. The group met four times and discussed complex clinical scenarios (e.g., a child with both non-severe pneumonia and SM) to resolve ambiguity guideline interpretation when needed and to allow flexibility in situations of multi-morbidities with conflicting antibiotic recommendations. For example, if a child had both non-severe pneumonia and SM, treatment with broad-spectrum injectable antibiotics (e.g., ampicillin and gentamicin) recommended for SM was considered guideline adherent for non-severe pneumonia, even though pneumonia guidelines recommended an oral antibiotic (e.g., amoxicillin).

Diarrhea, pneumonia, and SM guideline adherence was calculated as the proportion of children initially treated with adherent care for all relevant sub-conditions within 24-hours of admission. We reported the site-specific number and proportion of children managed with full, partial, or non-adherent care. Fully adherent care included providing all treatment components in guidelines (e.g., recommended antibiotics, oral and IV fluid, therapeutic feeds). Non-adherent care indicated that a child was not provided any recommended treatments. Partial adherence indicated that some, but not all, guideline-indicated treatments were provided. We also reported adherence to specific recommendations (e.g., oxygen supplementation for severe pneumonia). The analysis was agnostic to the relative importance of different recommendations.

Statistical analysis

For peer review only

Descriptive statistics were applied to report the proportion of adherence to treatment for conditions (full, partial, and non-adherence) and sub-conditions (adherence and non-adherence) within each site and overall.

The dichotomous outcome for the correlates analysis was any non-adherence to guidelines (including partial non-adherence). We explored patient-specific correlates including age, sex, socio-economic status (composite variable standardized using asset quintiles, derived from the Demographic and Health Survey wealth index), pre-admission care-seeking history , and wasting (weight-for-height Z score <-2).[17] Other patient-specific correlates included comorbidities and presence of WHO and condition-specific danger signs (i.e., obstructed breathing, respiratory distress, shock, cyanosis, convulsions, severe anemia, severe dehydration, profuse watery diarrhea, severe vomiting, impaired consciousness) as assessed by study clinicians.[18] Provider-specific correlates included clinician perceived risk at admission that the child may die during hospitalization. Facility-specific correlates included patient arrival during weekdays vs weekends/holidays according to each country's holiday and weekend conventions.

We determined the association between the pre-specified correlates of interest and guideline non-adherence for each condition by utilizing generalized estimating equations (GEE) with a logit link, with clustering at the hospital-level, robust standard error estimates and an exchangeable correlation structure. This multivariable analysis was evaluated at a 5% alpha level.

Two independent variables had some missingness: care-seeking history (~2% missingness) and clinician risk impression upon admission (~0·1% missingness). We used multiple imputation by chained equation for these variables under the assumption of data missing at random using all non-missing exposure variables in the imputation model. Rubin's rule was used to combine logistic regression outputs of ten iterations.[19] In sensitivity analysis, we repeated all analyses with complete

For peer review only

cases. Because the results did not substantially differ between the two approaches, we presented results from the imputed data.

Kenya's pneumonia guideline excluded children with SM from its treatment recommendations because of conflicting antibiotic recommendations in the national guidelines. Because antibiotic recommendations often conflict between SM and pneumonia recommendations, especially for non-severe pneumonia, we conducted a sensitivity analysis in which we excluded children with SM from pneumonia-specific analyses for all countries. Furthermore, all diarrhea guidelines indicated that diarrhea with comorbid SM requires special management, especially if the child has dehydration. Per the guidelines, SM cases were excluded when calculating diarrhea guideline adherence for all diarrhea sub-conditions. Adherence to rehydration recommendations for children with SM, diarrhea and dehydration was assessed under the SM with dehydration sub-condition.

Because SM assessment in children >6 months does not account for low birth weight or prematurity, we calculated SM guideline adherence amongst children <6 months versus ≥6 months in additional sensitivity analysis. Finally, we conducted a sensitivity analysis for conditions and sub-conditions without any comorbidities overall across all sites. Stata v.16 was used for all analyses.

Role of the funding source

The funding sources had no role in study design, in the collection, analysis, and interpretation of data, in the writing of or decision to submit the report for publication.

Patient and public involvement

Patients or the public were not involved in the design, conduct, reporting, or dissemination plans of our research.

For peer review only

RESULTS

Sample characteristics

Diagnosis and treatment criteria were drawn from thirteen different guidelines (Supplemental Tables 1 & 2) and applied to 2,796 pediatric hospitalizations. A quarter of patient admissions were in Bangladesh (n=708). More than half were male (57%) and 42% were aged 12-23 months (Table 1).

Table 1: Demographic characteristics and prevalence of pneumonia, diarrhea and SM

	Bangladesh (n=708)		Burkina Faso (n=429)		Kenya (n=533)		Malawi (n=311)		Pakistan (n=349)		Uganda (n=466)		Total (N=2,796)	
	n	%[1]	n	%[1]	n	%[1]	n	%[1]	n	%[1]	n	%[1]	n	%[1]
Demographics of children														
Age at admission (months)														
2-5	164	23	66	15	122	23	54	17	106	30	49	11	561	20
6-11	313	44	127	30	191	36	104	33	122	35	201	43	1058	37
12-23	231	33	236	55	220	41	153	49	121	35	216	46	1177	42
Male sex	426	60	245	57	297	56	172	55	190	54	258	55	1588	57
Assets Quintiles														
1st (lowest)	58	8	141	33	155	29	92	30	1	0·3	17	4	464	17
2nd	106	15	183	43	71	13	91	29	4	0·7	83	18	538	19
3rd	140	20	72	17	113	21	59	19	30	9	168	36	582	21
4th	187	26	19	4	111	21	34	11	107	31	138	30	596	21
5th (highest)	217	31	14	3	83	16	35	11	207	59	60	13	616	22
Conditions and sub-conditions														
Pneumonia	279	39	101	24	306	57	74	24	192	55	143	31	1095	39
Non-severe	138	19	23	5	52	10	27	9	34	10	22	5	296	10
Severe	141	20	78	18	254	48	47	15	158	45	121	26	799	29
Diarrhea[2]	576	81	71	16	103	20	60	20	49	14	76	16	935	32
AWD w/ severe dehydration	31	4	21	5	2	0·4	10	3	11	3	19	4	94	3
AWD w/ some dehydration	50	7	5	1	46	9	5	2	10	3	14	3	130	5

For peer review only

AWD w/ no dehydration	494	70	45	10	54	10	40	13	16	5	34	7	683	23
Persistent[3]			-	-	-	-	3	1	5	1	6	1	14	0·5
Severe persistent[3]			-	-	-	-	2	1	4	1	2	0·4	8	0·3
Dysentery	1	0·1	-	-	1	0.1	-	-	3	0·9	1	0·2	6	0·2
SM[4]	269	38	181	42	218	41	83	27	118	34	227	49	1096	39
w/ hypoglycemia	2	0·3	5	1	6	1	-	-	3	0·9	7	2	23	2
w/ hypothermia	-	-	-	-	4	0·8	2	0·6	-	-	4	0·9	10	0·9
w/ dehydration[5]	76	11	17	4	20	4	43	14	16	5	2	0·4	174	16
w/ measles	2	0·3	-	-	-	-	-	-	8	2	7	2	17	2
w/ severe anemia	-	-	27	6	3	0·6	-	-	1	0·3	-	-	31	3
w/ skin lesions	57	8	35	8	72	14	15	5	50	14	109	23	337	31
Conditions with Comorbidities[6]														
Pneumonia w/ other comorbidities	253	36	96	22	228	43	54	17	157	45	116	25	905	32
Diarrhea w/ other comorbidities	437	62	70	16	78	15	30	10	41	12	46	10	702	25
SM w/ other comorbidities	267	38	168	39	206	39	68	22	115	33	172	37	996	36

[1] The denominator for all percentages is the total number of children treated in each site
[2] Diarrhea with dehydration in children with SM is included under SM
[3] Persistent diarrhea involves no signs of dehydration and severe persistent diarrhea involves signs of dehydration
[4] SM sub conditions are not mutually exclusive. Also, guidelines assume that all SM cases have infection, micronutrient deficiency, and electrolyte imbalance and should be treated accordingly. The other sub-conditions listed in the table for SAM may or may not be present and are not mutually exclusive
[5] Excludes SM with shock/unconsciousness based on guideline recommendations
[6] Comorbidities: bronchiolitis, lower respiratory tract infection, upper respiratory tract infections, tuberculosis, otitis media, asthma, anemia, sickle cell disease, thalassemia, nephrotic syndrome, nephritis, other renal diseases, liver disease, ileus, cardiac disease, sepsis, malaria, soft tissue disease, urinary tract infection, measles, varicella, osteomyelitis, febrile illness, enteric fever, epilepsy, meningitis, encephalopathy, hydrocephalus, developmental delay, cerebral palsy, gastroenteritis, SM (excluding the condition originally being assessed as applicable)
Notes: (1) AWD= Acute Watery Diarrhea, (2) Columns percentages may not exactly be 100% due to rounding, (3) Decimals are rounded to the nearest integer except when the proportion is less than 1%, (4) No exclusions are made in this table for prevalence of conditions and sub-conditions unless noted otherwise, (5) A "-" indicates that there were no cases in the sample with the diagnosis (6) A blank cell indicates there are no specific recommendations for the given condition in the country

Prevalence of conditions and sub-conditions

Approximately one-third of children were admitted with severe pneumonia and 10% with non-severe pneumonia (Table 1). Over half in Kenya (57%, n=533) and Pakistan (55%, n=349) were

For peer review only

admitted with pneumonia. About 32% of children across facilities were admitted with diarrhea (excluding children with concurrent SM). The Bangladesh facilities, being diarrhea specialty hospitals, had the highest proportion of children admitted with diarrhea (81%, n=576) while Pakistan had the lowest (14%, n=349). Across facilities, 39% of children had SM, which is expected given that CHAIN purposefully overenrolled children with wasting. Approximately one-third of children were admitted with at least two of the three conditions: diarrhea, pneumonia and SM. Supplemental Table 3 provides more detail about comorbidities in the sample.

Prevalence of guideline adherence

The guidelines used by countries have significant differences (Supplemental table 1). Prevalence of guideline adherence varied by country and guideline recommendations. Overall, 42% of children with pneumonia were treated with fully adherent care (Table 2 and Supplemental Table 4). The majority of the non-severe pneumonia (54%) cases were treated with full adherence, whereas only 38% of children with severe pneumonia were treated with full adherence. Lower adherence to pneumonia guidelines was driven primarily by non-provision of oxygen when recommended and prescription of different antibiotics, typically broader spectrum, from those recommended (e.g., ceftriaxone rather than ampicillin and gentamicin) (Table 3 and Supplemental Table 5). In sensitivity analyses in which comorbid SM was excluded, we continued to observe partial adherence in most pneumonia cases (58%) (Supplemental Table 6).

Table 2: Summary of guideline adherence, by conditions and sub-conditions

Subtitle: Care is categorized as mostly guideline adherent (light blue), partially guideline adherent (blue) or not adherent (dark blue).

Conditions and sub-conditions	Bangladesh	Burkina Faso	Kenya	Malawi	Pakistan	Uganda	Overall
PNEUMONIA[1]							
Pneumonia (combined)							

For peer review only

Non-severe							
Severe							
DIARRHEA[2]							
Diarrhea (combined)							
AWD w/ severe dehydration							
AWD w/ some dehydration							
AWD w/ no dehydration							
Persistent diarrhea		-					
Severe persistent diarrhea		-	-				
Dysentery		-		-			
SM							
SM (combined)							
W/ hypoglycemia[3]				-			
W/ hypothermia	-	-			-		
W/ dehydration							
W/ severe anemia	-			-			
W/ measles		-	-				
W/ micronutrient deficiency[4] (all SM)							
W/ infection[4] (all SM)							
Requiring nutritional stabilization[4] (all SM)							
W/ skin lesion	-						

Cell colors indicate that care was mostly (e.g., the highest proportion) fully guideline adherent (light blue), partially guideline adherent (blue) or not adherent (dark blue). Please see Supplemental Table 4 for quantitative details on these adherence levels

[1] For pneumonia, comorbid SM was excluded as per Kenyan pneumonia treatment guidelines

[2] For diarrhea, comorbid SM was excluded, per guidelines

[3] Hypoglycemia treatment applies to all children with SM irrespective of serum glucose level in Uganda, Kenya, Pakistan and Bangladesh, per guidelines

[4] Per guidelines, all children with SM should be treated for presumptive infection, micronutrient deficiency and require nutritional stabilization. Micronutrients include Vit A, zinc, folate, potassium, magnesium

Notes: (1) AWD= Acute Watery Diarrhea (2) A "-" indicates that there were no children with the diagnosis (3) a blank cell indicates there are no specific recommendations for a condition in the country, (4) Supplemental Table 4 provides detailed conditions and sub-conditions specific estimates of adherence for all cases

For peer review only - http://bmjopen.bmj.com/site/about/guidelines.xhtml

Across all sites, only 32% children with diarrhea were treated with fully guideline adherent care (Supplemental Table 4). Bangladesh and Burkina Faso had the highest levels of full adherence while Kenya, Pakistan and Uganda had the lowest (Table 2). The recommendation commonly exhibiting least adherence across diarrhea sub-conditions was provision of zinc (Table 3). Persistent diarrhea and dysentery were typically treated with partially adherent or non-adherent care across sites. Overall, 81% of children were treated will full adherence to the recommendation of withholding antibiotics for acute watery diarrhea without comorbidity. About 41% children with diarrhea were admitted with comorbidities, primarily SM, for which antibiotics were indicated.

Overall, most children with SM were treated with partially adherent care (72%) (Supplemental Table 4). Full adherence was observed in a minority of patients with hypothermia (20%), measles (20%), or skin lesions (5%). In contrast, most patients with SM and hypoglycemia (47%), dehydration (81%), severe anemia (81%), and infection (65%), were treated with fully adherent care. Nutritional stabilization was also started in most children with SM (79%). In sensitivity analysis, we found that full adherence to overall SM guidelines was higher in children 6 months and older (29%) compared to less than 6 months (19%). Recommendations were less detailed for <6 months and simply stated that management should be similar to >6 months old, except some feeding recommendations. Full adherence was lower in <6 months children compared to 6 months and older for recommendations to treat hypoglycemia (43 % vs 54%), dehydration (79% vs 82%), presumed micronutrient deficiency (35% vs 44%), and presumed infection (61% vs 65%). Fully adherent care was higher in <6 months versus in 6 months and older for treatment of skin lesion (15% vs 4%) and severe anemia (100% vs 79%). Adherence to nutritional stabilization recommendations did not differ between the age groups (79% fully adherent).

For peer review only

We conducted sensitivity analyses to describe overall guideline adherence patterns for pneumonia, diarrhea and SM without comorbidities (Supplemental Table 7) and found no appreciable difference except lower adherence for non-severe pneumonia and higher adherence for micronutrients in SM with dehydration.

Table 3: Number and proportion of children treated with fully guideline adherent care, by specific guideline recommendations

Subtitle: Each cell indicates the number and proportion of children receiving fully adherent care. Cells colored in dark blue indicate <65% full adherence, blue indicate 65-80% full adherence and light blue indicate >80% full adherence

Specific recommendations	Bangladesh	Burkina Faso	Kenya	Malawi	Pakistan	Uganda	Overall
	n (%)						
PNEUMONIA[1]							
Antibiotics for severe pneumonia	107 (76)	36 (46)	102 (68)	42 (89)	0 (0)	98 (81)	329(52)
Antibiotics for non-severe pneumonia	101 (73)	8 (35)	5 (13)	12 (44)	17 (50)	16 (73)	129(54)
Oxygen for severe pneumonia		78 (100)[3]	71 (47)[4]	6 (13)[4]	158 (100)[3]	121 (100)[3]	434(78)
Paracetamol for fever (Severe Pneumonia)		74 (95)		47 (100)	156 (99)	119 (98)	396(98)
Paracetamol for fever (non-severe Pneumonia)				27 (100)		22 (100)	49 (100)
Bronchodilator for wheeze (severe pneumonia)		67 (86)	134 (89)	39 (83)	128 (81)	109 (90)	477(86)
Bronchodilator for wheeze (non-severe pneumonia)			36 (95)	20 (74)		20 (91)	76 (87)
DIARRHEA[2]							
Rehydration for AWD with severe dehydration	9 (29)	14 (67)	2 (100)	3 (30)	8 (73)	9 (47)	45 (52)
Zinc for AWD with severe dehydration	22 (71)	13 (62)	1 (50)	4 (40)	3 (27)	3 (16)	46 (49)
Rehydration for AWD with some dehydration	49 (98)	3 (60)	17 (37)	3 (60)	1 (10)	8 (57)	81(80)
Zinc for AWD with some dehydration		3 (60)	22 (48)	1 (20)	3 (30)	1 (7)	30 (28)
Rehydration for AWD with no dehydration	300 (96)	23 (51)	15 (28)	25 (63)	4 (25)	8 (24)	375 (81)
No antibiotic in AWD without comorbidity	291 (74)	70 (99)	82 (80)	43 (78)	33 (89)	44 (66)	651 (81)

For peer review only - http://bmjopen.bmj.com/site/about/guidelines.xhtml

Zinc for AWD with no dehydration		26 (58)	14 (26)	21 (53)	2 (12)	3 (9)	66 (14)
Rehydration for persistent diarrhea		-	2 (50)	2 (67)	3 (60)	0 (0)	7 (44)
Zinc for persistent diarrhea		-	2 (50)	1 (33)	1 (20)	0 (0)	4 (25)
Multi-vitamins for persistent diarrhea[5]		-		0 (0)		0 (0)	0 (0)
Microminerals[6] for persistent diarrhea		-		0 (0)			0 (0)
Rehydration for severe persistent diarrhea		-		1 (50)	3 (75)	0 (0)	4 (50)
Zinc for severe persistent diarrhea		-		0 (0)	0 (0)	0 (0)	3 (30)
Multi-vitamins for severe persistent diarrhea[5]		-		0 (0)		0 (0)	0 (0)
Microminerals[6] for severe persistent diarrhea		-		0 (0)			0 (100)
Rehydration for dysentery		-	0 (0)	-	2 (67)	0 (0)	2 (33)
Zinc for dysentery		-	0 (0)	-	1 (33)	0 (0)	1 (17)
Antibiotics for dysentery	0 (0)	-	1 (100)	-	0 (0)	1 (100)	2 (33)
SM							
Specific antibiotics in SM	236 (88)	41 (23)	161 (74)	62 (75)	2 (2)	206 (91)	710 (65)
Fluid in SM with dehydration	71 (93)	16 (94)	10 (50)	35 (81)	8 (50)	1 (50)	141 (33)
Blood transfusion in SM with severe anemia	-	21 (78)	3 (100)	-	1 (100)	-	25 (81)
Vitamin A in SM with measles		-	-		2 (25)	1 (14)	3 (20)
Micronutrients in SM	143 (53)	68 (38)	114 (52)		22 (19)	72 (32)	471(43)
Therapeutic food in SM	262 (97)	153 (85)	184 (84)	62 (74)	58 (49)	147 (65)	866 (79)
Zinc for skin lesion in SM					8 (16)	0 (0)	8 (5)

Each cell indicates the number and % of fully adherent care. Cells colored in dark blue indicate <65% full adherence, blue indicate 65-80% full adherence and light blue indicate >80% full adherence

[1] For pneumonia, comorbid SM was excluded as per Kenyan pneumonia treatment guidelines

[2] For diarrhea, comorbid SM was excluded, per guidelines

[3] Oxygen is recommended for severe pneumonia with SpO2<90% only

[4] Oxygen is recommended to all children with severe pneumonia

[5] Vitamins include vitamin A, folate, both, and/or multivitamin

[6] Microminerals was an available variable in the CHAIN data without details of what the minerals are

Notes: (1) The denominator for all percentages is the total number of children with a specific condition, (2) AWD= Acute Watery Diarrhea, (3) A "-" indicates there were no cases in the sample with the diagnosis. (4) A blank cell indicates there are no relevant recommendation for a condition in the country, (5) Supplemental Table 5 provides detailed recommendation-specific estimates of adherence levels for all cases

Correlates of guideline non-adherence

For peer review only

In multivariable analysis, children with severe pneumonia were more likely to be treated with guideline non-adherent care (OR:1·81; 95% CI:1·36, 2·41) (Table 4) compared to non-severe pneumonia. For diarrhea, children who were older (OR:1·10; 95% CI:1·06, 1·43) and wasted at admission (OR:6·69; 95% CI:4·53, 10·17) were more likely to be treated with guideline non-adherent care compared to younger or non-wasted children. Children from higher asset quintiles (OR:0·85; 95% CI:0·73, 0·99) were less likely to receive non-adherent care. For SM, none of the factors appeared to be statistically significant in this analysis.

For peer review only

Table 4: Correlates of pneumonia, diarrhea and SM guideline non-adherence

Variables	Univariable analysis		
	OR	90% CI	p-value
**PNEUMONIA**			
Older age	0·98	0·96, 1·00	0·113
Male sex	0·85	0·66, 1·09	0·199
Wasted at admission	0·95	0·71, 1·26	0·706
Losing weight/not gaining weight problem detected at admission	0·80	0·58, 1·10	0·166
Presence of danger sign	1·12	0.81, 1·55	0·500
Severe Pneumonia[1]	1·81	1·36, 2·41	<0·001
Previous hospitalization	1·08	0·94, 1·26	0·28
Child likely to die[2]	1·15	0·82, 1·63	0·42
Asset quintile[3]	0·99	0·87, 1·11	0·77
Seeking prior treatment[4]			
Did not seek treatment	1·26	0·79, 2·02	0·332
Sought treatment from shop/pharmacy	1·02	0·76, 1·35	0·911
Sought treatment from traditional healer/herbalist/homeopathist	1·55	0·84, 2·88	0·164
Presence of comorbidity	1·08	0·76, 1·53	0·676
Referred to hospital by health worker	1·23	0·92, 1·66	0·169
Admitted on a holiday	0·87	0·57, 1·34	0·568
**DIARRHEA**			
Older age	1·10	1·06, 1·43	<0·001
Male sex	1·27	0·91, 1·77	0·155
Wasted at admission	6·79	4·53, 10·17	<0·001
Losing weight/not gaining weight problem detected at admission[a]	1·11	0·72, 1·71	0·649
Presence of danger sign	1·50	0·90, 2·50	0·117
Previous hospitalization	1·01	0·81, 1·25	0·933
Child likely to die[2]	0·85	0·54, 1·35	0·496

Asset quintile[3]	0·85	0·73, 0·99	0·033
Seeking prior treatment[4]			
Did not seek treatment	1·03	0·57, 1·89	0·900
Sought treatment from shop/pharmacy	0·91	0·62, 1·34	0·632
Sought treatment from traditional healer/herbalist/homeopathist	0·44	0·19, 1·06	0·066
Presence of comorbidity	0·87	0·61, 1·29	0·527
Referred to hospital by health worker	1·09	0·71, 1·67	0·709
Admitted on a holiday	0·86	0·55, 1·35	0·525
Diarrhea sub-condition[5]			
AWD w/ severe dehydration	1·70	0·96, 3·01	0·067
AWD w/ some dehydration	0·63	0·38, 1·06	0·085
**SM**			
Older age	0·98	0·94, 1·01	0·200
Male sex	1·41	0·93, 2·15	0·106
Losing weight/not gaining weight problem detected at admission	0·59	0·35, 1·01	0·056
Danger sign	0·64	0·35, 1·19	0·161
Previous hospitalization	1·05	0·82, 1·33	0·700
Child likely to die[2]	0·74	0·44, 1·26	0·270
Assets quintile[3]	1·07	0·88, 1·31	0·495
Presence of comorbidity	1·34	0·69, 2·62	0·390
Seeking prior treatment[4]			
Did not seek treatment	2·31	0·77, 7·00	0·137
Sought treatment from shop/pharmacy	1·39	0·80, 2·40	0·243
Sought treatment from traditional healer/herbalist/homeopathist	0·94	0·45, 1·99	0·875
Referred to hospital by health worker	0·84	0·53, 1·35	0·476
Admitted on a holiday	1·04	0·47, 2·31	0·919
SM w/ hypoglycemia	0·54	0·18, 1·59	0·263
SM w/ hypothermia	0·72	0·10, 5·16	0·747
SM w/ dehydration	2·49	1·01, 6·13	0·048
SM w/ skin lesion	1·46	0·89, 2·38	0·132

Reference categories: [1] non-severe pneumonia, [2]child unlikely to die, [3] lowest quintile, [4] sought treatment from a medical facility, [5] AWD w/ no dehydration

[a]Losing weight/not gaining weight problem detected at admission (detected by history taking) and admission MUAC group (detected by anthropometry measurement) were highly correlated. Admission MUAC group was used in the multivariable analysis as this is an objective measure

Notes: (1) AWD=Acute watery diarrhea; OR=Odds Ratio (2) There were not enough cases to run regressions with SM w/ measles and SM w/ severe anemia

389

390 DISCUSSION

We examined reported pediatric treatment data from eight health facilities across a range of geographic and ecologic settings in Africa and South Asia to examine adherence to standard-of-care treatment guidelines. Guidelines vary considerably across countries in level of detail and scope of included recommendations. Overall, partial and non-adherence to pneumonia, diarrhea, and SM guidelines was common, although variable by country and across individual recommendations.

Pneumonia guideline adherence

Most children with pneumonia were treated with partial adherence. Lowest adherence was noted for antibiotic recommendations and oxygen supplementation guidance. While amoxicillin is recommended for non-severe pneumonia by all guidelines, broader spectrum antibiotics were typically prescribed in most cases. Hospitalization is not indicated for non-severe pneumonia alone, thus it was likely that children with non-severe pneumonia had other comorbidities or were perceived by a clinician to be at risk of deterioration. A study in Sudan found only 18% of hospitalized children with pneumonia received recommended antibiotic, and that broader spectrum antimicrobials were commonly prescribed.[20] A systematic review of qualitative studies revealed that previous clinical practice, peer practices, fear of patient complications, diagnostic uncertainty, patient comorbidities, desiring a rapid cure, time pressure, and cost saving influenced antibiotic prescription decisions.[21] Clinical guidelines did not emerge as a key driver of initial empiric antibiotic use in the review.[21]

Kenyan and Malawian guidelines include oxygen supplementation for all children with severe pneumonia; adherence to this recommendation was uncommon. In contrast, supplementation is recommended based on oxygen saturation levels in the other four countries' guidelines, and full adherence was observed in most children. When faced with vague or broad guidelines, healthcare

For peer review only

workers may be required to lean on clinical judgement for decision-making on allocating limited resources such as oxygen.[22] More specific recommendations such as allocation to those at highest risk of clinical deterioration or based upon oxygen saturation or specific clinical signs could help users prioritize when therapeutics are in short supply and drive higher guideline adherence.[23]

Severe pneumonia treatment recommendations contained more components and were less likely to be fully adhered to compared to non-severe pneumonia. Resource shortages, including oxygen and pulse oximetry, challenge the ability to adhere to recommendations.[24]

Diarrhea guideline adherence

Most children with diarrhea were treated with guideline non-adherent care. Fluid management had the lowest adherence, including non-provision of recommended ORS and IVF. Vitamin A and folate for persistent diarrhea and zinc for any form of diarrhea were also subject to low adherence. Other studies in low-resource settings also reported low adherence to fluid and zinc reccomendations,[25] This may also be driven by clinical practice culture, stock outs, lack of human resources, and low provider knowledge about zinc treatment for diarrhea.[26]

All guidelines considered in this analysis recommend that clinicians should not provide antibiotics for diarrhea in the absence of dysentery, cholera, or an indicated comorbidity. This recommendation was fully adhered to in most cases. However, for patients with dysentery, providers frequently prescribed a different antibiotic than that specified in guidelines. In contrast, a prior study in two Kenyan facilities reported frequent over-prescription of initial antibiotics in non-dysentery uncomplicated diarrhea and under-prescription in dysentery.[27] Our analysis documented and accounted for the presence of comorbidities indicative of initial antibiotic treatment, which is a novel addition to the literature. This might explain some of the over

For peer review only

prescription of antibiotics in uncomplicated diarrhea observed in the literature, which typically do not take comorbidities into account.

In this study, children with diarrhea who presented with wasting at were less likely to receive guideline adherent care, compared to non-wasted children. This could result from over-cautiousness of providers in dehydration management among wasted children because of the potentially narrow safety range of fluids in severe wasting.[28] Additionally, children with diarrhea from poorer households were less likely to receive guideline adherent care. Further investigation is necessary to determine if out-of-pocket costs present a barrier to appropriate care. Within our population of children under 2 years old, older children were also less likely to receive adherent care compared to younger children. Evidence from other diseases (malaria and lower respiratory tract infections), similarly, found older children received less guideline adherent care.[29] Some guideline recommendations are different for younger vs older children, especially less than 6 months vs 6 months or older. For example, breast milk replaces ORS in cases requiring oral rehydration. The latter requires a hospital supply or purchase ability by the family which could lead to lower adherence in older children.

SM guideline adherence

Most patients with SM were treated with partially adherent care in this study. A Kenyan study investigating inpatient SM guideline adherence previously reported low adherence for hypoglycemia (15%), hypothermia (5%), and dehydration (31%) management.[30]

Strengths and limitations

This analysis was based on a strict interpretation of guidelines. If antibiotics for pneumonia or dysentery were prescribed based on local sensitivity patterns not described in guidelines, this was

For peer review only

458 considered antibiotic non-adherence. This is true particularly in Pakistan where study physicians
459 report that they purposefully avoid first line antibiotics due to high pathogen resistance prevalence.

460 It is possible that treatment was received without documentation as a prescription. For example,
461 zinc or ORS could have been provided directly to caregivers without a prescription and therefore
462 not recorded in the study database. Furthermore, it is possible that delayed documentation could
463 have resulted in non-adherence overestimation. These issues will be explored in future qualitative
464 research. Additionally, data from this study was collected in the context of a cohort study in
465 hospitals with highly trained and monitored staff and with a patient population with a
466 disproportionately high proportion of wasted children; the frequency of guideline non-adherence
467 observed might not be generalizable to a routine clinical care setting as a result.

468 Importantly, adherence in this study is not a proxy for quality of care. Non-adherence to guidelines
469 may not always mean that care was sub-optimal, rather decisions to deviate from guideline are
470 often intentional and valid, driven by complex factors such as institutional norms, resource
471 shortages, or clinical judgement. Measuring quality of care solely based upon adherence to
472 guidelines may mask important nuances not currently captured by guidelines, or unclarity in the
473 guidelines themselves.

474

475 CONCLUSION

476 Pediatric treatment guidelines aim to consolidate evidence-based recommendations into
477 straightforward clinical tools, relevant across cadres and settings. This study examined guideline
478 adherence for pneumonia, diarrhea, and SM and relevant sub-conditions. We found instances of
479 both unexplained and explained guideline non-adherence. These findings highlight opportunities

For peer review only

to improve guidelines by increasing specificity for individual recommendations. For example, clarifying oxygen supplementation recommendations for severe pneumonia, or SM management in children under 6 months of age may help improve adherence and clinical outcomes. However, this study also highlights opportunity for strategic nuance, such as providing core guideline recommendations requiring high fidelity with required resource support in addition to ensuring that recommendations are tailored at the local level (e.g., use of antibiotics based on local sensitivity patterns, end-user perspectives, resources and cost considerations of treatment). This analysis also identified areas of complexity and potential confusion in guideline interpretation especially when a child has multiple conditions, underscoring the importance of addressing comorbidities in guidelines. To improve pediatric guideline adherence in low-resource settings, critical review of existing guidelines is necessary to ensure that they fully support clinicians in complex clinical scenarios.

CONTRIBUTORS

ARM, JLW, DMD, and KBT conceptualized the study. ARM, DMD, KBT, and MA contributed to the methodology. ARM and KBT were responsible for data curation. ARM and RAS accessed and verified the data underlying the study. RAS did the formal analysis and prepared the original draft of the manuscript. ARM, JLW, JAB, DMD, KBT, MA, RHB, EMu, WV, EMb, MJC, TA, AFS, MN, and AHD contributed to critically reviewing and editing the manuscript. All authors had full access to all the data in the study and accept responsibility to submit for publication.

DATA SHARING

For peer review only

Any reasonable requests to share data will be considered by the CHAIN consortium. Data requests should be sent to the corresponding author.

DECLARATION OF INTERESTS

We declare no competing interests.

For peer review only

FUNDING

This secondary analysis of the CHAIN data was funded by the National Institutes of Health (NIH), grant number 5R03HD099270-02. The primary CHAIN study was supported by the Bill & Melinda Gates Foundation, grant number OPP1131320. For the purpose of Open Access, the author has applied a CC-BY public copyright license to any author accepted manuscript version arising from this submission.

ACKNOWLEDGEMENT

We thank the CHAIN Network for providing the data for this secondary analysis, and all CHAIN participants, hospitals, and the CHAIN clinical and data management teams. We are grateful to the CHAIN sites who responded to our requests for study site information. This work was supported, in whole or in part, by the Bill & Melinda Gates Foundation [OPP131320]. Under the grant conditions of the Foundation, a Creative Commons Attribution 4.0 Generic License has already been assigned to the Author Accepted Manuscript version that might arise from this submission.

ETHICAL APPROVAL

The parent CHAIN cohort study protocol was approved by the Oxford Tropical Research Ethics Committee, and the institutional review boards of all partner sites. This secondary analysis was approved by the University of Washington Human Subjects Division (IRB ID #00006878).

For peer review only

REFERENCE

1. UNICEF. Under-five mortality. [Internet]. 2019 [cited 2021 Dec 12]. Available from: https://data.unicef.org/topic/child-survival/under-five-mortality/

2. Black RE, Victora CG, Walker SP, Bhutta ZA, Christian P, de Onis M, et al. Maternal and child undernutrition and overweight in low-income and middle-income countries. Lancet (London, England). 2013 Aug;382(9890):427–51.

3. Hug L, Sharrow D, You D. Levels & Trends in Child Mortality [Internet]. 2017. Available from: https://reliefweb.int/sites/reliefweb.int/files/resources/Levels-and-trends-in-child-mortality-IGME-English_2021.pdf

4. Kuruvilla S, Bustreo F, Kuo T, Mishra CK, Taylor K, Fogstad H, et al. The Global strategy for women's, children's and adolescents' health (2016-2030): a roadmap based on evidence and country experience. Bull World Health Organ [Internet]. 2016/05/02. 2016 May 1;94(5):398–400. Available from: https://pubmed.ncbi.nlm.nih.gov/27147772

5. Hossain M, Chisti MJ, Hossain MI, Mahfuz M, Islam MM, Ahmed T. Efficacy of World Health Organization guideline in facility-based reduction of mortality in severely malnourished children from low and middle income countries: A systematic review and meta-analysis. J Paediatr Child Health. 2017 May;53(5):474–9.

6. Turner EL, Nielsen KR, Jamal SM, von Saint André-von Arnim A, Musa NL. A Review of Pediatric Critical Care in Resource-Limited Settings: A Look at Past, Present, and Future Directions. Front Pediatr. 2016;4:5.

7. Nolan T, Angos P, Cunha AJ, Muhe L, Qazi S, Simoes EA, et al. Quality of hospital care for seriously ill children in less-developed countries. Lancet (London, England). 2001

For peer review only

Jan;357(9250):106–10.

8. English M, Ntoburi S, Wagai J, Mbindyo P, Opiyo N, Ayieko P, et al. An intervention to improve paediatric and newborn care in Kenyan district hospitals: Understanding the context. Implement Sci. 2009;4(1):1–8.

9. Solà I, Carrasco JM, Díaz Del Campo P, Gracia J, Orrego C, Martínez F, et al. Attitudes and perceptions about clinical guidelines: A qualitative study with spanish physicians. PLoS One. 2014;9(2).

10. Arts DL, Voncken AG, Medlock S, Abu-Hanna A, van Weert HCPM. Reasons for intentional guideline non-adherence: A systematic review. Int J Med Inform. 2016 May;89:55–62.

11. Hendaus MA, Alhammadi AH, Razig EA, Alnaimi L. Pediatricians' perceptions of clinical practice guidelines. J Multidiscip Healthc. 2014;7:349–54.

12. Osterholt DM, Onikpo F, Lama M, Deming MS, Rowe AK. Improving pneumonia case-management in Benin: a randomized trial of a multi-faceted intervention to support health worker adherence to Integrated Management of Childhood Illness guidelines. Hum Resour Health. 2009;7(1):77.

13. Blacklock C, Gonçalves Bradley DC, Mickan S, Willcox M, Roberts N, Bergström A, et al. Impact of Contextual Factors on the Effect of Interventions to Improve Health Worker Performance in Sub-Saharan Africa: Review of Randomised Clinical Trials. PLoS One. 2016 Jan;11(1):e0145206.

14. Childhood Acute Illness and Nutrition Network. Childhood Acute Illness and Nutrition (CHAIN) Network: a protocol for a multi-site prospective cohort study to identify

For peer review only

modifiable risk factors for mortality among acutely ill children in Africa and Asia. BMJ Open. 2019 May;9(5):e028454.

15. Tickell KD, Mangale DI, Tornberg-Belanger SN, Bourdon C, Thitiri J, Timbwa M, et al. A mixed method multi-country assessment of barriers to implementing pediatric inpatient care guidelines. PLoS One. 2019;14(3):1–17.

16. Diallo AH, Sayeem Bin Shahid ASM, Khan AF, Saleem AF, Singa BO, Gnoumou BS, et al. Childhood mortality during and after acute illness in sub-Saharan Africa and South Asia - The CHAIN cohort study. medRxiv [Internet]. 2021 Jan 1;2021.11.24.21266806. Available from: http://medrxiv.org/content/early/2021/11/26/2021.11.24.21266806.abstract

17. Rutstein S. Steps to construct new DHS Wealth Index [Internet]. 2015. Available from: https://dhsprogram.com/programming/wealth index/Steps_to_constructing_the_new_DHS_Wealth_Index.pdf

18. WHO. Integrated Management of newborn and Childhood Illness Module: 1. An Introduction to the Integrated Management of Newborn and Childhood Illness (IMNCI). OpenLearnCreate. 2020. p. 1–8.

19. Azur MJ, Stuart EA, Frangakis C, Leaf PJ. Multiple imputation by chained equations: what is it and how does it work? Int J Methods Psychiatr Res. 2011 Mar;20(1):40–9.

20. Salih KEM, Bilal JA, Alfadeel MA, Hamid Y, Eldouch W, Elsammani E, et al. Poor adherence to the World Health Organization guidelines of treatment of severe pneumonia in children at Khartoum , Sudan. 2014;1–4.

21. Teixeira Rodrigues A, Roque F, Falcão A, Figueiras A, Herdeiro MT. Understanding

For peer review only

594 physician antibiotic prescribing behaviour: A systematic review of qualitative studies. Int
595 J Antimicrob Agents. 2013;41(3):203–12.

596 22. Dauncey JW, Olupot-Olupot P, Maitland K. Healthcare-provider perceptions of barriers to
597 oxygen therapy for paediatric patients in three government-funded eastern Ugandan
598 hospitals; A qualitative study. BMC Health Serv Res. 2019;19(1):1–9.

599 23. Wen B, Brals D, Bourdon C, Erdman L, Ngari M, Chimwezi E, et al. Predicting the risk of
600 mortality during hospitalization in sick severely malnourished children using daily
601 evaluation of key clinical warning signs. BMC Med. 2021;19(1):1–14.

602 24. Mvalo T. Need for routine pulse oximetry and oxygen delivery systems in low-income
603 and middle-income countries. Lancet Glob Heal [Internet]. 2022;10(3):e301–2. Available
604 from: http://dx.doi.org/10.1016/S2214-109X(22)00025-0

605 25. Gathara D, English M, van Hensbroek MB, Todd J, Allen E. Exploring sources of
606 variability in adherence to guidelines across hospitals in low-income settings: A multi-
607 level analysis of a cross-sectional survey of 22 hospitals. Implement Sci. 2015;10(1):1–8.

608 26. Biswas D, Hossin R, Rahman M, Bardosh KL, Watt MH, Zion MI, et al. An ethnographic
609 exploration of diarrheal disease management in public hospitals in Bangladesh: From
610 problems to solutions. Soc Sci Med. 2020;260(June):113185.

611 27. Rhee C, Aol G, Ouma A, Audi A, Muema S, Auko J, et al. Inappropriate use of antibiotics
612 for childhood diarrhea case management - Kenya, 2009-2016. BMC Public Health.
613 2019;19(Suppl 3):1–12.

614 28. Houston KA, Gibb JG, Maitland K. Intravenous rehydration of malnourished children
615 with acute gastroenteritis and severe dehydration: A systematic review. Wellcome open

For peer review only

Res [Internet]. 2017 Aug 18;2:65. Available from: https://pubmed.ncbi.nlm.nih.gov/28944301

29. De Bruycker M, Van den Bergh R, Dahmane A, Khogali M, Schiavetti B, Nzomukunda Y, et al. Non-adherence to standard treatment guidelines in a rural paediatric hospital in Sierra Leone. Public Heal Action. 2013;3(2):118–24.

30. Warfa O, Njai D, Ahmed L, Admani B, Were F, Wamalwa D, et al. Evaluating the level of adherence to Ministry of Health guidelines in the management of severe acute malnutrition at Garissa Provincial General Hospital, Garissa, Kenya. Pan Afr Med J. 2014 Mar;17:214.

For peer review only

626

For peer review only

STROBE Statement—Checklist of items that should be included in reports of ***cross-sectional studies***

	Item No	Recommendation	Page No
**Title and abstract**	1	(*a*) Indicate the study's design with a commonly used term in the title or the abstract	1
		(*b*) Provide in the abstract an informative and balanced summary of what was done and what was found	6
**Introduction**			
Background/rationale	2	Explain the scientific background and rationale for the investigation being reported	9-10
Objectives	3	State specific objectives, including any prespecified hypotheses	9
**Methods**			
Study design	4	Present key elements of study design early in the paper	10
Setting	5	Describe the setting, locations, and relevant dates, including periods of recruitment, exposure, follow-up, and data collection	10
Participants	6	(*a*) Give the eligibility criteria, and the sources and methods of selection of participants	10
Variables	7	Clearly define all outcomes, exposures, predictors, potential confounders, and effect modifiers. Give diagnostic criteria, if applicable	11-12
Data sources/ measurement	8*	For each variable of interest, give sources of data and details of methods of assessment (measurement). Describe comparability of assessment methods if there is more than one group	11-12
Bias	9	Describe any efforts to address potential sources of bias	13
Study size	10	Explain how the study size was arrived at	15
Quantitative variables	11	Explain how quantitative variables were handled in the analyses. If applicable, describe which groupings were chosen and why	13
Statistical methods	12	(*a*) Describe all statistical methods, including those used to control for confounding	13
		(*b*) Describe any methods used to examine subgroups and interactions	14
		(*c*) Explain how missing data were addressed	13
		(*d*) If applicable, describe analytical methods taking account of sampling strategy	N/A
		(*e*) Describe any sensitivity analyses	14
**Results**			
Participants	13*	(a) Report numbers of individuals at each stage of study—eg numbers potentially eligible, examined for eligibility, confirmed eligible, included in the study, completing follow-up, and analysed	15
		(b) Give reasons for non-participation at each stage	N/A
		(c) Consider use of a flow diagram	N/A
Descriptive data	14*	(a) Give characteristics of study participants (eg demographic, clinical, social) and information on exposures and potential confounders	15
		(b) Indicate number of participants with missing data for each variable of interest	13
Outcome data	15*	Report numbers of outcome events or summary measures	15-20

For peer review only - http://bmjopen.bmj.com/site/about/guidelines.xhtml

Main results	16	(*a*) Give unadjusted estimates and, if applicable, confounder-adjusted estimates and their precision (eg, 95% confidence interval). Make clear which confounders were adjusted for and why they were included	22-23
		(*b*) Report category boundaries when continuous variables were categorized	N/A
		(*c*) If relevant, consider translating estimates of relative risk into absolute risk for a meaningful time period	N/A
Other analyses	17	Report other analyses done—eg analyses of subgroups and interactions, and sensitivity analyses	19
**Discussion**			
Key results	18	Summarise key results with reference to study objectives	24
Limitations	19	Discuss limitations of the study, taking into account sources of potential bias or imprecision. Discuss both direction and magnitude of any potential bias	27
Interpretation	20	Give a cautious overall interpretation of results considering objectives, limitations, multiplicity of analyses, results from similar studies, and other relevant evidence	23-27
Generalisability	21	Discuss the generalisability (external validity) of the study results	26
**Other information**			
Funding	22	Give the source of funding and the role of the funders for the present study and, if applicable, for the original study on which the present article is based	29

For peer review only

BMJ Open

Prevalence and correlates of pediatric guideline non-adherence for initial empiric care in six low and middle-income settings: a hospital based cross sectional study

Journal:	*BMJ Open*
Manuscript ID	bmjopen-2023-078404.R1
Article Type:	Original research
Date Submitted by the Author:	02-Jan-2024
Complete List of Authors:	Shawon , R.A; University of Washington,<br>Denno, Donna; University of Washington<br>Tickell, Kirkby; University of Washington, Global Health; Childhood Acute Illness and Nutrition Network,<br>Atuhairwe, Michael; Makerere University<br>Bandsma, Robert; Centre for Global Child Health<br>Mupere, Ezekiel ; Makerere University, Paediatrics and Child Health<br>Voskuijl, Wieger ; Amsterdam University Medical Centres, Pediatrics<br>Mbale, Emmie; University of Malawi, Department of Pediatrics<br>Ahmed, Tahmeed; International Centre for Diarrhoeal Disease Research Bangladesh<br>Chisti, Md.; International Centre for Diarrhoeal Disease Research Bangladesh<br>Saleem, Ali; Aga Khan University Hospital<br>Ngari, Moses; Clinical Trials Facility<br>DIALLO, Hama; Universite Joseph Ki-Zerbo<br>Berkley, James ; KEMRI/Wellcome Trust Research Programme, Clinical Research<br>Walson, Judd; University of Washington<br>Means, Arianna; University of Washington
**Primary Subject Heading**:	Evidence based practice
Secondary Subject Heading:	Global health, Paediatrics, Public health, Infectious diseases
Keywords:	PAEDIATRICS, INFECTIOUS DISEASES, PUBLIC HEALTH

SCHOLARONE™
Manuscripts

I, the Submitting Author has the right to grant and does grant on behalf of all authors of the Work (as defined in the below author licence), an exclusive licence and/or a non-exclusive licence for contributions from authors who are: i) UK Crown employees; ii) where BMJ has agreed a CC-BY licence shall apply, and/or iii) in accordance with the terms applicable for US Federal Government officers or employees acting as part of their official duties; on a worldwide, perpetual, irrevocable, royalty-free basis to BMJ Publishing Group Ltd ("BMJ") its licensees and where the relevant Journal is co-owned by BMJ to the co-owners of the Journal, to publish the Work in this journal and any other BMJ products and to exploit all rights, as set out in our licence.

The Submitting Author accepts and understands that any supply made under these terms is made by BMJ to the Submitting Author unless you are acting as an employee on behalf of your employer or a postgraduate student of an affiliated institution which is paying any applicable article publishing charge ("APC") for Open Access articles. Where the Submitting Author wishes to make the Work available on an Open Access basis (and intends to pay the relevant APC), the terms of reuse of such Open Access shall be governed by a Creative Commons licence – details of these licences and which Creative Commons licence will apply to this Work are set out in our licence referred to above.

Other than as permitted in any relevant BMJ Author's Self Archiving Policies, I confirm this Work has not been accepted for publication elsewhere, is not being considered for publication elsewhere and does not duplicate material already published. I confirm all authors consent to publication of this Work and authorise the granting of this licence.

For peer review only

TITLE

Prevalence and correlates of pediatric guideline non-adherence for initial empiric care in six low and middle-income settings: a hospital based cross sectional study

AUTHORS

Riffat Ara Shawon, MPH[1,2]
Donna M. Denno, MD[1,3,4]
Kirkby D. Tickell, PhD[1,2,4]
Michael Atuhairwe, MBChB [4,8]
Robert H J Bandsma, PhD[4,13]
Ezekiel Mupere, PhD[4,8,9]
Wieger Voskuijl, PhD[4,10,11,12]
Emmie Mbale, MBBS[4, 14]
Tahmeed Ahmed, PhD[15]
Md. Jobayer Chisti, PhD[15]
Ali Faisal Saleem, MBBS[16]
Moses Ngari, PhD[5]
Abdoulaye Hama Diallo, PhD[17]
James A. Berkley, FRCPCH[4,5,6]
Judd L. Walson, MD[1,2,3,4,7]
Arianna Rubin Means, PhD[1]

CORRESPONDING AUTHOR INFORMATION

Name of the Corresponding Author: Riffat Ara Shawon

Affiliations:

1. Department of Global Health, University of Washington, Seattle, WA, USA.
2. Department of Epidemiology, University of Washington, Seattle, WA, USA.

Email Address: riasha47@gmail.com; shawon@uw.edu

For peer review only

AUTHOR AFFILIATIONS:

1. Department of Global Health, University of Washington, Seattle, WA, USA.
2. Department of Epidemiology, University of Washington, Seattle, WA, USA.
3. Departments of Pediatrics, University of Washington, Seattle, WA, USA.
4. The Childhood Acute Illness & Nutrition (CHAIN) Network, Nairobi, Kenya.
5. KEMRI/Wellcome Trust Research Programme, Kilifi, Kenya.
6. Centre for Tropical Medicine & Global Health, Nuffield Department of Medicine, University of Oxford, Oxford, UK.
7. Department of Medicine, University of Washington, Seattle, WA, USA.
8. Uganda-Case Western Reserve University Research Collaboration, Makerere University, Kampala, Uganda.
9. Department of Paediatrics and Child Health School of Medicine College of Health Sciences, Makerere University.
10. Amsterdam Centre for Global Child Health, Emma Children's Hospital, Amsterdam University Medical Centres.
11. Amsterdam Institute for Global Health and Development, Amsterdam University Medical Centres.
12. Kamuzu University for Health Sciences (formerly College of Medicine), Blanytre, Malawi.
13. Hospital for Sick Children, Translational Medicine Program and Centre for Global Child Health, Toronto, Canada.
14. Department of Pediatrics, College of Medicine, University of Malawi, Blantyre, Malawi.
15. International Centre for Diarrhoeal Disease Research, Bangladesh (icddr,b), Mohakhali, Dhaka, Bangladesh.
16. Aga Khan University Hospital, Karachi, Sindh, Pakistan.
17. University Joseph KI-ZERBO, Ouagadougou, Burkina Faso.

For peer review only

FULL ADDRESSES OF AUTHORS

Riffat Ara Shawon
Address: Hans Rosling Center for Population Health, Box 351620
3980 15th Ave NE, Seattle, WA 98105
email: shawon@uw.edu

Donna M. Denno
Address: Hans Rosling Center for Population Health, Box 351620
3980 15th Ave NE, Seattle, WA 98105
email: ddenno@uw.edu

Kirkby D. Tickell
Address: Hans Rosling Center for Population Health, Box 351620
3980 15th Ave NE, Seattle, WA 98105
email: kirkbt@uw.edu

Michael Atuhairwe, MBChB
Address: Uganda-Case Western Reserve University Research Collaboration, Makerere University, Upper Mulago Hill Road. Box 7072 Kampala, Uganda
email: michaelatu@gmail.com

Robert H J Bandsma, PhD
Address: Hospital for Sick Children, 555 University Avenue, Toronto, Ontario, Canada
email: robert.bandsma@sickkids.ca

Ezekiel Mupere, PhD
Address: Uganda-Case Western Reserve University Research Collaboration, Makerere University, Upper Mulago Hill Road. Box 7072 Kampala, Uganda.
Email: mupez@yahoo.com

Wieger P. Voskuijl, PhD
Address: Meibergdreef 9, 1105 AZ Amsterdam, Netherlands

For peer review only

email: w.p.voskuijl@amsterdamumc.nl

Emmie Mbale, MBBS
Address: University of Malawi, Chirunga Road, Zomba, Malawi
email: emmiembale@gmail.com

Tahmeed Ahmed. PhD
Address: Dhaka Hospital, Nutrition and Clinical Services Division, icddr,b,
Dhaka, Bangladesh
email: tahmeed@icddrb.org

Md. Jobayer Chisti, PhD
Address: Dhaka Hospital, Nutrition and Clinical Services Division, icddr,b,
Dhaka, Bangladesh
email: chisti@icddrb.org

Ali Faisal Saleem, MBBS
Address: Aga Khan University Hospital, National Stadium Road, Bahadurabad, Karachi, Sindh, Pakistan.
email: ali.saleem@aku.edu

Moses Ngari
Address: KEMRI/Wellcome Trust Research Programme, Clinical Trials Facility, Hospital road, Kilifi, 80108, Kenya
email: mngari@kemri-wellcome.org

Abdoulaye Hama Diallo
Address: Avenue Charles De Gaulle, Ouagadougou, Burkina Faso
email: hamadial@yahoo.fr

James A. Berkley
Address: Oxford OX1 2JD, United Kingdom
email: jberkley@kemri-wellcome.org

For peer review only

Judd L. Walson
Address: Hans Rosling Center for Population Health, Box 351620
3980 15th Ave NE, Seattle, WA 98105
email: walson@uw.edu

Arianna Rubin Means, PhD
Address: Hans Rosling Center for Population Health, Box 351620
3980 15th Ave NE, Seattle, WA 98105
email: aerubin@uw.edu

For peer review only

ABSTRACT

Objectives: This study evaluated the prevalence and correlates of guideline non-adherence for common childhood illnesses in low-resource settings.

Design and setting: We used secondary cross-sectional data from eight healthcare facilities in six Asian and African countries.

Participants: A total of 2,796 children aged 2-23 months hospitalized between November 2016, and January 2019 with pneumonia, diarrhea, or severe malnutrition and without HIV infection were included in this study.

Primary outcome measures: We identified children treated with full, partial, or non-adherent initial inpatient care according to site-specific standard-of-care guidelines for pneumonia, diarrhea, and severe malnutrition (SM) within the first 24-hours of admission. Correlates of guideline non-adherence were identified using generalized estimating equations.

Results: Fully adherent care was delivered to 32% of children admitted with diarrhea, 34% of children with pneumonia, and 28% of children with SM when a strict definition of adherence was applied. Non-adherence to recommendations was most common for oxygen and antibiotics for pneumonia; fluid, zinc, and antibiotics for diarrhea; and vitamin A and zinc for SM. Non-adherence varied by site. Pneumonia guideline non-adherence was more likely among patients with severe disease (Odds Ratio [OR]:1·82; 95% Confidence Interval [CI]:1·38, 2·34) compared to non-severe disease. Diarrhea guideline non-adherence was more likely among lower asset quintile groups (OR:1·16; 95% CI:1·01, 1·35), older children (OR:1·10; 95% CI:1·06, 1·13) and children presenting with wasting (OR:6·44; 95% CI:4·33, 9·57) compared to those with higher assets, younger age, and not wasted.

For peer review only

Conclusions: Non-adherence to pediatric guidelines was common and associated with older age, disease severity, and comorbidities, and lower household economic status. These findings highlight opportunities to improve guidelines by adding clarity to specific recommendations.

For peer review only

STRENGTHS AND LIMITATIONS OF THIS STUDY

- To our knowledge, this is the largest multi-country study conducted to date evaluating the prevalence of guideline non-adherence for three major causes of pediatric mortality and their sub-conditions: pneumonia, diarrhea, and severe malnutrition.
- Strict interpretation of guidelines with the support of a clinical advisory group revealed contradictions, complexities and gaps within guidelines, including challenges in applying guidelines when children have multiple comorbidities.
- This study uniquely investigated patient-level (ex. socioeconomic status), provider-level (ex. clinician perceived risk at admission that the child may die during hospitalization), and facility-level correlates (ex. patient arrival during weekdays vs weekends/holidays) of guideline non-adherence.
- Non-adherence in this study was measured in hospitals with highly trained and monitored staff which may not be generalizable in all settings.
- Observed non-adherence could be driven by purposeful deviations from guidelines, resulting from resource limitations, environmental factors (e.g., local antibiotic resistance pattern) and vague or unclear guidelines. It was not possible to differentiate purposeful and accidental guideline non-adherence.

For peer review only

INTRODUCTION

Globally, an estimated 14,000 under-five children die every day.[1] Leading causes of mortality in the post-neonatal period include pneumonia (13%) and diarrhea (8%), and undernutrition is an underlying cause in nearly half of child deaths under the age of 5 years.[2,3] Low-resource settings bear a disproportionate burden of child mortality, with over 50% of these deaths occurring in sub-Saharan Africa and one-third in South Asia.[4]

World Health Organization (WHO) normative guidelines offer informed decision support for managing undernutrition, diarrhea, pneumonia and other conditions. These guidelines are often adapted to national contexts for adoption at the country-level. When applied with high fidelity, clinical guidelines can improve health outcomes of targeted populations and reduce case fatality.[5] Yet, guidelines for reducing mortality in children under five, such as the Integrated Management of Childhood Illness and other related WHO guidelines, are inconsistently applied.[6] Guideline non-adherent case management can be driven by inadequate assessment, treatment, and monitoring, local clinical culture, limitations in infrastructure, supply chain challenges, political context, differences in patient-practitioner goals, disease trajectory, patient comorbidities and clarity and applicability of recommendations. Thus, in some circumstances guideline non-adherence may be clinically justified. [7–11]

Evidence from multiple settings indicates that training alone does not affect adherence to guidelines.[12] However, there is minimal information regarding the patient, health service provider and institution specific factors that influence fidelity to clinical guidelines in low-and-middle-income countries (LMICs).[13] We aim to identify the frequency of guideline non-adherence and factors influencing non-adherence during inpatient pediatric treatment for pneumonia, diarrhea, and severe malnutrition (SM) across eight facilities in six LMICs. This

For peer review only

information will be helpful in identifying specific opportunities to support healthcare workers in their daily patient care.

METHODS

Study setting

This analysis utilized de-identified data from the Childhood Acute Illness and Nutrition (CHAIN) study, a multi-site prospective cohort study that identifies risk factors for mortality in hospitalized acutely ill children and after discharge.[14] The CHAIN study took place between November 2016, and January 2019. We include children enrolled in CHAIN aged 2-23 months, hospitalized with pneumonia, diarrhea, or SM (referred to within the guidelines as severe acute malnutrition or SAM) and without HIV infection. Children with malnutrition were deliberately oversampled in CHAIN.

Urban CHAIN sites included hospitals in Karachi (Pakistan), Dhaka (Bangladesh), Kampala (Uganda), Blantyre (Malawi), and Nairobi (Kenya), and rural sites were in Banfora (Burkina Faso), Migori (Kenya), and Matlab (Bangladesh). The sites included public, private non-profit, referral, and district hospitals and often receive patients with complex or severe disease compared to lower-level facilities. Further information about hospital characteristics can be found elsewhere.[15] One CHAIN site, Kilifi County Hospital, Kenya, was omitted from the analysis, as there was a potential conflict between this analysis and ongoing internal guideline adherence auditing processes. Participating Bangladesh facilities were diarrhea specialty centers, also admitting children with other conditions such as pneumonia and SM. In the CHAIN study, sites received a pre-study resource audit, and ongoing performance audit and feedback. Sites were also supported by trained

For peer review only

research clinicians to ensure high standards of clinical care.[16] Thus, in this secondary analysis, we assume clinical staff were highly trained and aware of ongoing monitoring.

Key informants from each site identified institutional or national guidelines available for each condition, and if none, WHO guidelines were used for this analysis (Supplemental Table 1). All sites within the same country reported using the same guidelines. Some facilities might draw from multiple guidelines to manage a child, but for the purposes of this analysis a single and most primary guideline was used as the standard-of-care to abstract relevant diagnostic criteria and treatment recommendations for each condition within each site. To identify children with pneumonia, diarrhea, SM, and sub-conditions we used CHAIN records of clinician diagnoses or, if necessary, we applied guideline diagnostic criteria (Supplemental Table 1).

Conditions and sub-conditions

We evaluated guideline adherence for initial treatment of pneumonia, diarrhea, and SM, the most common conditions across all sites. We also assessed adherence to sub-conditions, including non-severe and severe pneumonia, acute watery diarrhea (AWD) with severe, some and no dehydration, non-severe and severe persistent diarrhea, dysentery, and SM complicated by hypoglycemia, hypothermia, dehydration, severe anemia, measles, micronutrient deficiency, infection, and skin lesions.

We reviewed diagnoses and initial management provided within the first 24-hours of admission. However, we reviewed and counted antibiotic management up to 48-hours to allow for emergence of new clinical information.

For peer review only

Defining adherence to guidelines

We formed a clinical advisory group comprising three CHAIN clinicians, two pediatricians and one general practitioner, simulating a real-world scenario of clinical decision-making with peer support. The group met four times and discussed complex clinical scenarios (e.g., a child with both non-severe pneumonia and SM) to resolve ambiguity guideline interpretation when needed and to allow flexibility in situations of multi-morbidities with conflicting antibiotic recommendations. For example, if a child had both non-severe pneumonia and SM, treatment with broad-spectrum injectable antibiotics (e.g., ampicillin and gentamicin) recommended for SM was considered guideline adherent for non-severe pneumonia, even though pneumonia guidelines recommended an oral antibiotic (e.g., amoxicillin).

Diarrhea, pneumonia, and SM guideline adherence was calculated as the proportion of children initially treated with adherent care for all relevant sub-conditions within 24-hours of admission. We reported the site-specific number and proportion of children managed with full, partial, or non-adherent care. Fully adherent care included providing all treatment components in guidelines (e.g., recommended antibiotics, oral and IV fluid, therapeutic feeds). Non-adherent care indicated that a child was not provided any recommended treatments. Partial adherence indicated that some, but not all, guideline-indicated treatments were provided. We also reported adherence to specific recommendations (e.g., oxygen supplementation for severe pneumonia). The analysis was agnostic to the relative importance of different recommendations.

Statistical analysis

For peer review only

Descriptive statistics were applied to report the proportion of adherence to treatment for conditions (full, partial, and non-adherence) and sub-conditions (adherence and non-adherence) within each site and overall.

The dichotomous outcome for the correlates analysis was any non-adherence to guidelines (including partial non-adherence). We explored patient-specific correlates including age, sex, socio-economic status (composite variable standardized using asset quintiles, derived from the Demographic and Health Survey wealth index), pre-admission care-seeking history , and wasting (weight-for-height Z score <-2).[17] Other patient-specific correlates included comorbidities and presence of WHO and condition-specific danger signs (i.e., obstructed breathing, respiratory distress, shock, cyanosis, convulsions, severe anemia, severe dehydration, profuse watery diarrhea, severe vomiting, impaired consciousness) as assessed by study clinicians.[18] Provider-specific correlates included clinician perceived risk at admission that the child may die during hospitalization. Facility-specific correlates included patient arrival during weekdays vs weekends/holidays according to each country's holiday and weekend conventions.

We determined the association between the pre-specified correlates of interest and guideline non-adherence for each condition by utilizing generalized estimating equations (GEE) with a logit link, with clustering at the hospital-level, robust standard error estimates and an exchangeable correlation structure. This multivariable analysis was evaluated at a 5% alpha level.

Two independent variables had some missingness: care-seeking history (~2% missingness) and clinician risk impression upon admission (~0·1% missingness). We used multiple imputation by chained equation for these variables under the assumption of data missing at random using all non-missing exposure variables in the imputation model. Rubin's rule was used to combine logistic regression outputs of ten iterations.[19] In sensitivity analysis, we repeated all analyses with

For peer review only

complete cases. Because the results did not substantially differ between the two approaches, we presented results from the imputed data.

Kenya's pneumonia guideline excluded children with SM from its treatment recommendations because of conflicting antibiotic recommendations in the national guidelines. Because antibiotic recommendations often conflict between SM and pneumonia recommendations, especially for non-severe pneumonia, we conducted a sensitivity analysis in which we excluded children with SM from pneumonia-specific analyses for all countries. Furthermore, all diarrhea guidelines indicated that diarrhea with comorbid SM requires special management, especially if the child has dehydration. Per the guidelines, SM cases were excluded when calculating diarrhea guideline adherence for all diarrhea sub-conditions. Adherence to rehydration recommendations for children with SM, diarrhea and dehydration was assessed under the SM with dehydration sub-condition.

Because SM assessment in children >6 months does not account for low birth weight or prematurity, we calculated SM guideline adherence amongst children <6 months versus ≥6 months in additional sensitivity analysis. Finally, we conducted a sensitivity analysis for conditions and sub-conditions without any comorbidities overall across all sites. Stata v.16 was used for all analyses.

Role of the funding source

The funding sources had no role in study design, in the collection, analysis, and interpretation of data, in the writing of or decision to submit the report for publication.

Patient and public involvement

Patients or the public were not involved in the design, conduct, reporting, or dissemination plans of our research.

For peer review only - http://bmjopen.bmj.com/site/about/guidelines.xhtml

RESULTS

Sample characteristics

Diagnosis and treatment criteria were drawn from thirteen different guidelines (Supplemental Tables 1 & 2) and applied to 2,796 pediatric hospitalizations. A quarter of patient admissions were in Bangladesh (n=708). More than half were male (57%) and 42% were aged 12-23 months (Table 1).

Table 1: Demographic characteristics and prevalence of pneumonia, diarrhea and SM

	Total (N=2,796)	Bangladesh (n=708)	Burkina Faso (n=429)	Kenya (n=533)	Malawi (n=311)	Pakistan (n=349)	Uganda (n=466)
	N (%)[1]	n (%)[1]	n (%)[1]	n (%)[1]	n (%)[1]	n (%)[1]	n (%)[1]
Demographics of children							
Age at admission (months)							
2-5	561 (20)	164 (23)	66 (15)	122 (23)	54 (17)	106 (30)	49 (11)
6-11	1058 (37)	313 (44)	127 (30)	191 (36)	104 (33)	122 (35)	201 (43)
12-23	1177 (42)	231 (33)	236 (55)	220 (41)	153 (49)	121 (35)	216 (46)
Male sex	1588 (57)	426 (60)	245 (57)	297 (56)	172 (55)	190 (54)	258 (55)
Assets Quintiles							
1st (lowest)	464 (17)	58 (8)	141 (33)	155 (29)	92 (30)	1 (0·3)	17 (4)
2nd	538 (19)	106 (15)	183 (43)	71 (13)	91 (29)	4 (0·7)	83 (18)
3rd	582 (21)	140 (20)	72 (17)	113 (21)	59 (19)	30 (9)	168 (36)
4th	596 (21)	187 (26)	19 (4)	111 (21)	34 (11)	107 (31)	138 (30)
5th (highest)	616 (22)	217 (31)	14 (3)	83 (16)	35 (11)	207 (59)	60 (13)
Conditions and sub-conditions							
Pneumonia	1095 (39)	279 (39)	101 (24)	306 (57)	74 (24)	192 (55)	143 (31)
Non-severe	296 (10)	138 (19)	23 (5)	52 (10)	27 (9)	34 (10)	22 (5)
Severe	799 (29)	141 (20)	78 (18)	254 (48)	47 (15)	158 (45)	121 (26)
Diarrhea[2]	935 (32)	576 (81)	71 (16)	103 (20)	60 (20)	49 (14)	76 (16)
AWD w/ severe dehydration	94 (3)	31 (4)	21 (5)	2 (0·4)	10 (3)	11 (3)	19 (4)
AWD w/ some dehydration	130 (5)	50 (7)	5 (1)	46 (9)	5 (2)	10 (3)	14 (3)
AWD w/ no dehydration	683 (23)	494 (70)	45 (10)	54 (10)	40 (13)	16 (5)	34 (7)

For peer review only

For peer review only - http://bmjopen.bmj.com/site/about/guidelines.xhtml

Persistent[3]	14 (0·5)		-	-	3 (1)	5 (1)	6 (1)
Severe persistent[3]	8 (0·3)		-	-	2 (1)	4 (1)	2 (0·4)
Dysentery	6 (0·2)	1 (0·1)	-	1 (0.1)	-	3 (0·9)	1 (0·2)
SM[4]	1096 (39)	269 (38)	181 (42)	218 (41)	83 (27)	118 (34)	227 (49)
w/ hypoglycemia	23 (2)	2 (0·3)	5 (1)	6 (1)	-	3 (0·9)	7 (2)
w/ hypothermia	10 (0·9)	-	-	4 (0·8)	2 (0·6)	-	4 (0·9)
w/ dehydration[5]	174 (16)	76 (11)	17 (4)	20 (4)	43 (14)	16 (5)	2 (0·4)
w/ measles	17 (2)	2 (0·3)	-	-	-	8 (2)	7 (2)
w/ severe anemia	31 (3)	-	27 (6)	3 (0·6)	-	1 (0·3)	-
w/ skin lesions	337 (31)	57 (8)	35 (8)	72 (14)	15 (5)	50 (14)	109 (23)
Conditions with Comorbidities[6]							
Pneumonia w/ other comorbidities	905 (32)	253 (36)	96 (22)	228 (43)	54 (17)	157 (45)	116 (25)
Diarrhea w/ other comorbidities	702 (25)	437 (62)	70 (16)	78 (15	30 (10)	41 (12)	46 (10)
SM w/ other comorbidities	996 (36)	267 (38)	168 (39)	206 (39)	68 (22)	115 (33)	172 (37)

[1] The denominator for all percentages is the total number of children treated in each site
[2] Diarrhea with dehydration in children with SM is included under SM
[3] Persistent diarrhea involves no signs of dehydration and severe persistent diarrhea involves signs of dehydration
[4] SM sub conditions are not mutually exclusive. Also, guidelines assume that all SM cases have infection, micronutrient deficiency, and electrolyte imbalance and should be treated accordingly. The other sub-conditions listed in the table for SAM may or may not be present and are not mutually exclusive
[5] Excludes SM with shock/unconsciousness based on guideline recommendations
[6] Comorbidities: bronchiolitis, lower respiratory tract infection, upper respiratory tract infections, tuberculosis, otitis media, asthma, anemia, sickle cell disease, thalassemia, nephrotic syndrome, nephritis, other renal diseases, liver disease, ileus, cardiac disease, sepsis, malaria, soft tissue disease, urinary tract infection, measles, varicella, osteomyelitis, febrile illness, enteric fever, epilepsy, meningitis, encephalopathy, hydrocephalus, developmental delay, cerebral palsy, gastroenteritis, SM (excluding the condition originally being assessed as applicable)
Notes: (1) AWD= Acute Watery Diarrhea, (2) Columns percentages may not exactly be 100% due to rounding, (3) Decimals are rounded to the nearest integer except when the proportion is less than 1%, (4) No exclusions are made in this table for prevalence of conditions and sub-conditions unless noted otherwise, (5) A “-” indicates that there were no cases in the sample with the diagnosis (6) A blank cell indicates there are no specific recommendations for the given condition in the country

321

322 *Prevalence of conditions and sub-conditions*

323 Approximately one-third of children were admitted with severe pneumonia and 10% with non-
324 severe pneumonia (Table 1). Over half in Kenya (57%, n=533) and Pakistan (55%, n=349) were
325 admitted with pneumonia. About 32% of children across facilities were admitted with diarrhea
326 (excluding children with concurrent SM). The Bangladesh facilities, being diarrhea specialty

For peer review only

hospitals, had the highest proportion of children admitted with diarrhea (81%, n=576) while Pakistan had the lowest (14%, n=349). Across facilities, 39% of children had SM, which is expected given that CHAIN purposefully overenrolled children with wasting. Approximately one-third of children were admitted with at least two of the three conditions: diarrhea, pneumonia and SM. Supplemental Table 3 provides more detail about comorbidities in the sample.

Prevalence of guideline adherence

The guidelines used by countries have significant differences (Supplemental table 1). Prevalence of guideline adherence varied by country and guideline recommendations. Overall, 42% of children with pneumonia were treated with fully adherent care (Table 2 and Supplemental Table 4). The majority of the non-severe pneumonia (54%) cases were treated with full adherence, whereas only 38% of children with severe pneumonia were treated with full adherence. Lower adherence to pneumonia guidelines was driven primarily by non-provision of oxygen when recommended and prescription of different antibiotics, typically broader spectrum, from those recommended (e.g., ceftriaxone rather than ampicillin and gentamicin) (Table 3 and Supplemental Table 5). In sensitivity analyses in which comorbid SM was excluded, we continued to observe partial adherence in most pneumonia cases (58%) (Supplemental Table 6).

Table 2: Summary of guideline adherence, by conditions and sub-conditions

Subtitle: Cell colors indicate which adherence level applied to the largest proportion of patients, by condition and site. Light blue indicates a majority of patients were treated with full adherence, medium blue indicates a majority of patients were treated with partial adherence, and dark blue indicates a majority of patients were treated with non-adherent care

Conditions and sub-conditions	Bangladesh	Burkina Faso	Kenya	Malawi	Pakistan	Uganda	Overall
PNEUMONIA[1]							
Pneumonia (combined)							
Non-severe							

For peer review only

Severe							
DIARRHEA[2]							
Diarrhea (combined)							
AWD w/ severe dehydration							
AWD w/ some dehydration				5a			
AWD w/ no dehydration							
Persistent diarrhea		-	5b				
Severe persistent diarrhea		-	-	5a			
Dysentery		-		-			
SM							
SM (combined)							
W/ hypoglycemia[3]				-			
W/ hypothermia	-	-		5b	-		
W/ dehydration			5b		5b	5b	
W/ severe anemia	-			-			
W/ measles		-	-				
W/ micronutrient deficiency[4] (all SM)							
W/ infection[4] (all SM)							
Requiring nutritional stabilization[4] (all SM)							
W/ skin lesion	-						

[1] For pneumonia, comorbid SM was excluded as per Kenyan pneumonia treatment guidelines
[2] For diarrhea, comorbid SM was excluded, per guidelines
[3] Hypoglycemia treatment applies to all children with SM irrespective of serum glucose level in Uganda, Kenya, Pakistan and Bangladesh, per guidelines
[4] Per guidelines, all children with SM should be treated for presumptive infection, micronutrient deficiency and require nutritional stabilization. Micronutrients include Vit A, zinc, folate, potassium, magnesium
[5a] The proportion of patients treated with partially adherent and nonadherent care were equally high, and the cell is coded to partial adherence
[5b] The propostion of patients treated with fully adherent and nonadherent care were equally high, and the cell is coded to full adherence
Notes: (1) AWD= Acute Watery Diarrhea (2) A "-" indicates that there were no children with the diagnosis (3) a blank cell indicates there are no specific recommendations for a condition in the country, (4) Supplemental Table 4 provides detailed values and proportions for each cell

Across all sites, only 32% children with diarrhea were treated with fully guideline adherent care (Supplemental Table 4). Bangladesh and Burkina Faso had the highest levels of full adherence

For peer review only

while Kenya, Pakistan and Uganda had the lowest (Table 2). The recommendation commonly exhibiting least adherence across diarrhea sub-conditions was provision of zinc (Table 3). Persistent diarrhea and dysentery were typically treated with partially adherent or non-adherent care across sites. Overall, 81% of children were treated will full adherence to the recommendation of withholding antibiotics for acute watery diarrhea without comorbidity. About 41% children with diarrhea were admitted with comorbidities, primarily SM, for which antibiotics were indicated.

Overall, most children with SM were treated with partially adherent care (72%) (Supplemental Table 4). Full adherence was observed in a minority of patients with hypothermia (20%), measles (20%), or skin lesions (5%). In contrast, most patients with SM and hypoglycemia (47%), dehydration (81%), severe anemia (81%), and infection (65%), were treated with fully adherent care. Nutritional stabilization was also started in most children with SM (79%). In sensitivity analysis, we found that full adherence to overall SM guidelines was higher in children 6 months and older (29%) compared to less than 6 months (19%). Recommendations were less detailed for <6 months and simply stated that management should be similar to >6 months old, except some feeding recommendations. Full adherence was lower in <6 months children compared to 6 months and older for recommendations to treat hypoglycemia (43 % vs 54%), dehydration (79% vs 82%), presumed micronutrient deficiency (35% vs 44%), and presumed infection (61% vs 65%). Fully adherent care was higher in <6 months versus in 6 months and older for treatment of skin lesion (15% vs 4%) and severe anemia (100% vs 79%). Adherence to nutritional stabilization recommendations did not differ between the age groups (79% fully adherent).

We conducted sensitivity analyses to describe overall guideline adherence patterns for pneumonia, diarrhea and SM without comorbidities (Supplemental Table 7) and found no appreciable

For peer review only

difference except lower adherence for non-severe pneumonia and higher adherence for micronutrients in SM with dehydration.

Table 3: Number and proportion of children treated with fully guideline adherent care, by specific guideline recommendations

Subtitle: Each cell indicates the number and proportion of children receiving fully adherent care. Cells colored in dark blue indicate <65% full adherence, blue indicate 65-80% full adherence and light blue indicate >80% full adherence

Specific recommendations	Bangladesh	Burkina Faso	Kenya	Malawi	Pakistan	Uganda	Overall
	n (%)						
PNEUMONIA[1]							
Antibiotics for severe pneumonia	107 (76)	36 (46)	102 (68)	42 (89)	0 (0)	98 (81)	329(52)
Antibiotics for non-severe pneumonia	101 (73)	8 (35)	5 (13)	12 (44)	17 (50)	16 (73)	129(54)
Oxygen for severe pneumonia		78 (100) [3]	71 (47) [4]	6 (13)[4]	158 (100) [3]	121 (100) [3]	434(78)
Paracetamol for fever (Severe Pneumonia)		74 (95)		47 (100)	156 (99)	119 (98)	396(98)
Paracetamol for fever (non-severe Pneumonia)				27 (100)		22 (100)	49 (100)
Bronchodilator for wheeze (severe pneumonia)		67 (86)	134 (89)	39 (83)	128 (81)	109 (90)	477(86)
Bronchodilator for wheeze (non-severe pneumonia)			36 (95)	20 (74)		20 (91)	76 (87)
DIARRHEA[2]							
Rehydration for AWD with severe dehydration	9 (29)	14 (67)	2 (100)	3 (30)	8 (73)	9 (47)	45 (52)
Zinc for AWD with severe dehydration	22 (71)	13 (62)	1 (50)	4 (40)	3 (27)	3 (16)	46 (49)
Rehydration for AWD with some dehydration	49 (98)	3 (60)	17 (37)	3 (60)	1 (10)	8 (57)	81(80)
Zinc for AWD with some dehydration		3 (60)	22 (48)	1 (20)	3 (30)	1 (7)	30 (28)
Rehydration for AWD with no dehydration	300 (96)	23 (51)	15 (28)	25 (63)	4 (25)	8 (24)	375 (81)
No antibiotic in AWD without comorbidity	291 (74)	70 (99)	82 (80)	43 (78)	33 (89)	44 (66)	651 (81)
Zinc for AWD with no dehydration		26 (58)	14 (26)	21 (53)	2 (12)	3 (9)	66 (14)
Rehydration for persistent diarrhea		-	2 (50)	2 (67)	3 (60)	0 (0)	7 (44)

For peer review only

Zinc for persistent diarrhea		-	2 (50)	1 (33)	1 (20)	0 (0)	4 (25)
Multi-vitamins for persistent diarrhea[5]		-		0 (0)		0 (0)	0 (0)
Microminerals[6] for persistent diarrhea		-		0 (0)			0 (0)
Rehydration for severe persistent diarrhea		-		1 (50)	3 (75)	0 (0)	4 (50)
Zinc for severe persistent diarrhea		-		0 (0)	0 (0)	0 (0)	3 (30)
Multi-vitamins for severe persistent diarrhea[5]		-		0 (0)		0 (0)	0 (0)
Microminerals[6] for severe persistent diarrhea		-		0 (0)			0 (100)
Rehydration for dysentery		-	0 (0)	-	2 (67)	0 (0)	2 (33)
Zinc for dysentery		-	0 (0)	-	1 (33)	0 (0)	1 (17)
Antibiotics for dysentery	0 (0)	-	1 (100)	-	0 (0)	1 (100)	2 (33)
SM							
Specific antibiotics in SM	236 (88)	41 (23)	161 (74)	62 (75)	2 (2)	206 (91)	710 (65)
Fluid in SM with dehydration	71 (93)	16 (94)	10 (50)	35 (81)	8 (50)	1 (50)	141 (33)
Blood transfusion in SM with severe anemia	-	21 (78)	3 (100)	-	1 (100)	-	25 (81)
Vitamin A in SM with measles		-	-		2 (25)	1 (14)	3 (20)
Micronutrients in SM	143 (53)	68 (38)	114 (52)		22 (19)	72 (32)	471(43)
Therapeutic food in SM	262 (97)	153 (85)	184 (84)	62 (74)	58 (49)	147 (65)	866 (79)
Zinc for skin lesion in SM					8 (16)	0 (0)	8 (5)

[1] For pneumonia, comorbid SM was excluded as per Kenyan pneumonia treatment guidelines
[2] For diarrhea, comorbid SM was excluded, per guidelines
[3] Oxygen is recommended for severe pneumonia with SpO2<90% only
[4] Oxygen is recommended to all children with severe pneumonia
[5] Vitamins include vitamin A, folate, both, and/or multivitamin
[6] Microminerals was an available variable in the CHAIN data without details of what the minerals are
Notes: (1) The denominator for all percentages is the total number of children with a specific condition, (2) AWD= Acute Watery Diarrhea, (3) A "-" indicates there were no cases in the sample with the diagnosis. (4) A blank cell indicates there are no relevant recommendation for a condition in the country, (5) Supplemental Table 5 provides detailed recommendation-specific estimates of adherence levels for all cases

383

384 *Correlates of guideline non-adherence*

385 In multivariable analysis, children with severe pneumonia were more likely to be treated with
386 guideline non-adherent care (OR:1·81; 95% CI:1·36, 2·41) (Table 4) compared to non-severe
387 pneumonia. For diarrhea, children who were older (OR:1·10; 95% CI:1·06, 1·43) and wasted at

For peer review only

admission (OR:6·69; 95% CI:4·53, 10·17) were more likely to be treated with guideline non-adherent care compared to younger or non-wasted children. Children from higher asset quintiles (OR:0·85; 95% CI:0·73, 0·99) were less likely to receive non-adherent care. For SM, none of the factors appeared to be statistically significant in this analysis.

Table 4: Correlates of pneumonia, diarrhea and SM guideline non-adherence

Variables	Multivariable analysis		
	OR	90% CI	p-value
**PNEUMONIA**			
Older age	0·98	0·96, 1·00	0·113
Male sex	0·85	0·66, 1·09	0·199
Wasted at admission	0·95	0·71, 1·26	0·706
Losing weight/not gaining weight problem detected at admission	0·80	0·58, 1·10	0·166
Presence of danger sign	1·12	0.81, 1·55	0·500
Severe Pneumonia[1]	1·81	1·36, 2·41	<0·001
Previous hospitalization	1·08	0·94, 1·26	0·28
Child likely to die[2]	1·15	0·82, 1·63	0·42
Asset quintile[3]	0·99	0·87, 1·11	0·77
Seeking prior treatment[4]			
Did not seek treatment	1·26	0·79, 2·02	0·332
Sought treatment from shop/pharmacy	1·02	0·76, 1·35	0·911
Sought treatment from traditional healer/herbalist/homeopathist	1·55	0·84, 2·88	0·164
Presence of comorbidity	1·08	0·76, 1·53	0·676
Referred to hospital by health worker	1·23	0·92, 1·66	0·169
Admitted on a holiday	0·87	0·57, 1·34	0·568
**DIARRHEA**			
Older age	1·10	1·06, 1·43	<0·001
Male sex	1·27	0·91, 1·77	0·155
Wasted at admission	6·79	4·53, 10·17	<0·001
Losing weight/not gaining weight problem detected at admission[a]	1·11	0·72, 1·71	0·649
Presence of danger sign	1·50	0·90, 2·50	0·117
Previous hospitalization	1·01	0·81, 1·25	0·933
Child likely to die[2]	0·85	0·54, 1·35	0·496
Asset quintile[3]	0·85	0·73, 0·99	0·033
Seeking prior treatment[4]			
Did not seek treatment	1·03	0·57, 1·89	0·900
Sought treatment from shop/pharmacy	0·91	0·62, 1·34	0·632
Sought treatment from traditional healer/herbalist/homeopathist	0·44	0·19, 1·06	0·066

For peer review only

Presence of comorbidity	0·87	0·61, 1·29	0·527
Referred to hospital by health worker	1·09	0·71, 1·67	0·709
Admitted on a holiday	0·86	0·55, 1·35	0·525
Diarrhea sub-condition[5]			
AWD w/ severe dehydration	1·70	0·96, 3·01	0·067
AWD w/ some dehydration	0·63	0·38, 1·06	0·085
**SM**			
Older age	0·98	0·94, 1·01	0·200
Male sex	1·41	0·93, 2·15	0·106
Losing weight/not gaining weight problem detected at admission	0·59	0·35, 1·01	0·056
Danger sign	0·64	0·35, 1·19	0·161
Previous hospitalization	1·05	0·82, 1·33	0·700
Child likely to die[2]	0·74	0·44, 1·26	0·270
Assets quintile[3]	1·07	0·88, 1·31	0·495
Presence of comorbidity	1·34	0·69, 2·62	0·390
Seeking prior treatment[4]			
Did not seek treatment	2·31	0·77, 7·00	0·137
Sought treatment from shop/pharmacy	1·39	0·80, 2·40	0·243
Sought treatment from traditional healer/herbalist/homeopathist	0·94	0·45, 1·99	0·875
Referred to hospital by health worker	0·84	0·53, 1·35	0·476
Admitted on a holiday	1·04	0·47, 2·31	0·919
SM w/ hypoglycemia	0·54	0·18, 1·59	0·263
SM w/ hypothermia	0·72	0·10, 5·16	0·747
SM w/ dehydration	2·49	1·01, 6·13	0·048
SM w/ skin lesion	1·46	0·89, 2·38	0·132

Reference categories: [1] non-severe pneumonia, [2]child unlikely to die, [3] lowest quintile, [4] sought treatment from a medical facility, [5] AWD w/ no dehydration

[a]Losing weight/not gaining weight problem detected at admission (detected by history taking) and admission MUAC group (detected by anthropometry measurement) were highly correlated. Admission MUAC group was used in the multivariable analysis as this is an objective measure

Notes: (1) AWD=Acute watery diarrhea; OR=Odds Ratio (2) There were not enough cases to run regressions with SM w/ measles and SM w/ severe anemia

DISCUSSION

We examined reported pediatric treatment data from eight health facilities across a range of geographic and ecologic settings in Africa and South Asia to examine adherence to standard-of-care treatment guidelines. Guidelines vary considerably across countries in level of detail and

For peer review only

scope of included recommendations. Overall, partial and non-adherence to pneumonia, diarrhea, and SM guidelines was common, although variable by country and across individual recommendations.

Pneumonia guideline adherence

Most children with pneumonia were treated with partial adherence. Lowest adherence was noted for antibiotic recommendations and oxygen supplementation guidance. While amoxicillin is recommended for non-severe pneumonia by all guidelines, broader spectrum antibiotics were typically prescribed in most cases. Hospitalization is not indicated for non-severe pneumonia alone, thus it was likely that children with non-severe pneumonia had other comorbidities or were perceived by a clinician to be at risk of deterioration. A study in Sudan found only 18% of hospitalized children with pneumonia received recommended antibiotic, and that broader spectrum antimicrobials were commonly prescribed.[20] A systematic review of qualitative studies revealed that previous clinical practice, peer practices, fear of patient complications, diagnostic uncertainty, patient comorbidities, desiring a rapid cure, time pressure, and cost saving influenced antibiotic prescription decisions.[21] Clinical guidelines did not emerge as a key driver of initial empiric antibiotic use in the review.[21]

Kenyan and Malawian guidelines include oxygen supplementation for all children with severe pneumonia; adherence to this recommendation was uncommon. In contrast, supplementation is recommended based on oxygen saturation levels in the other four countries' guidelines, and full adherence was observed in most children. When faced with vague or broad guidelines, healthcare workers may be required to lean on clinical judgement for decision-making on allocating limited resources such as oxygen.[22] More specific recommendations such as allocation to those at highest risk of clinical deterioration or based upon oxygen saturation or specific clinical signs

For peer review only

could help users prioritize when therapeutics are in short supply and drive higher guideline adherence.[23]

Severe pneumonia treatment recommendations contained more components and were less likely to be fully adhered to compared to non-severe pneumonia. Resource shortages, including oxygen and pulse oximetry, challenge the ability to adhere to recommendations.[24]

Diarrhea guideline adherence

Most children with diarrhea were treated with guideline non-adherent care. Fluid management had the lowest adherence, including non-provision of recommended ORS and IVF. Vitamin A and folate for persistent diarrhea and zinc for any form of diarrhea were also subject to low adherence. Other studies in low-resource settings also reported low adherence to fluid and zinc reccomendations,[25] This may also be driven by clinical practice culture, stock outs, lack of human resources, and low provider knowledge about zinc treatment for diarrhea.[26]

All guidelines considered in this analysis recommend that clinicians should not provide antibiotics for diarrhea in the absence of dysentery, cholera, or an indicated comorbidity. This recommendation was fully adhered to in most cases. However, for patients with dysentery, providers frequently prescribed a different antibiotic than that specified in guidelines. In contrast, a prior study in two Kenyan facilities reported frequent over-prescription of initial antibiotics in non-dysentery uncomplicated diarrhea and under-prescription in dysentery.[27] Our analysis documented and accounted for the presence of comorbidities indicative of initial antibiotic treatment, which is a novel addition to the literature. This might explain some of the over prescription of antibiotics in uncomplicated diarrhea observed in the literature, which typically do not take comorbidities into account.

For peer review only

In this study, children with diarrhea who presented with wasting at were less likely to receive guideline adherent care, compared to non-wasted children. This could result from over-cautiousness of providers in dehydration management among wasted children because of the potentially narrow safety range of fluids in severe wasting.[28] Additionally, children with diarrhea from poorer households were less likely to receive guideline adherent care. Further investigation is necessary to determine if out-of-pocket costs present a barrier to appropriate care. Within our population of children under 2 years old, older children were also less likely to receive adherent care compared to younger children. Evidence from other diseases (malaria and lower respiratory tract infections), similarly, found older children received less guideline adherent care.[29] Some guideline recommendations are different for younger vs older children, especially less than 6 months vs 6 months or older. For example, breast milk replaces ORS in cases requiring oral rehydration. The latter requires a hospital supply or purchase ability by the family which could lead to lower adherence in older children.

SM guideline adherence

Most patients with SM were treated with partially adherent care in this study. A Kenyan study investigating inpatient SM guideline adherence previously reported low adherence for hypoglycemia (15%), hypothermia (5%), and dehydration (31%) management.[30]

Strengths and limitations

This analysis was based on a strict interpretation of guidelines. If antibiotics for pneumonia or dysentery were prescribed based on local sensitivity patterns not described in guidelines, this was considered antibiotic non-adherence. This is true particularly in Pakistan where study physicians report that they purposefully avoid first line antibiotics due to high pathogen resistance prevalence.

For peer review only

We also acknowledge the potential influence of unmeasured confounders such as the clinical experience and expertise of different healthcare professionals who treated children within these hospitals in our study. Future research would benefit from systematically collecting such provider-level data to quantitatively explore these relationships and their impact on patient care outcomes.

It is possible that treatment was received without documentation as a prescription. For example, zinc or ORS could have been provided directly to caregivers without a prescription and therefore not recorded in the study database. Furthermore, it is possible that delayed documentation could have resulted in non-adherence overestimation. These issues will be explored in future qualitative research. Additionally, data from this study was collected in the context of a cohort study in hospitals with highly trained and monitored staff and with a patient population with a disproportionately high proportion of wasted children; the frequency of guideline non-adherence observed might not be generalizable to a routine clinical care setting as a result.

Importantly, adherence in this study is not a proxy for quality of care. Non-adherence to guidelines may not always mean that care was sub-optimal, rather decisions to deviate from guideline are often intentional and valid, driven by complex factors such as institutional norms, resource shortages, or clinical judgement. Measuring quality of care solely based upon adherence to guidelines may mask important nuances not currently captured by guidelines, or unclarity in the guidelines themselves.

CONCLUSION

Pediatric treatment guidelines aim to consolidate evidence-based recommendations into straightforward clinical tools, relevant across cadres and settings. This study examined guideline

For peer review only

adherence for pneumonia, diarrhea, and SM and relevant sub-conditions. We found instances of both unexplained and explained guideline non-adherence. These findings highlight opportunities to improve guidelines by increasing specificity for individual recommendations. For example, clarifying oxygen supplementation recommendations for severe pneumonia, or SM management in children under 6 months of age may help improve adherence and clinical outcomes. However, this study also highlights opportunity for strategic nuance, such as providing core guideline recommendations requiring high fidelity with required resource support in addition to ensuring that recommendations are tailored at the local level (e.g., use of antibiotics based on local sensitivity patterns, end-user perspectives, resources and cost considerations of treatment). This analysis also identified areas of complexity and potential confusion in guideline interpretation especially when a child has multiple conditions, underscoring the importance of addressing comorbidities in guidelines. To improve pediatric guideline adherence in low-resource settings, critical review of existing guidelines is necessary to ensure that they fully support clinicians in complex clinical scenarios.

For peer review only

CONTRIBUTORS

ARM, JLW, DMD, and KBT conceptualized the study. ARM, DMD, KBT, and MA contributed to the methodology. ARM and KBT were responsible for data curation. ARM and RAS accessed and verified the data underlying the study. RAS did the formal analysis and prepared the original draft of the manuscript. ARM, JLW, JAB, DMD, KBT, MA, RHB, EMu, WV, EMb, MJC, TA, AFS, MN, and AHD contributed to critically reviewing and editing the manuscript. All authors had full access to all the data in the study and accept responsibility to submit for publication.

509 DATA SHARING

510 Any reasonable requests to share data will be considered by the CHAIN consortium. Data requests
511 should be sent to the corresponding author.

512

513 DECLARATION OF INTERESTS

514 We declare no competing interests.

515

516 FUNDING

517 This secondary analysis of the CHAIN data was funded by the National Institutes of Health (NIH),
518 grant number 5R03HD099270-02. The primary CHAIN study was supported by the Bill &
519 Melinda Gates Foundation, grant number OPP1131320. For the purpose of Open Access, the
520 author has applied a CC-BY public copyright license to any author accepted manuscript version
521 arising from this submission.

522

523 ACKNOWLEDGEMENT

524 We thank the CHAIN Network for providing the data for this secondary analysis, and all CHAIN
525 participants, hospitals, and the CHAIN clinical and data management teams. We are grateful to the
526 CHAIN sites who responded to our requests for study site information. This work was supported,
527 in whole or in part, by the Bill & Melinda Gates Foundation [OPP131320]. Under the grant

For peer review only

528 conditions of the Foundation, a Creative Commons Attribution 4.0 Generic License has already
529 been assigned to the Author Accepted Manuscript version that might arise from this submission.

530

531 ## ETHICAL APPROVAL

532 The parent CHAIN cohort study protocol was approved by the Oxford Tropical Research Ethics
533 Committee, and the institutional review boards of all partner sites. This secondary analysis was
534 approved by the University of Washington Human Subjects Division (IRB ID #00006878).

535

536

For peer review only

REFERENCE

1 UNICEF. Under-five mortality. 2019.https://data.unicef.org/topic/child-survival/under-five-mortality/ (accessed 12 Dec 2021).

2 Black RE, Victora CG, Walker SP, *et al.* Maternal and child undernutrition and overweight in low-income and middle-income countries. *Lancet (London, England)* 2013;**382**:427–51. doi:10.1016/S0140-6736(13)60937-X

3 Hug L, Sharrow D, You D. Levels & Trends in Child Mortality. 2017. https://reliefweb.int/sites/reliefweb.int/files/resources/Levels-and-trends-in-child-mortality-IGME-English_2021.pdf

4 Kuruvilla S, Bustreo F, Kuo T, *et al.* The Global strategy for women's, children's and adolescents' health (2016-2030): a roadmap based on evidence and country experience. *Bull World Health Organ* 2016;**94**:398–400. doi:10.2471/BLT.16.170431

5 Hossain M, Chisti MJ, Hossain MI, *et al.* Efficacy of World Health Organization guideline in facility-based reduction of mortality in severely malnourished children from low and middle income countries: A systematic review and meta-analysis. *J Paediatr Child Health* 2017;**53**:474–9. doi:10.1111/jpc.13443

6 Turner EL, Nielsen KR, Jamal SM, *et al.* A Review of Pediatric Critical Care in Resource-Limited Settings: A Look at Past, Present, and Future Directions. *Front Pediatr* 2016;**4**:5. doi:10.3389/fped.2016.00005

7 Nolan T, Angos P, Cunha AJ, *et al.* Quality of hospital care for seriously ill children in less-developed countries. *Lancet (London, England)* 2001;**357**:106–10. doi:10.1016/S0140-6736(00)03542-X

For peer review only

8 English M, Ntoburi S, Wagai J, *et al.* An intervention to improve paediatric and newborn care in Kenyan district hospitals: Understanding the context. *Implement Sci* 2009;**4**:1–8. doi:10.1186/1748-5908-4-42

9 Solà I, Carrasco JM, Díaz Del Campo P, *et al.* Attitudes and perceptions about clinical guidelines: A qualitative study with spanish physicians. *PLoS One* 2014;**9**. doi:10.1371/journal.pone.0086065

10 Arts DL, Voncken AG, Medlock S, *et al.* Reasons for intentional guideline non-adherence: A systematic review. *Int J Med Inform* 2016;**89**:55–62. doi:10.1016/j.ijmedinf.2016.02.009

11 Hendaus MA, Alhammadi AH, Razig EA, *et al.* Pediatricians' perceptions of clinical practice guidelines. *J Multidiscip Healthc* 2014;**7**:349–54. doi:10.2147/JMDH.S66147

12 Osterholt DM, Onikpo F, Lama M, *et al.* Improving pneumonia case-management in Benin: a randomized trial of a multi-faceted intervention to support health worker adherence to Integrated Management of Childhood Illness guidelines. *Hum Resour Health* 2009;**7**:77. doi:10.1186/1478-4491-7-77

13 Blacklock C, Gonçalves Bradley DC, Mickan S, *et al.* Impact of Contextual Factors on the Effect of Interventions to Improve Health Worker Performance in Sub-Saharan Africa: Review of Randomised Clinical Trials. *PLoS One* 2016;**11**:e0145206.

14 Childhood Acute Illness and Nutrition Network. Childhood Acute Illness and Nutrition (CHAIN) Network: a protocol for a multi-site prospective cohort study to identify modifiable risk factors for mortality among acutely ill children in Africa and Asia. *BMJ Open* 2019;**9**:e028454. doi:10.1136/bmjopen-2018-028454

For peer review only

For peer review only - http://bmjopen.bmj.com/site/about/guidelines.xhtml

15 Tickell KD, Mangale DI, Tornberg-Belanger SN, *et al.* A mixed method multi-country assessment of barriers to implementing pediatric inpatient care guidelines. *PLoS One* 2019;**14**:1–17. doi:10.1371/journal.pone.0212395

16 Diallo AH, Sayeem Bin Shahid ASM, Khan AF, *et al.* Childhood mortality during and after acute illness in sub-Saharan Africa and South Asia - The CHAIN cohort study. *medRxiv* 2021;:2021.11.24.21266806. doi:10.1101/2021.11.24.21266806

17 Rutstein S. Steps to construct new DHS Wealth Index. 2015.https://dhsprogram.com/programming/wealth index/Steps_to_constructing_the_new_DHS_Wealth_Index.pdf

18 WHO. Integrated Management of newborn and Childhood Illness Module: 1. An Introduction to the Integrated Management of Newborn and Childhood Illness (IMNCI). OpenLearnCreate. 2020;:1–8.

19 Azur MJ, Stuart EA, Frangakis C, *et al.* Multiple imputation by chained equations: what is it and how does it work? *Int J Methods Psychiatr Res* 2011;**20**:40–9. doi:10.1002/mpr.329

20 Salih KEM, Bilal JA, Alfadeel MA, *et al.* Poor adherence to the World Health Organization guidelines of treatment of severe pneumonia in children at Khartoum , Sudan. 2014;:1–4.

21 Teixeira Rodrigues A, Roque F, Falcão A, *et al.* Understanding physician antibiotic prescribing behaviour: A systematic review of qualitative studies. *Int J Antimicrob Agents* 2013;**41**:203–12. doi:10.1016/j.ijantimicag.2012.09.003

22 Dauncey JW, Olupot-Olupot P, Maitland K. Healthcare-provider perceptions of barriers to oxygen therapy for paediatric patients in three government-funded eastern Ugandan

For peer review only

hospitals; A qualitative study. *BMC Health Serv Res* 2019;**19**:1–9. doi:10.1186/s12913-019-4129-7

23 Wen B, Brals D, Bourdon C, *et al.* Predicting the risk of mortality during hospitalization in sick severely malnourished children using daily evaluation of key clinical warning signs. *BMC Med* 2021;**19**:1–14. doi:10.1186/s12916-021-02074-6

24 Mvalo T. Need for routine pulse oximetry and oxygen delivery systems in low-income and middle-income countries. *Lancet Glob Heal* 2022;**10**:e301–2. doi:10.1016/S2214-109X(22)00025-0

25 Gathara D, English M, van Hensbroek MB, *et al.* Exploring sources of variability in adherence to guidelines across hospitals in low-income settings: A multi-level analysis of a cross-sectional survey of 22 hospitals. *Implement Sci* 2015;**10**:1–8. doi:10.1186/s13012-015-0245-x

26 Biswas D, Hossin R, Rahman M, *et al.* An ethnographic exploration of diarrheal disease management in public hospitals in Bangladesh: From problems to solutions. *Soc Sci Med* 2020;**260**:113185. doi:10.1016/j.socscimed.2020.113185

27 Rhee C, Aol G, Ouma A, *et al.* Inappropriate use of antibiotics for childhood diarrhea case management - Kenya, 2009-2016. *BMC Public Health* 2019;**19**:1–12. doi:10.1186/s12889-019-6771-8

28 Houston KA, Gibb JG, Maitland K. Intravenous rehydration of malnourished children with acute gastroenteritis and severe dehydration: A systematic review. *Wellcome open Res* 2017;**2**:65. doi:10.12688/wellcomeopenres.12346.1

29 De Bruycker M, Van den Bergh R, Dahmane A, *et al.* Non-adherence to standard

For peer review only

treatment guidelines in a rural paediatric hospital in Sierra Leone. *Public Heal Action* 2013;**3**:118–24. doi:10.5588/pha.12.0060

30 Warfa O, Njai D, Ahmed L, *et al.* Evaluating the level of adherence to Ministry of Health guidelines in the management of severe acute malnutrition at Garissa Provincial General Hospital, Garissa, Kenya. *Pan Afr Med J* 2014;**17**:214. doi:10.11604/pamj.2014.17.214.3821

For peer review only

SUPPLEMENTAL TABLES

For peer review only

Prevalence and correlates of pediatric guideline non-adherence across six low and middle-income countries

Supplemental Table 1: Comparison of country/institution/WHO guidelines for diagnosis of pneumonia, diarrhea and SM sub-conditions

Variables	Bangladesh	Burkina Faso	Kenya	Malawi	Pakistan	Uganda	WHO
Guideline used in site	Institutional guideline used for pneumonia[1], Diarrhea[2] and SM[3]	WHO guideline used for pneumonia and diarrhea<br><br>National guideline used for SM[4]	National guideline used for pneumonia, diarrhea,[5] and SM[6]	Institutional guidelines used for pneumonia,[7] diarrhea[8], and SM[9]	WHO guideline used for pneumonia<br><br>Institutional guideline used for diarrhea[10]<br><br>National guideline used for SM[11]	National guidelines used for pneumonia, diarrhea, and SM[12]	Pocket Book of Hospital Care for Children[13]
**Pneumonia**							
Severe Pneumonia	Cough or difficulty breathing PLUS at least one of the following:<br><br>-central cyanosis or oxygen saturation <90%<br><br>-severe respiratory distress (e.g. grunting, very severe chest indrawing)<br><br>-signs of pneumonia with a general danger sign (inability to breastfeed or drink, lethargy or unconscious,<br><br>convulsions)	Cough or difficulty breathing PLUS at least one of the following:<br><br>-central cyanosis or oxygen saturation <90%<br><br>-severe respiratory distress (e.g., grunting, very severe chest indrawing)<br><br>-signs of pneumonia with a general danger sign (inability to breastfeed or drink, lethargy or unconscious, convulsions)	Cough or difficulty breathing<br><br>PLUS, one of the danger signs:<br><br>-oxygen saturation <90%,<br><br>-cyanosis<br><br>-inability to drink/breastfeed-AVPU =V/P/U, -grunting	Cough or difficulty breathing PLUS<br><br>-central cyanosis /oxygen saturation <90%,<br><br>-severe respiratory distress (e.g., grunting, severe indrawing, head bobbing, nasal flaring), -convulsion/ loss of consciousness/ feeding/ lethargy	Cough or difficulty breathing PLUS at least one of the following:<br><br>-central cyanosis or oxygen saturation <90%<br><br>-severe respiratory distress (e.g., grunting, very severe chest indrawing)<br><br>-signs of pneumonia with a general danger sign (inability to breastfeed or drink, lethargy or unconscious,<br><br>convulsions)	Cough, fast breathing, AND at least one of the following-<br><br>-central cyanosis or<br><br>-oxygen saturation <90%<br><br>-inability to feed, vomiting everything*<br><br>-convulsions, lethargy, decreased level of consciousness<br><br>-severe respiratory distress (severe chest indrawing, grunting, nasal flaring)<br><br>-extrapulmonary features, e.g., confusion or disorientation	Cough or difficulty breathing PLUS at least one of the following:<br><br>-central cyanosis or oxygen saturation <90%<br><br>-severe respiratory distress (e.g., grunting, very severe chest indrawing)<br><br>-signs of pneumonia with a general danger sign (inability to breastfeed or drink, lethargy or unconscious, convulsions)

2

For peer review only - http://bmjopen.bmj.com/site/about/guidelines.xhtml

Prevalence and correlates of pediatric guideline non-adherence across six low and middle-income countries

Non-severe Pneumonia	Cough or difficult breathing plus at least one of the following:<br>-fast breathing<br>-lower chest wall indrawing	Cough or difficult breathing plus at least one of the following:<br>-fast breathing<br>-lower chest wall indrawing	Lower chest wall indrawing OR fast breathing	Fast breathing	Cough or difficult breathing plus at least one of the following:<br>-fast breathing<br>-lower chest wall indrawing	Cough, and fast breathing, and mild chest indrawing	Cough or difficult breathing plus at least one of the following:<br>-fast breathing<br>-lower chest wall indrawing
**Diarrhea**							
Acute watery diarrhea with severe dehydration	Acute watery diarrhea and signs of some dehydration PLUS any one of the following:<br>-lethargic/comatose<br>-unable to drink<br>-skin pinch goes back very slowly<br>-radial pulse uncountable or absent*	Diarrhea PLUS any two signs or symptoms of severe dehydration with diarrhea:<br>-lethargic, drowsy or unconscious<br>-sunken eyes<br>-sunken fontanelle<br>-drinks poorly or not able to drink<br>-skin pinch goes back very slowly<br>(>2 seconds)	Diarrhea PLUS unable to drink or AVPU<A, plus sunken eyes, or return of skin pinch >=2 secs	Diarrhea PLUS two or more of the following signs:<br>-lethargy/ unconsciousness<br>-sunken Eyes<br>-unable to drink/drinks poorly<br>-skin pinch goes back very slowly (≥ 2 seconds)	Diarrhea PLUS any two signs or symptoms of severe dehydration<br>-lethargic, drowsy or unconscious<br>-sunken eyes<br>-sunken fontanelle<br>-drinks poorly or not able to drink<br>-skin pinch goes back very slowly<br>(>2 seconds)	Diarrhea PLUS clinical features of severe dehydration<br>-lethargic, drowsy or unconscious<br>-sunken eyes<br>-sunken fontanelle<br>-drinks poorly or not able to drink<br>-skin pinch goes back very slowly<br>(>2 seconds)	Diarrhea PLUS any two signs or symptoms of severe dehydration<br>-lethargic, drowsy or unconscious<br>-sunken eyes<br>-sunken fontanelle<br>-drinks poorly or not able to drink<br>-skin pinch goes back very slowly<br>(>2 seconds)
Acute watery diarrhea with Some dehydration	Acute watery diarrhea PLUS at least two signs including one (*) sign<br>-irritable/less active*<br>-sunken eyes<br>-dry tongue<br>-thirsty (drinks eagerly)<br>-skin pinch goes back slowly*<br>-reduced radial pulse*	Diarrhea PLUS if the child has two or more of the following signs, he or she has some dehydration:<br>-restlessness or irritability<br>-thirsty and drinks eagerly<br>-sunken eyes<br>-skin pinch goes back slowly.<br>If a child has only one of the above	Diarrhea PLUS able to drink adequately but 2 or more of: Sunken eyes, return of skin pinch 1-2 secs, restlessness/ irritability	Diarrhea PLUS two or more of the following signs:<br>-restlessness/ irritability<br>-sunken eyes<br>-drinks eagerly/thirsty<br>-skin pinch goes back slowly	Diarrhea PLUS if the child has two or more of the following signs, he or she has some dehydration:<br>-restlessness or irritability<br>-thirsty and drinks eagerly<br>-sunken eyes<br>-skin pinch goes back slowly<br>If a child has only one of the above signs and one of the signs of	Diarrhea PLUS clinical features of some dehydration:<br>-restless, irritable<br>-sunken Eyes<br>-sunken fontanelle<br>- drinks eagerly, thirsty<br>-skin pinch goes back slowly; <2 seconds	Diarrhea PLUS if the child has two or more of the following signs, he or she has some dehydration:<br>-restlessness or irritability<br>-thirsty and drinks eagerly<br>-sunken eyes<br>-skin pinch goes back slowly.<br>If a child has only one of the above signs and one of the signs of<br>severe dehydration (e.g. restlessness or irritable and drinking poorly), then the<br>child also has some dehydration

For peer review only

For peer review only - http://bmjopen.bmj.com/site/about/guidelines.xhtml

		signs and one of the signs of<br><br>severe dehydration (e.g., restlessness or irritable and drinking poorly), then the<br><br>child also has some dehydration			severe dehydration (e.g., restlessness or irritable and drinking poorly), then the<br><br>child also has some dehydration		
Acute watery diarrhea with no dehydration	Acute watery diarrhea PLUS normal awareness condition, eyes, tongue, thirst, skin pinch, radial pulse	Diarrhea PLUS no sign of dehydration	Diarrhea PLUS fewer than 2 of the signs of dehydration	Diarrhea PLUS not enough signs to classify as some or severe dehydration	Diarrhea PLUS no sign of dehydration	Diarrhea PLUS clinical features of no dehydration:<br><br>-alert<br><br>-eyes not sunken<br><br>-fontanelle not sunken<br><br>-drinks normally<br><br>-skin pinch goes back immediately	Diarrhea PLUS no sign of dehydration
Persistent diarrhea	-	Diarrhea, with or without blood, that begins acutely and lasts for ≥ 14 days with no signs of dehydration	Diarrhea, with or without blood, that begins acutely and lasts for ≥ 14 days with no signs of dehydration	Diarrhea, with or without blood, that begins acutely and lasts for ≥ 14 days with no signs of dehydration	Diarrhea, with or without blood, that begins acutely and lasts for ≥ 14 days with no signs of dehydration	Diarrhea, with or without blood, that begins acutely and lasts for ≥ 14 days with no signs of dehydration	Diarrhea, with or without blood, that begins acutely and lasts for ≥ 14 days
Severe Persistent diarrhea	-	When there is some or severe dehydration, persistent diarrhea is classified as ‘severe’	When there is some or severe dehydration, persistent diarrhea is classified as ‘severe’	When there is some or severe dehydration, persistent diarrhea is classified as ‘severe’	When there is some or severe dehydration, persistent diarrhea is classified as ‘severe’	When there is some or severe dehydration, persistent diarrhea is classified as ‘severe’	When there is some or severe dehydration, persistent diarrhea is classified as ‘severe’
Dysentery	Diarrhea PLUS<br><br>-febrile≥ 38°C<br><br>-passes bloody or mucoid stools*<br><br>-has straining or tenesmus.	Blood mixed with the stools	Blood mixed with the stools	Frequent loose stools with visible red blood	Blood mixed with the stools	Bloody diarrhea, visible blood and mucus	Blood mixed with the stools

For peer review only - http://bmjopen.bmj.com/site/about/guidelines.xhtml

Prevalence and correlates of pediatric guideline non-adherence across six low and middle-income countries

**SM**							
SM	Bilateral oedema of both feet OR WFH/L <-3 z scores OR MUAC<125mm for children>=12 months<br><br>For <12 months, WLZ<-3 OR bipedal oedema	Bilateral pitting oedema OR severe wasting (WHZ< -3 z scores OR MUAC<115mm)<br><br>For <6 months, WLZ<-3 OR bipedal oedema OR visible wasting	Bilateral pitting oedema OR severe wasting (WHZ< -3 z scores OR MUAC<115mm)<br><br>For <6 months, WLZ<-3 OR bipedal oedema OR visible wasting	Bilateral pitting oedema OR severe wasting (WHZ< -3 z scores OR MUAC<115mm)<br><br>For <6 months, WLZ<-3 OR bipedal oedema	Bilateral oedema of both feet OR WFH/L <-3 z scores OR MUAC<115mm OR visible severe wasting<br><br>For <6 months, WLZ<-3 OR bipedal oedema OR visible wasting	Oedema of both feet OR WFH/L <-3 z scores OR MUAC<115mm OR visible severe wasting<br><br>For <6 months, WLZ<-3 OR bipedal oedema OR visible wasting	Bilateral oedema of both feet OR WFH/L <-3 z scores OR MUAC<115mm OR visible severe wasting<br><br>For <6 months, WLZ<-3 OR bipedal oedema OR visible wasting
Hypoglycemia in SM	Blood sugar <3 mmol/L	Blood sugar <3 mmol/L	Blood sugar <3 mmol/L	Blood sugar <3 mmol/L	Blood sugar <3 mmol/L	Blood sugar <3 mmol/L	Blood sugar <3 mmol/L
Hypothermia in SM	Axillary temperature <35c and rectal temperature <35·5c	Axillary temperature <35c and rectal temperature <35·5c	Axillary temperature <35c and rectal temperature <35·5c	Axillary temperature <35c	Axillary temperature <35c and rectal temperature <35·5c	Axillary temperature <35c and rectal temperature <35·5c	Axillary temperature <35c and rectal temperature <35·5c
Dehydration in SM	Signs of some or severe dehydration	Recent fluid loss, recent change in gaze, sunken eye, absence of superficial veins	All children with watery diarrhea or reduced urine output	Signs of some or severe dehydration	All children with watery diarrhea or reduced urine output	All children with watery diarrhea or reduced urine output	All children with watery diarrhea or reduced urine output
Severe Anemia in SM	Hb<5g/dl	Hb<4g/dl	Hb<4g/dl	Hb<4 OR Hb<6 with respiratory distress	Hb<4g/dl OR Hb 4-6g/dl with respiratory distress	Hb<4g/dl OR Hb 4-6g/dl with respiratory distress	Hb<4g/dl OR Hb 4-6g/dl with respiratory distress
Skin lesion in SM	Weeping skin lesions	Skin lesions in kwashiorkor	Dermatosis (patches of skin abnormally light or dark, shedding of skin, ulceration of skin and/or weeping<br><br>lesions)	No information	Hypo-or hyperpigmentation, Desquamation, Ulceration, Exudative lesions often with secondary infections, including Candida	Skin lesions in kwashiorkor	Hypo-or hyperpigmentation, Desquamation, Ulceration, Exudative lesions often with secondary infections, including Candida
Infection in SM	Most children with SM in in-patient care have infections and all are routinely treated	All children with SM should be treated for infection even in absence of clinical signs	Nearly all children with SM have bacterial infections. (all children should routinely receive antibiotic)	Typical signs of infections are absent. (so, antibiotics recommended in all children)	Most of the children with SM in in-patient care have infections and all are routinely treated	Assume children with SM have infection	Assume children with SM have infection
SM with measles	SM with measles	SM with measles	SM with measles	-	SM with measles	SM with measles	SM with measles

1. Dhaka Hospital. icddr,b Treatment Guidelines for Community Acquired Pneumonia. :1.

2. Nutrition & Clinical Services Division , icddr , b Treatment for Cholera and other Diarrheas.

For peer review only - http://bmjopen.bmj.com/site/about/guidelines.xhtml

Prevalence and correlates of pediatric guideline non-adherence across six low and middle-income countries

3. Ahmed T. Manual for Standardized Management of Severely Malnourished Children. 1–49 p.

4. Ministère de la Santé. Protocole National: Prise En Charge Integree De La Malnutrition Aiguë (Pcima), Burkina Faso. 2014;154.

5. Ministry of Health Kenya. Basic Paediatric Protocols. 2016.

6. Ministry of Health Kenya. National Guidelines for Integrated Management of Acute Malnutrition. 2009.

7. Queen Elizabeth Central Hospital. Electronic protocols for the management of common childhood illnesses in Malawi: Pneumonia. 2018.

8. Queen Elizabeth Central Hospital. Electronic protocols for the management of common childhood illnesses in Malawi: Diarrhea. 2018.

9. Voskuijl W. Queen Elizabeth Central Hospital. Electronic protocols for the management of common childhood illnesses in Malawi: Malnutrition. 2018;

10. Pakistan Pediatric Association. Diarrhea in Children. 2010. p. 1–14.

11. Ministry of Health Pakistan. National Guidelines for the management of acute malnutrition among children under five and pregnant and lactating women Ministry of Health. 2009.

12. Ministry of Health Uganda. Uganda Clinical Guidelines. 2016.

13. WHO. Pocket Book of Hospital Care for Children. World Health Organization; 2013.

* These variables were not available in the dataset. The coding was done with available variables only

Note: All of the diagnosis of conditions and sub-conditions were determined based on signs and symptoms and guideline criteria with the exception of two conditions: diarrhea, pneumonia, and one sub-condition: measles (in SM). These three diagnoses were determined based upon clinician diagnosis

For peer review only

For peer review only - http://bmjopen.bmj.com/site/about/guidelines.xhtml

Supplemental Table 2: Comparison of country/institution/WHO guidelines for management of pneumonia, diarrhea and SM sub-conditions

Variables	Bangladesh	Burkina Faso	Kenya	Malawi	Pakistan	Uganda	WHO
Guideline used in site	Institutional guideline used for pneumonia[1], Diarrhea[2] and SM[3]	WHO guideline used for pneumonia and diarrhea<br>National guideline used for SM[4]	National guideline used for pneumonia, diarrhea,[5] and SM[6]	Institutional guidelines used for pneumonia,[7] diarrhea[8], and SM[9]	WHO guideline used for pneumonia<br>Institutional guideline used for diarrhea[10]<br>National guideline used for SM[11]	National guidelines used for pneumonia, diarrhea, and SM[12]	Pocket Book of Hospital Care for Children[13]
**Pneumonia**							
Severe Pneumonia	Ampicillin + Gentamicin	Ampicillin (or Benzylpenicillin) and Gentamicin<br>Give oxygen if saturation <90%<br>If fever: paracetamol<br>If wheeze: bronchodilator	Penicillin and Gentamicin<br>Give oxygen<br>If wheeze: bronchodilators	X-pen + Gentamicin<br>Give oxygen<br>If fever: paracetamol<br>If wheeze: salbutamol	Ampicillin (or Benzylpenicillin) and Gentamicin<br>Give oxygen if saturation <90%<br>If fever: paracetamol<br>If wheeze: salbutamol	Ampicillin (or Benzylpenicillin) and Gentamicin<br>Give oxygen if saturation <90%<br>If wheeze: salbutamol<br>If fever-paracetamol<br>If convulsion: diazepam or phenobarbital	Ampicillin (or Benzylpenicillin) and Gentamicin<br>Give oxygen if saturation <90%<br>If fever: paracetamol<br>If wheeze: salbutamol
Non-severe Pneumonia	Amoxicillin	Amoxicillin	Amoxicillin<br>If wheeze: bronchodilators	Amoxicillin<br>If fever: paracetamol<br>If wheeze: salbutamol	Amoxicillin	Amoxicillin<br>If fever-paracetamol<br>If wheeze: salbutamol	Amoxicillin
**Diarrhea**							
Diarrhea with severe dehydration	IV fluid I/V solution containing sodium, potassium, chloride and bicarbonate (e.g. Ringer's Lactate/ cholera saline)<br>Zinc	IV Ringers lactate or normal saline<br>Zinc (once the child starts to improve sufficiently)	IV Ringers' lactate or NG rehydration<br>Zinc<br>Antimicrobials are not indicated unless there is	IV Ringers lactate or normal saline<br>Zinc (when severe dehydration is corrected)	IV Ringers lactate or normal saline<br>Zinc (once the child starts to improve sufficiently)	IV fluid or ORS by NG<br>Zinc	IV Ringers lactate or normal saline<br>Zinc (once the child starts to improve sufficiently)

For peer review only - http://bmjopen.bmj.com/site/about/guidelines.xhtml

Prevalence and correlates of pediatric guideline non-adherence across six low and middle-income countries

	Antibiotic only if necessary		dysentery or proven amoebiasis or giardiasis.			Avoid unnecessary antibiotic	
Diarrhea with some dehydration	ORS	ORS<br>After reassessment, if there is no dehydration, give zinc supplement	ORS, zinc<br>Antimicrobials are not indicated unless there is<br>dysentery or proven amoebiasis or giardiasis.	ORS, zinc	ORS<br>After reassessment, if there is no dehydration, give zinc supplement	ORS, zinc	ORS<br>After reassessment, if there is no dehydration, give zinc supplement
Diarrhea with no dehydration	ORS	ORS, zinc	ORS, zinc	ORS or clean water, zinc	ORS, zinc	ORS, zinc	ORS, zinc
Persistent diarrhea with no dehydration	-	Fluid, zinc, vitamin A, minerals	ORS, zinc	ORS, zinc, vitamin A, folate, multimineral	Fluid, zinc, vitamin A, minerals	ORS, zinc, vitamin A	Fluid, zinc, vitamin A, minerals
Persistent diarrhea with some or severe dehydration	-	Fluid, zinc, vitamin A, folate, multimineral	Fluid, zinc	Fluid, zinc, vitamin A, folate, multimineral	Fluid, zinc, vitamin A, folate, multimineral	Fluid, zinc, vitamin A	Fluid, zinc, vitamin A, folate, multimineral
Dysentery	Azithromycin if Shigellosis; Metronidazole if Amoebiasis<br>Continue Ampicillin, Ciprofloxacin or Ceftriaxone if indicated for other causes	Ciprofloxacin if antibiotic sensitivity<br>is unknown. If local antimicrobial sensitivity is known, follow<br>local guidelines.<br>ceftriaxone to severely ill children	Ciprofloxacin	Azithromycin or Ciprofloxacin	Ciprofloxacin or Cefixime	Ciprofloxacin	Ciprofloxacin if antibiotic sensitivity<br>is unknown. If local antimicrobial sensitivity is known, follow<br>local guidelines.<br>ceftriaxone to severely ill children
**SM**							
Hypoglycemia in SM	Oral or IV glucose	Feed or 10% glucose/ sucrose	All children with SM are at risk and should be given a feed or 10% glucose/ sucrose immediately on admission	If hypoglycemia, give a feed and oral or 10% IV glucose immediately on admission	Assume all children with SM are hypoglycemic. Give a feed or 10% glucose/ sucrose immediately on admission	All children with SM are at risk and should be given a feed or 10% glucose/ sucrose immediately on admission	All children with SM are at risk and should be given a feed or 10% glucose/ sucrose immediately on admission

For peer review only

Hypothermia in SM	Keep warm<br><br>Ensure feeding	Give a feed or 10% glucose/ sucrose, warm the child, give antibiotics	Keep warm	Warm the child	Warm the child, feed 2 hourly	Feed immediately as in hypoglycemia, warm the child, give antibiotics	Feed immediately as in hypoglycemia, warm the child, give antibiotics
Dehydration in SM	ORS/IV fluid	ReSoMal except in case of shock/ unconscious	ReSoMal except in case of shock/ unconscious	ReSoMal/ORS	ReSoMal except in case of shock/ unconscious	ReSoMal except in case of shock/ unconscious	ReSoMal except in case of shock/ unconscious
Severe Anemia in SM	Blood transfusion<br><br>or<br><br>packed blood cell*	Blood transfusion + furosemide	Blood transfusion	Blood transfusion	Blood transfusion + furosemide	Blood transfusion + furosemide	Blood transfusion + furosemide
Skin lesion in SM	Potassium permanganate solution*	No zinc	No zinc	Cloxacillin or Flucloxacillin if infected skin lesion	Zinc supplementation	Zinc supplementation + ointments	Zinc supplementation
Infection in SM *(applicable to all inpatients for prevention or management of infection)*	Broad spectrum antibiotic	Ceftriaxone OR Ceftriaxone + Gentamicin OR Ceftriaxone + Metronidazole	Penicillin OR Ampicillin PLUS Gentamicin	Benzylpenicillin PLUS Gentamicin	Penicillin OR Ampicillin PLUS Gentamicin	Benzylpenicillin OR Ampicillin PLUS Gentamicin	Penicillin OR Ampicillin PLUS Gentamicin
Micronutrient deficiency in SM *(applicable to all children with SM)*	Vit A, zinc, folate, multivitamin, potassium*, magnesium*	If clinical anemia, give folic acid	If child not on premixed food the give folic acid	No information	Give folic acid, multivitamin, zinc, copper to all children with SM	If child not on premixed food the give folic acid, multivitamin, zinc, copper	If child not on premixed food the give folic acid, multivitamin, zinc, copper
SM requiring nutritional stabilization *(applicable to all* children with SM*)*	Liquid diet such as milk suji	F-75,<br><br>(for children<6 months, F-75 OR diluted F-100 OR breastmilk)	F-75,<br><br>(for children<6 months, F-75 OR diluted F-100 OR breastmilk)	F-75, to all children	F-75,<br><br>(for children<6 months, F-75 OR diluted F-100 OR breastmilk)	F-75,<br><br>(for children<6 months, F-75 OR diluted F-100 OR breastmilk)	F-75,<br><br>(for children<6 months, F-75 OR diluted F-100 OR breastmilk)
SM with measles	Not mentioned	Vitamin A	Vitamin A	No information	Vitamin A	Vitamin A	Vitamin A

1. Dhaka Hospital. icddr,b Treatment Guidelines for Community Acquired Pneumonia. :1.
2. Nutrition & Clinical Services Division , icddr , b Treatment for Cholera and other Diarrheas.
3. Ahmed T. Manual for Standardized Management of Severely Malnourished Children. 1–49 p.
4. Ministère de la Santé. Protocole National: Prise En Charge Integree De La Malnutrition Aiguë (Pcima), Burkina Faso. 2014;154.
5. Ministry of Health Kenya. Basic Paediatric Protocols. 2016.

For peer review only - http://bmjopen.bmj.com/site/about/guidelines.xhtml

Prevalence and correlates of pediatric guideline non-adherence across six low and middle-income countries

6. Ministry of Health Kenya. National Guidelines for Integrated Management of Acute Malnutrition. 2009.

7. Queen Elizabeth Central Hospital. Electronic protocols for the management of common childhood illnesses in Malawi: Pneumonia. 2018.

8. Queen Elizabeth Central Hospital. Electronic protocols for the management of common childhood illnesses in Malawi: Diarrhea. 2018.

9. Voskuijl W. Queen Elizabeth Central Hospital. Electronic protocols for the management of common childhood illnesses in Malawi: Malnutrition. 2018;

10. Pakistan Pediatric Association. Diarrhea in Children. 2010. p. 1–14.

11. Ministry of Health Pakistan. National Guidelines for the management of acute malnutrition among children under five and pregnant and lactating women Ministry of Health. 2009.

12. Ministry of Health Uganda. Uganda Clinical Guidelines. 2016.

13. WHO. Pocket Book of Hospital Care for Children. World Health Organization; 2013.

* These variables were not available in the dataset. The coding was done with available variables only

For peer review only

Prevalence and correlates of pediatric guideline non-adherence across six low and middle-income countries

Supplemental Table 3: Overlap between pneumonia, diarrhea, SM and other clinical diagnoses

Overlapping diagnoses	Conditions n (%)		
	Pneumonia (N=1095)	Diarrhea (N=1014)	SM (N=1096)
Pneumonia	-	418 (32)	384 (35)
Diarrhea	418 (38)	-	524 (48)
SM	384 (35)	524 (41)	-
Bronchiolitis	77 (7)	14 (1)	21 (2)
Upper respiratory tract infection	11 (1)	59 (5)	58 (5)
Tuberculosis	18 (2)	4 (0·3)	30 (3)
Otitis media	7 (0·6)	4 (0·3)	7 (0·6)
Asthma	4 (0·4)	-	1 (0·1)
Anemia	203 (19)	179 (14)	211 (19)
Sickle cell disease	5 (0·5)	1 (0·1)	4 (0·4)
Thalassemia	1 (0·1)	1 (0·1)	1 (0·1)
Renal impairment	-	1 (0·1)	3 (0·3)
Nephrotic syndrome	-	-	2 (0·2)
Liver disease	-	-	1 (0·1)
Ileus	2 (0·2)	2 (0·2)	1 (0·1)
Congenital cardiac disease	1 (0·1)	-	1 (0·1)
Sepsis	91 (8)	138 (11)	165 (15)
Malaria	108 (10)	96 (7)	137 (13)
Soft tissue infection	2 (0·2)	2 (0·2)	4 (0·4)
Urinary tract infection	3 (0·3)	8 (0·7)	3 (0·3)
Measles	48 (4)	13 (1)	17 (2)
Osteomyelitis	-	-	1 (0·1)
Febrile illness	1 (0·1)	21 (2)	4 (0·4)
Enteric fever	10 (0·9)	6 (0·7)	28 (3)
Epilepsy	1 (0·1)	-	-
Meningitis	32 (3)	14 (1)	25 (2)
Encephalopathy	2 (0·2)	4 (0·3)	2 (0·2)
Hydrocephalous	2 (0·2)	-	2 (0·2)
Developmental delay	2 (0·2)	3 (0·2)	10 (1)
Cerebral Palsy	4 (0·4)	2 (0·2)	5 (0·5)
Note: The denominator is all cases of the syndrome in column for all proportions			

For peer review only

Supplemental Table 4: Guideline adherence for conditions and sub-conditions, by country

Conditions and sub-conditions	Bangladesh<br>n<br>%			Burkina Faso<br>n<br>%			Kenya<br>n<br>%			Malawi<br>n<br>%			Pakistan<br>n<br>%			Uganda<br>n<br>%			Overall<br>n<br>%		
	Full	Partial	None	Full	Partial	None	Full	Partial	None	Full	Partial	None	Full	Partial	None	Full	Partial	None	Full	Partial	None
**PNEUMONIA[1]**	208<br>*75*	-	71<br>*25*	36<br>*36*	50<br>*50*	15<br>*15*	40<br>*21*	147<br>*78*	1<br>*0·5*	13<br>*18*	61<br>*82*	-	17<br>*9*	157<br>*82*	18<br>*9*	101<br>*71*	42<br>*29*	-	415<br>*42*	457<br>*47*	105<br>*11*
Non-severe	101<br>*73*	-	37<br>*27*	8<br>*35*	-	15<br>*65*	4<br>*11*	33<br>*87*	1<br>*3*	10<br>*37*	17<br>*63*	-	17<br>*50*	-	17<br>*50*	14<br>*64*	8<br>*36*	-	154<br>*54*	58<br>*21*	70<br>*24*
Severe	107<br>*76*	-	34<br>*24*	28<br>*36*	50<br>*64*	-	36<br>*24*	114<br>*76*	-	3<br>*6*	44<br>*94*	-	-	158<br>*100*	1<br>*1*	87<br>*72*	34<br>*28*	-	261<br>*38*	400<br>*57*	35<br>*5*
**DIARRHEA[2]**	259<br>*66*	132<br>*33*	4<br>*1*	36<br>*51*	9<br>*13*	26<br>*37*	2<br>*2*	49<br>*46*	56<br>*52*	16<br>*27*	24<br>*40*	20<br>*33*	6<br>*12*	18<br>*37*	25<br>*51*	4<br>*5*	23<br>*30*	49<br>*64*	323<br>*32*	255<br>*25*	435<br>*43*
AWD w/ severe dehydration	6<br>*19*	22<br>*71*	3<br>*10*	12<br>*57*	3<br>*14*	6<br>*29*	-	2<br>*100*	-	-	6<br>*60*	4<br>*40*	3<br>*27*	5<br>*46*	3<br>*27*	3<br>*16*	6<br>*32*	10<br>*53*	24<br>*26*	44<br>*47*	26<br>*28*
AWD w/ some dehydration	35<br>*70*	15<br>*30*	-	3<br>*60*	-	2<br>*40*	-	25<br>*54*	21<br>*46*	1<br>*20*	2<br>*40*	2<br>*40*	1<br>*10*	2<br>*20*	7<br>*70*	1<br>*7*	7<br>*50*	6<br>*43*	41<br>*33*	51<br>*39*	38<br>*29*
AWD w/ no dehydration	218<br>*69*	95<br>*30*	1<br>*0·3*	21<br>*47*	6<br>*13*	18<br>*40*	-	21<br>*39*	33<br>*61*	15<br>*38*	13<br>*33*	12<br>*30*	2<br>*13*	2<br>*13*	12<br>*75*	-	9<br>*26*	25<br>*74*	256<br>*51*	146<br>*29*	101<br>*20*
Persistent				-	-	-	2<br>*50*	-	2<br>*50*	-	2<br>*67*	1<br>*33*	-	4<br>*80*	1<br>*20*	-	-	6<br>*100*	2<br>*11*	6<br>*33*	10<br>*56*
Severe persistent				-	-	-	-	-	-	-	1<br>*50*	1<br>*50*	-	3<br>*75*	1<br>*25*	-	-	2<br>*100*	-	4<br>*50*	4<br>*50*
Dysentery	-	-	1<br>*100*	-	-	-	-	1<br>*100*	-	-	-	-	-	2<br>*67*	1<br>*33*	-	1<br>*100*	-	-	4<br>*67*	2<br>*33*
**SM[3]**	136<br>51	133<br>*49*	-	14<br>*8*	167<br>*92*	-	80<br>*37*	138<br>*63*	-	49<br>*34*	34<br>*41*	-	-	118<br>*100*	-	23<br>10	204<br>90	-	302<br>*28*	794<br>*72*	-
W/ hypoglycemia[4]	-	2<br>*100*	-	181<br>*100*	-	-	90<br>*41*	114<br>*52*	14<br>*6*	-	-	-	-	24<br>*20*	94<br>*80*	81<br>*36*	134<br>*59*	12<br>*5*	352<br>*47*	274<br>*37*	120<br>*16*
W/ hypothermia	-	-	-	-	-	-	1<br>*25*	-	3<br>*75*	1<br>*50*	-	1<br>*50*	-	-	-	-	4<br>*100*	-	2<br>*20*	4<br>40	4<br>40

For peer review only

Prevalence and correlates of pediatric guideline non-adherence across six low and middle-income countries

SM with dehydration	71<br>*93*	-	5<br>*7*	16<br>*94*	-	1<br>*6*	10<br>*50*	-	10<br>*50*	35<br>*81*	-	8<br>*19*	8<br>*50*	-	8<br>*50*	1<br>50	-	1<br>*50*	141<br>*81*		33<br>*19*
SM with severe anemia	-	-	-	21<br>*78*	-	6<br>*22*	3<br>*100*	-	-	-	-	-	1<br>*100*	-	-	-	-	-	25<br>*81*		6<br>*19*
SM with active measles				-	-	-	-	-	-				2<br>*25*	-	6<br>*75*	1<br>*14*	-	6<br>*86*	3<br>*20*		12<br>*80*
W/ micronutrient deficiency[5]	143<br>*53*	81<br>*30*	45<br>*17*	1<br>*0·9*	-	113<br>*99*	114<br>*52*	-	104<br>*48*				22<br>*19*	6<br>*5*	90<br>*76*	72<br>*32*	-	155<br>*68*	352<br>*37*	87<br>*9*	507<br>*54*
W/ infection[5]	238<br>*88*	-	31<br>*12*	41<br>*23*	48<br>*27*	92<br>*51*	161<br>*74*	36<br>*16*	21<br>*10*	62<br>*75*	-	21<br>*25*	2<br>*2*	1<br>*1*	115<br>*97*	206<br>*91*	7<br>*3*	14<br>*6*	710<br>*65*	92<br>*8*	294<br>*27*
Nutritional stabilization[5]	262<br>*97*	1<br>*0·4*	6<br>*2*	153<br>*85*	-	28<br>*16*	184<br>*84*	-	34<br>*16*	62<br>*75*	-	21<br>*25*	58<br>*49*	-	60<br>*51*	147<br>*65*	-	80<br>*35*	866<br>*79*	1<br>*0·1*	229<br>*21*
W/ skin lesion	[6]	[6]	[6]										8<br>*16*	-	42<br>*84*	-	-	109<br>*100*	8<br>*5*	-	151<br>*95*

[1] For pneumonia, comorbid SM was excluded as per Kenyan pneumonia treatment guidelines
[2] For diarrhea w/dehydration, comorbid SM was excluded, per guidelines
[3] Total SM cases in any adherence category may not be equal to the summation of all SM sub-conditions in that category since these are not mutually exclusive.
[4] Hypoglycemia treatment applies to all children with SM in Uganda, Kenya, and Pakistan as per guidelines irrespective of blood glucose level.
[5] Per guidelines, all children with SM should be treated for Infection and micronutrient deficiency and all children require nutritional stabilization. Micronutrients include Vit A, Zinc, Multivitamin, & Folic acid if therapeutic feed not given
[6] Variable for skin lesion treatment in Bangladesh not available in the data

Notes: (1) The denominator for all percentages is the total number of children with a specific condition, (2) AWD= Acute Watery Diarrhea, (3) A "-" indicates that there were no cases in the sample with the diagnosis (4) a blank cell indicates there are no relevant recommendation for a condition in the country, (5) In case of a tie, comparatively higher adherence level between the tie categories is assumed to be the majority

For peer review only

Prevalence and correlates of pediatric guideline non-adherence across six low and middle-income countries

Supplemental Table 5: Guideline adherence for specific recommendations, by sub-conditions

Recommendations	Bangladesh<br>n<br>%		Burkina Faso<br>n<br>%		Kenya<br>n<br>%		Malawi<br>n<br>%		Pakistan<br>n<br>%		Uganda<br>n<br>%		Overall<br>n<br>%	
	Full adherence	Not adherent	Full adherence	Not adherent	Full adherence	Not adherent	Full adherence	Not adherent	Full adherence	Not adherent	Full adherence	Not adherent	Full adherence	Not adherent
**PNEUMONIA[1]**														
Antibiotics for severe pneumonia	107<br>*76*	34<br>*24*	36<br>*46*	42<br>*54*	102<br>*68*	48<br>*32*	42<br>*89*	5<br>*11*	-	158<br>*100*	98<br>*81*	23<br>*19*	329<br>*52*	303<br>*48*
Antibiotics for non-severe pneumonia	101<br>*73*	37<br>*27*	8<br>*35*	15<br>*65*	5<br>*13*	33<br>*87*	12<br>*44*	15<br>*56*	17<br>*50*	17<br>*50*	16<br>*73*	6<br>*27*	129<br>*54*	112<br>*46*
Oxygen for severe pneumonia			78<br>*100* [3]	-	71<br>*47* [4]	79<br>*53* [4]	6<br>*13* [4]	41<br>*87* [4]	158<br>*100* [3]	-	121<br>*100* [3]	-	434<br>*78*	120<br>*22*
Paracetamol for fever (Severe Pneumonia)			74<br>*95*	4<br>*5*			47<br>*100*	-	156<br>*99*	2<br>*1*	119<br>*98*	2<br>*2*	396<br>*98*	8<br>*2*
Paracetamol for fever (non-severe Pneumonia)							27<br>*100*	-			22<br>*100*	-	49<br>*100*	-
Bronchodilator for wheeze (severe pneumonia)			67<br>*86*	11<br>*14*	134<br>*89*	16<br>*11*	39<br>*83*	8<br>*17*	128<br>*81*	30<br>*19*	109<br>*90*	12<br>*10*	477<br>*86*	77<br>*14*
Bronchodilator for wheeze (non-severe pneumonia)					36<br>*95*	2<br>*5*	20<br>*74*	7<br>*26*			20<br>*91*	2<br>*9*	76<br>*87*	11<br>*13*
**DIARRHEA[2]**														
Rehydration for AWD with severe dehydration	9<br>*29*	22<br>*71*	14<br>*67*	7<br>*33*	2<br>*100*	-	3<br>*30*	7<br>*70*	8<br>*73*	3<br>*27*	9<br>*47*	10<br>*53*	45<br>*48*	49<br>*52*
Zinc for AWD with severe dehydration	22<br>*71*	9<br>*29*	13<br>*62*	8<br>*38*	1<br>*50*	1<br>*50*	4<br>*40*	6<br>*60*	3<br>*27*	8<br>*73*	3<br>*16*	16<br>*84*	46<br>*49*	47<br>*51*
Rehydration for AWD with some dehydration	49<br>*98*	1<br>*2*	3<br>*60*	2<br>*40*	17<br>*37*	29<br>*63*	3<br>*60*	2<br>*40*	1<br>*10*	9<br>*90*	8<br>*57*	6<br>*43*	81<br>*80*	20<br>*20*
Zinc for AWD with some dehydration			3<br>*60*	2<br>*40*	22<br>*48*	24<br>*52*	1<br>*20*	4<br>*80*	3<br>*30*	7<br>*70*	1<br>*7*	13<br>*93*	30<br>*28*	76<br>*72*

For peer review only

Prevalence and correlates of pediatric guideline non-adherence across six low and middle-income countries

Rehydration for AWD with no dehydration	300	14	23	22	15	39	25	15	4	12	8	26	375	89
	96	*4*	*51*	*49*	*28*	*72*	*63*	*38*	*25*	*75*	*24*	*77*	*81*	*19*
No antibiotic in AWD	291	104	70	1	82	20	43	12	33	4	44	23	651	154
	74	*26*	*99*	*1*	*80*	*20*	*78*	*22*	*89*	*11*	*66*	*34*	*81*	*20*
Zinc for AWD with no dehydration			26	19	14	40	21	19	2	14	3	31	66	397
			58	*42*	*26*	*74*	*53*	*47*	*12*	*88*	*9*	*91*	*14*	*86*
Rehydration for persistent diarrhea			-	-	2	2	2	1	3	2	-	6	7	9
					50	*50*	*67*	*33*	*60*	*40*		*100*	*44*	*56*
Zinc for persistent diarrhea			-	-	2	2	1	2	1	4	-	6	4	12
					50	*50*	*33*	*67*	*20*	*80*		*100*	*25*	*75*
Vitamin for persistent diarrhea[5]			-	-			-	3			-	6	-	9
								100				*100*		*100*
Folate for persistent diarrhea			-	-			-	3					-	3
								100						*100*
Microminerals for persistent diarrhea			-	-			-	3					-	3
								100						*100*
Rehydration for severe persistent diarrhea			-	-			1	1	3	1	-	2	4	4
							50	*50*	*75*	*25*		*100*	*50*	*50*
Zinc for severe persistent diarrhea			-	-			-	2	-	4	-	2	-	8
								100		*100*		*100*		*100*
Vitamin for severe persistent diarrhea[5]			-	-			-	2			-	2	-	4
								100				*100*		*100*
Folate for severe persistent diarrhea			-	-			-	2					-	2
								100						*100*
Microminerals for severe persistent diarrhea			-	-			-	2					-	2
								100						*100*
Rehydration for dysentery			-	-	-	1	-	-	2	1	-	1	2	4
						100			*67*	*33*		*100*	*33*	*67*
Zinc for dysentery			-	-	-	1	-	-	1	2	-	1	1	5
						100			*33*	*67*		*100*	*17*	*83*
Antibiotics for dysentery	-	1	-	-	1	-	-	-	-	3	1	-	2	4
		100			*100*					*100*	*100*		*33*	*67*
**SM**														
Antibiotics in SM	236	33	41	140	161	57	62	21	2	116	206	21	710	386
	88	*12*	*23*	*77*	*74*	*26*	*75*	*25*	*2*	*98*	*91*	*9*	*65*	*35*
Fluid for dehydration and SM	71	5	16	1	10	10	35	8	8	8	1	1	141	33
	93	*7*	*94*	*6*	*50*	*50*	*81*	*19*	*50*	*50*	*50*	*50*	*81*	*19*

For peer review only - http://bmjopen.bmj.com/site/about/guidelines.xhtml

Prevalence and correlates of pediatric guideline non-adherence across six low and middle-income countries

Blood transfusion in SM with severe anemia	-	-	21	6	3	-	-	-	1	-	-	-	25	6
			78	*22*	*100*				*100*				*81*	*19*
Vitamin A in SM with measles			-	-	-	-			2	6	1	6	3	12
									25	*75*	*14*	*86*	*20*	*80*
Micronutrients in SM	143	126	68	113	114	104			22	96	72	155	467	629
	53	*47*	*38*	*62*	*52*	*48*			*19*	*81*	*32*	*68*	*43*	*57*
Fortified food in SM	262	7	153	28	184	34	62	21	58	60	147	80	866	230
	97	*3*	*85*	*16*	*84*	*16*	*74*	*25*	*49*	*51*	*65*	*35*	*79*	*21*
Zinc for skin lesion in SM									8	42	-	109	8	151
									16	*84*		*100*	*5*	*95*

[1] For pneumonia, comorbid SM was excluded as per Kenyan pneumonia treatment guidelines
[2] For diarrhea, SM cases are excluded from all countries
[3] Oxygen is recommended for severe pneumonia cases with SpO2<90% only
[4] Oxygen is recommended for all severe pneumonia cases
[5] Vitamins include vitamin A, folate, both, and/or multivitamin
Notes: (1) The denominator for all percentages is the total number of children with a specific condition, (2) AWD= Acute Watery Diarrhea, (3) A "-" indicates that there were no cases in the sample with the diagnosis (4) a blank cell indicates there are no relevant recommendation for a condition in the country

For peer review only

For peer review only - http://bmjopen.bmj.com/site/about/guidelines.xhtml

Supplemental Table 6: Adherence to pneumonia and associated sub-conditions, excluding children with SM (sensitivity analysis)

Pneumonia sub-conditions and specific recommendations	Total N	Full adherence n (%)	Partial adherence n (%)	No adherence n (%)
**Pneumonia**	659	187 (28)	385 (58)	87 (13)
**Non-severe Pneumonia**	169	54 (32)	56 (33)	59 (34)
Antibiotics	169	57 (34)		112 (66)
Paracetamol if fever	33	33 (100)		
Bronchodilator if wheeze	71	62 (87)		9 (13)
**Severe Pneumonia**	490	133 (27)	329 (67)	28 (6)
Antibiotics	490	242 (49)		248 (51)
Oxygen	430[1]	320 (74)		110 (26)
Paracetamol if fever	280	274 (98)		6 (2)
Bronchodilator if wheeze	430	372 (87)		58 (13)

[1] Oxygen is only given when low saturation in sever pneumonia in some countries

For peer review only

Prevalence and correlates of pediatric guideline non-adherence across six low and middle-income countries

Supplemental Table 7: Overall guideline adherence for conditions and sub-conditions without comorbidities

Conditions and sub-conditions	Overall n %		
	Full	Partial	None
**PNEUMONIA**	51 (27)	120 (64)	17 (9)
Non-severe	8 (21)	18 (47)	12 (32)
Severe	43 (29)	102 (68)	5 (3)
**DIARRHEA**	111 (37)	148 (49)	45 (15)
AWD w/ severe dehydration	1 (5)	14 (64)	7 (32)
AWD w/ some dehydration	4 (11)	28 (74)	6 (16)
AWD w/ no dehydration	106 (47)	105 (46)	16 (7)
Persistent	-	1 (33)	2 (67)
Severe persistent	-	-	1 (100)
Dysentery	-	4 (67)	2 (33)
**SM**	22 (22)	78 (78)	
W/ hypoglycemia	2 (67)	1 (33)	-
W/ hypothermia	-	3 (100)	-
SM with dehydration	2 (33)		4 (67)
SM with severe anemia	-	-	-
SM with active measles	-	-	-
W/ micronutrient deficiency	54 (54)	-	46 (46)
W/ infection	75 (75)	9 (9)	16 (16)
SM requiring nutritional stabilization	76 (76)	-	24 (24)
W/ skin lesion	1 (3)	-	34 (97)

Notes: (1) The denominator for all percentages is the total number of children with a specific condition without comorbidities, (2) AWD= Acute Watery Diarrhea, (3) A "-" indicates that there were no cases in the sample with the diagnosis and without comorbidities

For peer review only

STROBE Statement—Checklist of items that should be included in reports of ***cross-sectional studies***

	Item No	Recommendation	Page No
**Title and abstract**	1	(*a*) Indicate the study's design with a commonly used term in the title or the abstract	1
		(*b*) Provide in the abstract an informative and balanced summary of what was done and what was found	6
**Introduction**			
Background/rationale	2	Explain the scientific background and rationale for the investigation being reported	9-10
Objectives	3	State specific objectives, including any prespecified hypotheses	9
**Methods**			
Study design	4	Present key elements of study design early in the paper	10
Setting	5	Describe the setting, locations, and relevant dates, including periods of recruitment, exposure, follow-up, and data collection	10
Participants	6	(*a*) Give the eligibility criteria, and the sources and methods of selection of participants	10
Variables	7	Clearly define all outcomes, exposures, predictors, potential confounders, and effect modifiers. Give diagnostic criteria, if applicable	11-12
Data sources/ measurement	8*	For each variable of interest, give sources of data and details of methods of assessment (measurement). Describe comparability of assessment methods if there is more than one group	11-12
Bias	9	Describe any efforts to address potential sources of bias	13
Study size	10	Explain how the study size was arrived at	15
Quantitative variables	11	Explain how quantitative variables were handled in the analyses. If applicable, describe which groupings were chosen and why	13
Statistical methods	12	(*a*) Describe all statistical methods, including those used to control for confounding	13
		(*b*) Describe any methods used to examine subgroups and interactions	14
		(*c*) Explain how missing data were addressed	13
		(*d*) If applicable, describe analytical methods taking account of sampling strategy	N/A
		(*e*) Describe any sensitivity analyses	14
**Results**			
Participants	13*	(a) Report numbers of individuals at each stage of study—eg numbers potentially eligible, examined for eligibility, confirmed eligible, included in the study, completing follow-up, and analysed	15
		(b) Give reasons for non-participation at each stage	N/A
		(c) Consider use of a flow diagram	N/A
Descriptive data	14*	(a) Give characteristics of study participants (eg demographic, clinical, social) and information on exposures and potential confounders	15
		(b) Indicate number of participants with missing data for each variable of interest	13
Outcome data	15*	Report numbers of outcome events or summary measures	15-20

For peer review only - http://bmjopen.bmj.com/site/about/guidelines.xhtml

Main results	16	(*a*) Give unadjusted estimates and, if applicable, confounder-adjusted estimates and their precision (eg, 95% confidence interval). Make clear which confounders were adjusted for and why they were included	22-23
		(*b*) Report category boundaries when continuous variables were categorized	N/A
		(*c*) If relevant, consider translating estimates of relative risk into absolute risk for a meaningful time period	N/A
Other analyses	17	Report other analyses done—eg analyses of subgroups and interactions, and sensitivity analyses	19
**Discussion**			
Key results	18	Summarise key results with reference to study objectives	24
Limitations	19	Discuss limitations of the study, taking into account sources of potential bias or imprecision. Discuss both direction and magnitude of any potential bias	27
Interpretation	20	Give a cautious overall interpretation of results considering objectives, limitations, multiplicity of analyses, results from similar studies, and other relevant evidence	23-27
Generalisability	21	Discuss the generalisability (external validity) of the study results	26
**Other information**			
Funding	22	Give the source of funding and the role of the funders for the present study and, if applicable, for the original study on which the present article is based	29

For peer review only

BMJ Open

Prevalence and correlates of pediatric guideline non-adherence for initial empiric care in six low and middle-income settings: a hospital based cross sectional study

Journal:	*BMJ Open*
Manuscript ID	bmjopen-2023-078404.R2
Article Type:	Original research
Date Submitted by the Author:	22-Jan-2024
Complete List of Authors:	Shawon , R.A; University of Washington,<br>Denno, Donna; University of Washington<br>Tickell, Kirkby; University of Washington, Global Health; Childhood Acute Illness and Nutrition Network,<br>Atuhairwe, Michael; Makerere University<br>Bandsma, Robert; Centre for Global Child Health<br>Mupere, Ezekiel ; Makerere University, Paediatrics and Child Health<br>Voskuijl, Wieger ; Amsterdam University Medical Centres, Pediatrics<br>Mbale, Emmie; University of Malawi, Department of Pediatrics<br>Ahmed, Tahmeed; International Centre for Diarrhoeal Disease Research Bangladesh<br>Chisti, Md.; International Centre for Diarrhoeal Disease Research Bangladesh<br>Saleem, Ali; Aga Khan University Hospital<br>Ngari, Moses; Clinical Trials Facility<br>DIALLO, Hama; Universite Joseph Ki-Zerbo<br>Berkley, James ; KEMRI/Wellcome Trust Research Programme, Clinical Research<br>Walson, Judd; University of Washington<br>Means, Arianna; University of Washington
**Primary Subject Heading**:	Evidence based practice
Secondary Subject Heading:	Global health, Paediatrics, Public health, Infectious diseases
Keywords:	PAEDIATRICS, INFECTIOUS DISEASES, PUBLIC HEALTH

SCHOLARONE™
Manuscripts

I, the Submitting Author has the right to grant and does grant on behalf of all authors of the Work (as defined in the below author licence), an exclusive licence and/or a non-exclusive licence for contributions from authors who are: i) UK Crown employees; ii) where BMJ has agreed a CC-BY licence shall apply, and/or iii) in accordance with the terms applicable for US Federal Government officers or employees acting as part of their official duties; on a worldwide, perpetual, irrevocable, royalty-free basis to BMJ Publishing Group Ltd ("BMJ") its licensees and where the relevant Journal is co-owned by BMJ to the co-owners of the Journal, to publish the Work in this journal and any other BMJ products and to exploit all rights, as set out in our licence.

The Submitting Author accepts and understands that any supply made under these terms is made by BMJ to the Submitting Author unless you are acting as an employee on behalf of your employer or a postgraduate student of an affiliated institution which is paying any applicable article publishing charge ("APC") for Open Access articles. Where the Submitting Author wishes to make the Work available on an Open Access basis (and intends to pay the relevant APC), the terms of reuse of such Open Access shall be governed by a Creative Commons licence – details of these licences and which Creative Commons licence will apply to this Work are set out in our licence referred to above.

Other than as permitted in any relevant BMJ Author's Self Archiving Policies, I confirm this Work has not been accepted for publication elsewhere, is not being considered for publication elsewhere and does not duplicate material already published. I confirm all authors consent to publication of this Work and authorise the granting of this licence.

For peer review only

TITLE

Prevalence and correlates of pediatric guideline non-adherence for initial empiric care in six low and middle-income settings: a hospital based cross sectional study

AUTHORS

Riffat Ara Shawon, MPH[1,2]
Donna M. Denno, MD[1,3,4]
Kirkby D. Tickell, PhD[1,2,4]
Michael Atuhairwe, MBChB [4,8]
Robert H J Bandsma, PhD[4,13]
Ezekiel Mupere, PhD[4,8,9]
Wieger Voskuijl, PhD[4,10,11,12]
Emmie Mbale, MBBS[4, 14]
Tahmeed Ahmed, PhD[15]
Md. Jobayer Chisti, PhD[15]
Ali Faisal Saleem, MBBS[16]
Moses Ngari, PhD[5]
Abdoulaye Hama Diallo, PhD[17]
James A. Berkley, FRCPCH[4,5,6]
Judd L. Walson, MD[1,2,3,4,7]
Arianna Rubin Means, PhD[1]

CORRESPONDING AUTHOR INFORMATION

Name of the Corresponding Author: Riffat Ara Shawon
Affiliations:
1. Department of Global Health, University of Washington, Seattle, WA, USA.
2. Department of Epidemiology, University of Washington, Seattle, WA, USA.
Email Address: riasha47@gmail.com; shawon@uw.edu

For peer review only

AUTHOR AFFILIATIONS:

1. Department of Global Health, University of Washington, Seattle, WA, USA.
2. Department of Epidemiology, University of Washington, Seattle, WA, USA.
3. Departments of Pediatrics, University of Washington, Seattle, WA, USA.
4. The Childhood Acute Illness & Nutrition (CHAIN) Network, Nairobi, Kenya.
5. KEMRI/Wellcome Trust Research Programme, Kilifi, Kenya.
6. Centre for Tropical Medicine & Global Health, Nuffield Department of Medicine, University of Oxford, Oxford, UK.
7. Department of Medicine, University of Washington, Seattle, WA, USA.
8. Uganda-Case Western Reserve University Research Collaboration, Makerere University, Kampala, Uganda.
9. Department of Paediatrics and Child Health School of Medicine College of Health Sciences, Makerere University.
10. Amsterdam Centre for Global Child Health, Emma Children's Hospital, Amsterdam University Medical Centres.
11. Amsterdam Institute for Global Health and Development, Amsterdam University Medical Centres.
12. Kamuzu University for Health Sciences (formerly College of Medicine), Blanytre, Malawi.
13. Hospital for Sick Children, Translational Medicine Program and Centre for Global Child Health, Toronto, Canada.
14. Department of Pediatrics, College of Medicine, University of Malawi, Blantyre, Malawi.
15. International Centre for Diarrhoeal Disease Research, Bangladesh (icddr,b), Mohakhali, Dhaka, Bangladesh.
16. Aga Khan University Hospital, Karachi, Sindh, Pakistan.
17. University Joseph KI-ZERBO, Ouagadougou, Burkina Faso.

For peer review only

FULL ADDRESSES OF AUTHORS

Riffat Ara Shawon
Address: Hans Rosling Center for Population Health, Box 351620
3980 15th Ave NE, Seattle, WA 98105
email: shawon@uw.edu

Donna M. Denno
Address: Hans Rosling Center for Population Health, Box 351620
3980 15th Ave NE, Seattle, WA 98105
email: ddenno@uw.edu

Kirkby D. Tickell
Address: Hans Rosling Center for Population Health, Box 351620
3980 15th Ave NE, Seattle, WA 98105
email: kirkbt@uw.edu

Michael Atuhairwe, MBChB
Address: Uganda-Case Western Reserve University Research Collaboration, Makerere University, Upper Mulago Hill Road. Box 7072 Kampala, Uganda
email: michaelatu@gmail.com

Robert H J Bandsma, PhD
Address: Hospital for Sick Children, 555 University Avenue, Toronto, Ontario, Canada
email: robert.bandsma@sickkids.ca

Ezekiel Mupere, PhD
Address: Uganda-Case Western Reserve University Research Collaboration, Makerere University, Upper Mulago Hill Road. Box 7072 Kampala, Uganda.
Email: mupez@yahoo.com

Wieger P. Voskuijl, PhD
Address: Meibergdreef 9, 1105 AZ Amsterdam, Netherlands

For peer review only

email: w.p.voskuijl@amsterdamumc.nl

Emmie Mbale, MBBS
Address: University of Malawi, Chirunga Road, Zomba, Malawi
email: emmiembale@gmail.com

Tahmeed Ahmed. PhD
Address: Dhaka Hospital, Nutrition and Clinical Services Division, icddr,b,
Dhaka, Bangladesh
email: tahmeed@icddrb.org

Md. Jobayer Chisti, PhD
Address: Dhaka Hospital, Nutrition and Clinical Services Division, icddr,b,
Dhaka, Bangladesh
email: chisti@icddrb.org

Ali Faisal Saleem, MBBS
Address: Aga Khan University Hospital, National Stadium Road, Bahadurabad, Karachi, Sindh, Pakistan.
email: ali.saleem@aku.edu

Moses Ngari
Address: KEMRI/Wellcome Trust Research Programme, Clinical Trials Facility, Hospital road, Kilifi, 80108, Kenya
email: mngari@kemri-wellcome.org

Abdoulaye Hama Diallo
Address: Avenue Charles De Gaulle, Ouagadougou, Burkina Faso
email: hamadial@yahoo.fr

James A. Berkley
Address: Oxford OX1 2JD, United Kingdom
email: jberkley@kemri-wellcome.org

For peer review only

Judd L. Walson
Address: Hans Rosling Center for Population Health, Box 351620
3980 15th Ave NE, Seattle, WA 98105
email: walson@uw.edu

Arianna Rubin Means, PhD
Address: Hans Rosling Center for Population Health, Box 351620
3980 15th Ave NE, Seattle, WA 98105
email: aerubin@uw.edu

For peer review only

ABSTRACT

Objectives: This study evaluated the prevalence and correlates of guideline non-adherence for common childhood illnesses in low-resource settings.

Design and setting: We used secondary cross-sectional data from eight healthcare facilities in six Asian and African countries.

Participants: A total of 2,796 children aged 2-23 months hospitalized between November 2016, and January 2019 with pneumonia, diarrhea, or severe malnutrition and without HIV infection were included in this study.

Primary outcome measures: We identified children treated with full, partial, or non-adherent initial inpatient care according to site-specific standard-of-care guidelines for pneumonia, diarrhea, and severe malnutrition (SM) within the first 24-hours of admission. Correlates of guideline non-adherence were identified using generalized estimating equations.

Results: Fully adherent care was delivered to 32% of children admitted with diarrhea, 34% of children with pneumonia, and 28% of children with SM when a strict definition of adherence was applied. Non-adherence to recommendations was most common for oxygen and antibiotics for pneumonia; fluid, zinc, and antibiotics for diarrhea; and vitamin A and zinc for SM. Non-adherence varied by site. Pneumonia guideline non-adherence was more likely among patients with severe disease (Odds Ratio [OR]:1·82; 95% Confidence Interval [CI]:1·38, 2·34) compared to non-severe disease. Diarrhea guideline non-adherence was more likely among lower asset quintile groups (OR:1·16; 95% CI:1·01, 1·35), older children (OR:1·10; 95% CI:1·06, 1·13) and children presenting with wasting (OR:6·44; 95% CI:4·33, 9·57) compared to those with higher assets, younger age, and not wasted.

For peer review only

158 *Conclusions*: Non-adherence to pediatric guidelines was common and associated with older age,
159 disease severity, and comorbidities, and lower household economic status. These findings
160 highlight opportunities to improve guidelines by adding clarity to specific recommendations.

For peer review only

STRENGTHS AND LIMITATIONS OF THIS STUDY

- To our knowledge, this is the largest multi-country study conducted to date evaluating the prevalence of guideline non-adherence for three major causes of pediatric mortality and their sub-conditions: pneumonia, diarrhea, and severe malnutrition.
- Strict interpretation of guidelines with the support of a clinical advisory group revealed contradictions, complexities and gaps within guidelines, including challenges in applying guidelines when children have multiple comorbidities.
- This study uniquely investigated patient-level (ex. socioeconomic status), provider-level (ex. clinician perceived risk at admission that the child may die during hospitalization), and facility-level correlates (ex. patient arrival during weekdays vs weekends/holidays) of guideline non-adherence.
- Non-adherence in this study was measured in hospitals with highly trained and monitored staff which may not be generalizable in all settings.
- Observed non-adherence could be driven by purposeful deviations from guidelines, resulting from resource limitations, environmental factors (e.g., local antibiotic resistance pattern) and vague or unclear guidelines. It was not possible to differentiate purposeful and accidental guideline non-adherence.

For peer review only

INTRODUCTION

Globally, an estimated 14,000 under-five children die every day.[1] Leading causes of mortality in the post-neonatal period include pneumonia (13%) and diarrhea (8%), and undernutrition is an underlying cause in nearly half of child deaths under the age of 5 years.[2,3] Low-resource settings bear a disproportionate burden of child mortality, with over 50% of these deaths occurring in sub-Saharan Africa and one-third in South Asia.[4]

World Health Organization (WHO) normative guidelines offer informed decision support for managing undernutrition, diarrhea, pneumonia and other conditions. These guidelines are often adapted to national contexts for adoption at the country-level. When applied with high fidelity, clinical guidelines can improve health outcomes of targeted populations and reduce case fatality.[5] Yet, guidelines for reducing mortality in children under five, such as the Integrated Management of Childhood Illness and other related WHO guidelines, are inconsistently applied.[6] Guideline non-adherent case management can be driven by inadequate assessment, treatment, and monitoring, local clinical culture, limitations in infrastructure, supply chain challenges, political context, differences in patient-practitioner goals, disease trajectory, patient comorbidities and clarity and applicability of recommendations. Thus, in some circumstances guideline non-adherence may be clinically justified. [7–11]

Evidence from multiple settings indicates that training alone does not affect adherence to guidelines.[12] However, there is minimal information regarding the patient, health service provider and institution specific factors that influence fidelity to clinical guidelines in low-and-middle-income countries (LMICs).[13] We aim to identify the frequency of guideline non-adherence and factors influencing non-adherence during inpatient pediatric treatment for pneumonia, diarrhea, and severe malnutrition (SM) across eight facilities in six LMICs. This

For peer review only

information will be helpful in identifying specific opportunities to support healthcare workers in their daily patient care.

METHODS

Study setting

This analysis utilized de-identified data from the Childhood Acute Illness and Nutrition (CHAIN) study, a multi-site prospective cohort study that identifies risk factors for mortality in hospitalized acutely ill children and after discharge.[14] The CHAIN study took place between November 2016, and January 2019. We include children enrolled in CHAIN aged 2-23 months, hospitalized with pneumonia, diarrhea, or SM (referred to within the guidelines as severe acute malnutrition or SAM) and without HIV infection. Children with malnutrition were deliberately oversampled in CHAIN.

Urban CHAIN sites included hospitals in Karachi (Pakistan), Dhaka (Bangladesh), Kampala (Uganda), Blantyre (Malawi), and Nairobi (Kenya), and rural sites were in Banfora (Burkina Faso), Migori (Kenya), and Matlab (Bangladesh). The sites included public, private non-profit, referral, and district hospitals and often receive patients with complex or severe disease compared to lower-level facilities. Further information about hospital characteristics can be found elsewhere.[15] One CHAIN site, Kilifi County Hospital, Kenya, was omitted from the analysis, as there was a potential conflict between this analysis and ongoing internal guideline adherence auditing processes. Participating Bangladesh facilities were diarrhea specialty centers, also admitting children with other conditions such as pneumonia and SM. In the CHAIN study, sites received a pre-study resource audit, and ongoing performance audit and feedback. Sites were also supported by trained

For peer review only

research clinicians to ensure high standards of clinical care.[16] Thus, in this secondary analysis, we assume clinical staff were highly trained and aware of ongoing monitoring.

Key informants from each site identified institutional or national guidelines available for each condition, and if none, WHO guidelines were used for this analysis (Supplemental Table 1). All sites within the same country reported using the same guidelines. Some facilities might draw from multiple guidelines to manage a child, but for the purposes of this analysis a single and most primary guideline was used as the standard-of-care to abstract relevant diagnostic criteria and treatment recommendations for each condition within each site. To identify children with pneumonia, diarrhea, SM, and sub-conditions we used CHAIN records of clinician diagnoses or, if necessary, we applied guideline diagnostic criteria (Supplemental Table 1).

Conditions and sub-conditions

We evaluated guideline adherence for initial treatment of pneumonia, diarrhea, and SM, the most common conditions across all sites. We also assessed adherence to sub-conditions, including non-severe and severe pneumonia, acute watery diarrhea (AWD) with severe, some and no dehydration, non-severe and severe persistent diarrhea, dysentery, and SM complicated by hypoglycemia, hypothermia, dehydration, severe anemia, measles, micronutrient deficiency, infection, and skin lesions.

We reviewed diagnoses and initial management provided within the first 24-hours of admission. However, we reviewed and counted antibiotic management up to 48-hours to allow for emergence of new clinical information.

For peer review only

Defining adherence to guidelines

We formed a clinical advisory group comprising three CHAIN clinicians, two pediatricians and one general practitioner, simulating a real-world scenario of clinical decision-making with peer support. The group met four times and discussed complex clinical scenarios (e.g., a child with both non-severe pneumonia and SM) to resolve ambiguity guideline interpretation when needed and to allow flexibility in situations of multi-morbidities with conflicting antibiotic recommendations. For example, if a child had both non-severe pneumonia and SM, treatment with broad-spectrum injectable antibiotics (e.g., ampicillin and gentamicin) recommended for SM was considered guideline adherent for non-severe pneumonia, even though pneumonia guidelines recommended an oral antibiotic (e.g., amoxicillin).

Diarrhea, pneumonia, and SM guideline adherence was calculated as the proportion of children initially treated with adherent care for all relevant sub-conditions within 24-hours of admission. We reported the site-specific number and proportion of children managed with full, partial, or non-adherent care. Fully adherent care included providing all treatment components in guidelines (e.g., recommended antibiotics, oral and IV fluid, therapeutic feeds). Non-adherent care indicated that a child was not provided any recommended treatments. Partial adherence indicated that some, but not all, guideline-indicated treatments were provided. We also reported adherence to specific recommendations (e.g., oxygen supplementation for severe pneumonia). The analysis was agnostic to the relative importance of different recommendations.

Statistical analysis

For peer review only

Descriptive statistics were applied to report the proportion of adherence to treatment for conditions (full, partial, and non-adherence) and sub-conditions (adherence and non-adherence) within each site and overall.

The dichotomous outcome for the correlates analysis was any non-adherence to guidelines (including partial non-adherence). We explored patient-specific correlates including age, sex, socio-economic status (composite variable standardized using asset quintiles, derived from the Demographic and Health Survey wealth index), pre-admission care-seeking history , and wasting (weight-for-height Z score <-2).[17] Other patient-specific correlates included comorbidities and presence of WHO and condition-specific danger signs (i.e., obstructed breathing, respiratory distress, shock, cyanosis, convulsions, severe anemia, severe dehydration, profuse watery diarrhea, severe vomiting, impaired consciousness) as assessed by study clinicians.[18] Provider-specific correlates included clinician perceived risk at admission that the child may die during hospitalization. Facility-specific correlates included patient arrival during weekdays vs weekends/holidays according to each country's holiday and weekend conventions.

We determined the association between the pre-specified correlates of interest and guideline non-adherence for each condition by utilizing generalized estimating equations (GEE) with a logit link, with clustering at the hospital-level, robust standard error estimates and an exchangeable correlation structure. This multivariable analysis was evaluated at a 5% alpha level.

Two independent variables had some missingness: care-seeking history (~2% missingness) and clinician risk impression upon admission (~0·1% missingness). We used multiple imputation by chained equation for these variables under the assumption of data missing at random using all non-missing exposure variables in the imputation model. Rubin's rule was used to combine logistic regression outputs of ten iterations.[19] In sensitivity analysis, we repeated all analyses with

For peer review only

complete cases. Because the results did not substantially differ between the two approaches, we presented results from the imputed data.

Kenya's pneumonia guideline excluded children with SM from its treatment recommendations because of conflicting antibiotic recommendations in the national guidelines. Because antibiotic recommendations often conflict between SM and pneumonia recommendations, especially for non-severe pneumonia, we conducted a sensitivity analysis in which we excluded children with SM from pneumonia-specific analyses for all countries. Furthermore, all diarrhea guidelines indicated that diarrhea with comorbid SM requires special management, especially if the child has dehydration. Per the guidelines, SM cases were excluded when calculating diarrhea guideline adherence for all diarrhea sub-conditions. Adherence to rehydration recommendations for children with SM, diarrhea and dehydration was assessed under the SM with dehydration sub-condition.

Because SM assessment in children >6 months does not account for low birth weight or prematurity, we calculated SM guideline adherence amongst children <6 months versus ≥6 months in additional sensitivity analysis. Finally, we conducted a sensitivity analysis for conditions and sub-conditions without any comorbidities overall across all sites. Stata v.16 was used for all analyses.

Role of the funding source

The funding sources had no role in study design, in the collection, analysis, and interpretation of data, in the writing of or decision to submit the report for publication.

Patient and public involvement

Patients or the public were not involved in the design, conduct, reporting, or dissemination plans of our research.

For peer review only

RESULTS

Sample characteristics

Diagnosis and treatment criteria were drawn from thirteen different guidelines (Supplemental Tables 1 & 2) and applied to 2,796 pediatric hospitalizations. A quarter of patient admissions were in Bangladesh (n=708). More than half were male (57%) and 42% were aged 12-23 months (Table 1).

Table 1: Demographic characteristics and prevalence of pneumonia, diarrhea and SM

	Total (N=2,796)	Bangladesh (n=708)	Burkina Faso (n=429)	Kenya (n=533)	Malawi (n=311)	Pakistan (n=349)	Uganda (n=466)
	N (%)[1]	n (%)[1]	n (%)[1]	n (%)[1]	n (%)[1]	n (%)[1]	n (%)[1]
Demographics of children							
Age at admission (months)							
2-5	561 (20)	164 (23)	66 (15)	122 (23)	54 (17)	106 (30)	49 (11)
6-11	1058 (37)	313 (44)	127 (30)	191 (36)	104 (33)	122 (35)	201 (43)
12-23	1177 (42)	231 (33)	236 (55)	220 (41)	153 (49)	121 (35)	216 (46)
Male sex	1588 (57)	426 (60)	245 (57)	297 (56)	172 (55)	190 (54)	258 (55)
Assets Quintiles							
1st (lowest)	464 (17)	58 (8)	141 (33)	155 (29)	92 (30)	1 (0·3)	17 (4)
2nd	538 (19)	106 (15)	183 (43)	71 (13)	91 (29)	4 (0·7)	83 (18)
3rd	582 (21)	140 (20)	72 (17)	113 (21)	59 (19)	30 (9)	168 (36)
4th	596 (21)	187 (26)	19 (4)	111 (21)	34 (11)	107 (31)	138 (30)
5th (highest)	616 (22)	217 (31)	14 (3)	83 (16)	35 (11)	207 (59)	60 (13)
Conditions and sub-conditions							
Pneumonia	1095 (39)	279 (39)	101 (24)	306 (57)	74 (24)	192 (55)	143 (31)
Non-severe	296 (10)	138 (19)	23 (5)	52 (10)	27 (9)	34 (10)	22 (5)
Severe	799 (29)	141 (20)	78 (18)	254 (48)	47 (15)	158 (45)	121 (26)
Diarrhea[2]	935 (32)	576 (81)	71 (16)	103 (20)	60 (20)	49 (14)	76 (16)
AWD w/ severe dehydration	94 (3)	31 (4)	21 (5)	2 (0·4)	10 (3)	11 (3)	19 (4)
AWD w/ some dehydration	130 (5)	50 (7)	5 (1)	46 (9)	5 (2)	10 (3)	14 (3)
AWD w/ no dehydration	683 (23)	494 (70)	45 (10)	54 (10)	40 (13)	16 (5)	34 (7)

For peer review only

Persistent[3]	14 (0·5)		-	-	3 (1)	5 (1)	6 (1)
Severe persistent[3]	8 (0·3)		-	-	2 (1)	4 (1)	2 (0·4)
Dysentery	6 (0·2)	1 (0·1)	-	1 (0.1)	-	3 (0·9)	1 (0·2)
SM[4]	1096 (39)	269 (38)	181 (42)	218 (41)	83 (27)	118 (34)	227 (49)
w/ hypoglycemia	23 (2)	2 (0·3)	5 (1)	6 (1)	-	3 (0·9)	7 (2)
w/ hypothermia	10 (0·9)	-	-	4 (0·8)	2 (0·6)	-	4 (0·9)
w/ dehydration[5]	174 (16)	76 (11)	17 (4)	20 (4)	43 (14)	16 (5)	2 (0·4)
w/ measles	17 (2)	2 (0·3)	-	-	-	8 (2)	7 (2)
w/ severe anemia	31 (3)	-	27 (6)	3 (0·6)	-	1 (0·3)	-
w/ skin lesions	337 (31)	57 (8)	35 (8)	72 (14)	15 (5)	50 (14)	109 (23)
Conditions with Comorbidities[6]							
Pneumonia w/ other comorbidities	905 (32)	253 (36)	96 (22)	228 (43)	54 (17)	157 (45)	116 (25)
Diarrhea w/ other comorbidities	702 (25)	437 (62)	70 (16)	78 (15	30 (10)	41 (12)	46 (10)
SM w/ other comorbidities	996 (36)	267 (38)	168 (39)	206 (39)	68 (22)	115 (33)	172 (37)

[1] The denominator for all percentages is the total number of children treated in each site
[2] Diarrhea with dehydration in children with SM is included under SM
[3] Persistent diarrhea involves no signs of dehydration and severe persistent diarrhea involves signs of dehydration
[4] SM sub conditions are not mutually exclusive. Also, guidelines assume that all SM cases have infection, micronutrient deficiency, and electrolyte imbalance and should be treated accordingly. The other sub-conditions listed in the table for SAM may or may not be present and are not mutually exclusive
[5] Excludes SM with shock/unconsciousness based on guideline recommendations
[6] Comorbidities: bronchiolitis, lower respiratory tract infection, upper respiratory tract infections, tuberculosis, otitis media, asthma, anemia, sickle cell disease, thalassemia, nephrotic syndrome, nephritis, other renal diseases, liver disease, ileus, cardiac disease, sepsis, malaria, soft tissue disease, urinary tract infection, measles, varicella, osteomyelitis, febrile illness, enteric fever, epilepsy, meningitis, encephalopathy, hydrocephalus, developmental delay, cerebral palsy, gastroenteritis, SM (excluding the condition originally being assessed as applicable)
Notes: (1) AWD= Acute Watery Diarrhea, (2) Columns percentages may not exactly be 100% due to rounding, (3) Decimals are rounded to the nearest integer except when the proportion is less than 1%, (4) No exclusions are made in this table for prevalence of conditions and sub-conditions unless noted otherwise, (5) A "-" indicates that there were no cases in the sample with the diagnosis (6) A blank cell indicates there are no specific recommendations for the given condition in the country

Prevalence of conditions and sub-conditions

Approximately one-third of children were admitted with severe pneumonia and 10% with non-severe pneumonia (Table 1). Over half in Kenya (57%, n=533) and Pakistan (55%, n=349) were admitted with pneumonia. About 32% of children across facilities were admitted with diarrhea (excluding children with concurrent SM). The Bangladesh facilities, being diarrhea specialty

For peer review only

hospitals, had the highest proportion of children admitted with diarrhea (81%, n=576) while Pakistan had the lowest (14%, n=349). Across facilities, 39% of children had SM, which is expected given that CHAIN purposefully overenrolled children with wasting. Approximately one-third of children were admitted with at least two of the three conditions: diarrhea, pneumonia and SM. Supplemental Table 3 provides more detail about comorbidities in the sample.

Prevalence of guideline adherence

The guidelines used by countries have significant differences (Supplemental table 1). Prevalence of guideline adherence varied by country and guideline recommendations. Overall, 42% of children with pneumonia were treated with fully adherent care (Table 2 and Supplemental Table 4). The majority of the non-severe pneumonia (54%) cases were treated with full adherence, whereas only 38% of children with severe pneumonia were treated with full adherence. Lower adherence to pneumonia guidelines was driven primarily by non-provision of oxygen when recommended and prescription of different antibiotics, typically broader spectrum, from those recommended (e.g., ceftriaxone rather than ampicillin and gentamicin) (Table 3 and Supplemental Table 5). In sensitivity analyses in which comorbid SM was excluded, we continued to observe partial adherence in most pneumonia cases (58%) (Supplemental Table 6).

Table 2: Summary of guideline adherence, by conditions and sub-conditions

Subtitle: Cell colors indicate which adherence level applied to the largest proportion of patients, by condition and site. Light blue indicates the largest proportion of patients were treated with full adherence, medium blue indicates the largest proportion of patients were treated with partial adherence, and dark blue indicates the largest proportion of patients were treated with non-adherent care

Conditions and sub-conditions	Bangladesh	Burkina Faso	Kenya	Malawi	Pakistan	Uganda	Overall
PNEUMONIA[1]							
Pneumonia (combined)							
Non-severe							

For peer review only

Severe							
DIARRHEA[2]							
Diarrhea (combined)							
AWD w/ severe dehydration							
AWD w/ some dehydration				5a			
AWD w/ no dehydration							
Persistent diarrhea		-	5b				
Severe persistent diarrhea		-	-	5a			
Dysentery		-		-			
SM							
SM (combined)							
W/ hypoglycemia[3]				-			
W/ hypothermia	-	-		5b	-		
W/ dehydration			5b		5b	5b	
W/ severe anemia	-			-			
W/ measles		-	-				
W/ micronutrient deficiency[4] (all SM)							
W/ infection[4] (all SM)							
Requiring nutritional stabilization[4] (all SM)							
W/ skin lesion	-						

[1] For pneumonia, comorbid SM was excluded as per Kenyan pneumonia treatment guidelines
[2] For diarrhea, comorbid SM was excluded, per guidelines
[3] Hypoglycemia treatment applies to all children with SM irrespective of serum glucose level in Uganda, Kenya, Pakistan and Bangladesh, per guidelines
[4] Per guidelines, all children with SM should be treated for presumptive infection, micronutrient deficiency and require nutritional stabilization. Micronutrients include Vit A, zinc, folate, potassium, magnesium
[5a] The proportion of patients treated with partially adherent and nonadherent care were equally high, and the cell is coded to partial adherence
[5b] The propostion of patients treated with fully adherent and nonadherent care were equally high, and the cell is coded to full adherence
Notes: (1) AWD= Acute Watery Diarrhea (2) A "-" indicates that there were no children with the diagnosis (3) a blank cell indicates there are no specific recommendations for a condition in the country, (4) Supplemental Table 4 provides detailed values and proportions for each cell

Across all sites, only 32% children with diarrhea were treated with fully guideline adherent care (Supplemental Table 4). Bangladesh and Burkina Faso had the highest levels of full adherence

For peer review only

while Kenya, Pakistan and Uganda had the lowest (Table 2). The recommendation commonly exhibiting least adherence across diarrhea sub-conditions was provision of zinc (Table 3). Persistent diarrhea and dysentery were typically treated with partially adherent or non-adherent care across sites. Overall, 81% of children were treated will full adherence to the recommendation of withholding antibiotics for acute watery diarrhea without comorbidity. About 41% children with diarrhea were admitted with comorbidities, primarily SM, for which antibiotics were indicated.

Overall, most children with SM were treated with partially adherent care (72%) (Supplemental Table 4). Full adherence was observed in a minority of patients with hypothermia (20%), measles (20%), or skin lesions (5%). In contrast, most patients with SM and hypoglycemia (47%), dehydration (81%), severe anemia (81%), and infection (65%), were treated with fully adherent care. Nutritional stabilization was also started in most children with SM (79%). In sensitivity analysis, we found that full adherence to overall SM guidelines was higher in children 6 months and older (29%) compared to less than 6 months (19%). Recommendations were less detailed for <6 months and simply stated that management should be similar to >6 months old, except some feeding recommendations. Full adherence was lower in <6 months children compared to 6 months and older for recommendations to treat hypoglycemia (43 % vs 54%), dehydration (79% vs 82%), presumed micronutrient deficiency (35% vs 44%), and presumed infection (61% vs 65%). Fully adherent care was higher in <6 months versus in 6 months and older for treatment of skin lesion (15% vs 4%) and severe anemia (100% vs 79%). Adherence to nutritional stabilization recommendations did not differ between the age groups (79% fully adherent).

We conducted sensitivity analyses to describe overall guideline adherence patterns for pneumonia, diarrhea and SM without comorbidities (Supplemental Table 7) and found no appreciable

For peer review only

difference except lower adherence for non-severe pneumonia and higher adherence for micronutrients in SM with dehydration.

Table 3: Number and proportion of children treated with fully guideline adherent care, by specific guideline recommendations

Subtitle: Each cell indicates the number and proportion of children receiving fully adherent care. Cells colored in dark blue indicate <65% full adherence, blue indicate 65-80% full adherence and light blue indicate >80% full adherence

Specific recommendations	Bangladesh	Burkina Faso	Kenya	Malawi	Pakistan	Uganda	Overall
	n (%)						
PNEUMONIA[1]							
Antibiotics for severe pneumonia	107 (76)	36 (46)	102 (68)	42 (89)	0 (0)	98 (81)	329(52)
Antibiotics for non-severe pneumonia	101 (73)	8 (35)	5 (13)	12 (44)	17 (50)	16 (73)	129(54)
Oxygen for severe pneumonia		78 (100) [3]	71 (47) [4]	6 (13)[4]	158 (100) [3]	121 (100) [3]	434(78)
Paracetamol for fever (Severe Pneumonia)		74 (95)		47 (100)	156 (99)	119 (98)	396(98)
Paracetamol for fever (non-severe Pneumonia)				27 (100)		22 (100)	49 (100)
Bronchodilator for wheeze (severe pneumonia)		67 (86)	134 (89)	39 (83)	128 (81)	109 (90)	477(86)
Bronchodilator for wheeze (non-severe pneumonia)			36 (95)	20 (74)		20 (91)	76 (87)
DIARRHEA[2]							
Rehydration for AWD with severe dehydration	9 (29)	14 (67)	2 (100)	3 (30)	8 (73)	9 (47)	45 (52)
Zinc for AWD with severe dehydration	22 (71)	13 (62)	1 (50)	4 (40)	3 (27)	3 (16)	46 (49)
Rehydration for AWD with some dehydration	49 (98)	3 (60)	17 (37)	3 (60)	1 (10)	8 (57)	81(80)
Zinc for AWD with some dehydration		3 (60)	22 (48)	1 (20)	3 (30)	1 (7)	30 (28)
Rehydration for AWD with no dehydration	300 (96)	23 (51)	15 (28)	25 (63)	4 (25)	8 (24)	375 (81)
No antibiotic in AWD without comorbidity	291 (74)	70 (99)	82 (80)	43 (78)	33 (89)	44 (66)	651 (81)
Zinc for AWD with no dehydration		26 (58)	14 (26)	21 (53)	2 (12)	3 (9)	66 (14)
Rehydration for persistent diarrhea		-	2 (50)	2 (67)	3 (60)	0 (0)	7 (44)

Zinc for persistent diarrhea		-	2 (50)	1 (33)	1 (20)	0 (0)	4 (25)
Multi-vitamins for persistent diarrhea[5]		-		0 (0)		0 (0)	0 (0)
Microminerals[6] for persistent diarrhea		-		0 (0)			0 (0)
Rehydration for severe persistent diarrhea		-		1 (50)	3 (75)	0 (0)	4 (50)
Zinc for severe persistent diarrhea		-		0 (0)	0 (0)	0 (0)	3 (30)
Multi-vitamins for severe persistent diarrhea[5]		-		0 (0)		0 (0)	0 (0)
Microminerals[6] for severe persistent diarrhea		-		0 (0)			0 (100)
Rehydration for dysentery		-	0 (0)	-	2 (67)	0 (0)	2 (33)
Zinc for dysentery		-	0 (0)	-	1 (33)	0 (0)	1 (17)
Antibiotics for dysentery	0 (0)	-	1 (100)	-	0 (0)	1 (100)	2 (33)
SM							
Specific antibiotics in SM	236 (88)	41 (23)	161 (74)	62 (75)	2 (2)	206 (91)	710 (65)
Fluid in SM with dehydration	71 (93)	16 (94)	10 (50)	35 (81)	8 (50)	1 (50)	141 (33)
Blood transfusion in SM with severe anemia	-	21 (78)	3 (100)	-	1 (100)	-	25 (81)
Vitamin A in SM with measles		-	-		2 (25)	1 (14)	3 (20)
Micronutrients in SM	143 (53)	68 (38)	114 (52)		22 (19)	72 (32)	471(43)
Therapeutic food in SM	262 (97)	153 (85)	184 (84)	62 (74)	58 (49)	147 (65)	866 (79)
Zinc for skin lesion in SM					8 (16)	0 (0)	8 (5)

[1] For pneumonia, comorbid SM was excluded as per Kenyan pneumonia treatment guidelines
[2] For diarrhea, comorbid SM was excluded, per guidelines
[3] Oxygen is recommended for severe pneumonia with SpO2<90% only
[4] Oxygen is recommended to all children with severe pneumonia
[5] Vitamins include vitamin A, folate, both, and/or multivitamin
[6] Microminerals was an available variable in the CHAIN data without details of what the minerals are
Notes: (1) The denominator for all percentages is the total number of children with a specific condition, (2) AWD= Acute Watery Diarrhea, (3) A "-" indicates there were no cases in the sample with the diagnosis. (4) A blank cell indicates there are no relevant recommendation for a condition in the country, (5) Supplemental Table 5 provides detailed recommendation-specific estimates of adherence levels for all cases

Correlates of guideline non-adherence

In multivariable analysis, children with severe pneumonia were more likely to be treated with guideline non-adherent care (OR:1·81; 95% CI:1·36, 2·41) (Table 4) compared to non-severe pneumonia. For diarrhea, children who were older (OR:1·10; 95% CI:1·06, 1·43) and wasted at

For peer review only

admission (OR:6·69; 95% CI:4·53, 10·17) were more likely to be treated with guideline non-adherent care compared to younger or non-wasted children. Children from higher asset quintiles (OR:0·85; 95% CI:0·73, 0·99) were less likely to receive non-adherent care. For SM, none of the factors appeared to be statistically significant in this analysis.

Table 4: Correlates of pneumonia, diarrhea and SM guideline non-adherence

Variables	Multivariable analysis		
	OR	90% CI	p-value
**PNEUMONIA**			
Older age	0·98	0·96, 1·00	0·113
Male sex	0·85	0·66, 1·09	0·199
Wasted at admission	0·95	0·71, 1·26	0·706
Losing weight/not gaining weight problem detected at admission	0·80	0·58, 1·10	0·166
Presence of danger sign	1·12	0.81, 1·55	0·500
Severe Pneumonia[1]	1·81	1·36, 2·41	<0·001
Previous hospitalization	1·08	0·94, 1·26	0·28
Child likely to die[2]	1·15	0·82, 1·63	0·42
Asset quintile[3]	0·99	0·87, 1·11	0·77
Seeking prior treatment[4]			
Did not seek treatment	1·26	0·79, 2·02	0·332
Sought treatment from shop/pharmacy	1·02	0·76, 1·35	0·911
Sought treatment from traditional healer/herbalist/homeopathist	1·55	0·84, 2·88	0·164
Presence of comorbidity	1·08	0·76, 1·53	0·676
Referred to hospital by health worker	1·23	0·92, 1·66	0·169
Admitted on a holiday	0·87	0·57, 1·34	0·568
**DIARRHEA**			
Older age	1·10	1·06, 1·43	<0·001
Male sex	1·27	0·91, 1·77	0·155
Wasted at admission	6·79	4·53, 10·17	<0·001
Losing weight/not gaining weight problem detected at admission[a]	1·11	0·72, 1·71	0·649
Presence of danger sign	1·50	0·90, 2·50	0·117
Previous hospitalization	1·01	0·81, 1·25	0·933
Child likely to die[2]	0·85	0·54, 1·35	0·496
Asset quintile[3]	0·85	0·73, 0·99	0·033
Seeking prior treatment[4]			
Did not seek treatment	1·03	0·57, 1·89	0·900
Sought treatment from shop/pharmacy	0·91	0·62, 1·34	0·632
Sought treatment from traditional healer/herbalist/homeopathist	0·44	0·19, 1·06	0·066

For peer review only

Presence of comorbidity	0·87	0·61, 1·29	0·527
Referred to hospital by health worker	1·09	0·71, 1·67	0·709
Admitted on a holiday	0·86	0·55, 1·35	0·525
Diarrhea sub-condition[5]			
AWD w/ severe dehydration	1·70	0·96, 3·01	0·067
AWD w/ some dehydration	0·63	0·38, 1·06	0·085
**SM**			
Older age	0·98	0·94, 1·01	0·200
Male sex	1·41	0·93, 2·15	0·106
Losing weight/not gaining weight problem detected at admission	0·59	0·35, 1·01	0·056
Danger sign	0·64	0·35, 1·19	0·161
Previous hospitalization	1·05	0·82, 1·33	0·700
Child likely to die[2]	0·74	0·44, 1·26	0·270
Assets quintile[3]	1·07	0·88, 1·31	0·495
Presence of comorbidity	1·34	0·69, 2·62	0·390
Seeking prior treatment[4]			
Did not seek treatment	2·31	0·77, 7·00	0·137
Sought treatment from shop/pharmacy	1·39	0·80, 2·40	0·243
Sought treatment from traditional healer/herbalist/homeopathist	0·94	0·45, 1·99	0·875
Referred to hospital by health worker	0·84	0·53, 1·35	0·476
Admitted on a holiday	1·04	0·47, 2·31	0·919
SM w/ hypoglycemia	0·54	0·18, 1·59	0·263
SM w/ hypothermia	0·72	0·10, 5·16	0·747
SM w/ dehydration	2·49	1·01, 6·13	0·048
SM w/ skin lesion	1·46	0·89, 2·38	0·132

Reference categories: [1] non-severe pneumonia, [2]child unlikely to die, [3] lowest quintile, [4] sought treatment from a medical facility, [5] AWD w/ no dehydration
[a]Losing weight/not gaining weight problem detected at admission (detected by history taking) and admission MUAC group (detected by anthropometry measurement) were highly correlated. Admission MUAC group was used in the multivariable analysis as this is an objective measure
Notes: (1) AWD=Acute watery diarrhea; OR=Odds Ratio (2) There were not enough cases to run regressions with SM w/ measles and SM w/ severe anemia

393

394 DISCUSSION

395 We examined reported pediatric treatment data from eight health facilities across a range of
396 geographic and ecologic settings in Africa and South Asia to examine adherence to standard-of-
397 care treatment guidelines. Guidelines vary considerably across countries in level of detail and

For peer review only

398 scope of included recommendations. Overall, partial and non-adherence to pneumonia, diarrhea,
399 and SM guidelines was common, although variable by country and across individual
400 recommendations.

401 *Pneumonia guideline adherence*

402 Most children with pneumonia were treated with partial adherence. Lowest adherence was noted
403 for antibiotic recommendations and oxygen supplementation guidance. While amoxicillin is
404 recommended for non-severe pneumonia by all guidelines, broader spectrum antibiotics were
405 typically prescribed in most cases. Hospitalization is not indicated for non-severe pneumonia
406 alone, thus it was likely that children with non-severe pneumonia had other comorbidities or were
407 perceived by a clinician to be at risk of deterioration. A study in Sudan found only 18% of
408 hospitalized children with pneumonia received recommended antibiotic, and that broader spectrum
409 antimicrobials were commonly prescribed.[20] A systematic review of qualitative studies revealed
410 that previous clinical practice, peer practices, fear of patient complications, diagnostic uncertainty,
411 patient comorbidities, desiring a rapid cure, time pressure, and cost saving influenced antibiotic
412 prescription decisions.[21] Clinical guidelines did not emerge as a key driver of initial empiric
413 antibiotic use in the review.[21]

414 Kenyan and Malawian guidelines include oxygen supplementation for all children with severe
415 pneumonia; adherence to this recommendation was uncommon. In contrast, supplementation is
416 recommended based on oxygen saturation levels in the other four countries' guidelines, and full
417 adherence was observed in most children. When faced with vague or broad guidelines, healthcare
418 workers may be required to lean on clinical judgement for decision-making on allocating limited
419 resources such as oxygen.[22] More specific recommendations such as allocation to those at
420 highest risk of clinical deterioration or based upon oxygen saturation or specific clinical signs

For peer review only

could help users prioritize when therapeutics are in short supply and drive higher guideline adherence.[23]

Severe pneumonia treatment recommendations contained more components and were less likely to be fully adhered to compared to non-severe pneumonia. Resource shortages, including oxygen and pulse oximetry, challenge the ability to adhere to recommendations.[24]

Diarrhea guideline adherence

Most children with diarrhea were treated with guideline non-adherent care. Fluid management had the lowest adherence, including non-provision of recommended ORS and IVF. Vitamin A and folate for persistent diarrhea and zinc for any form of diarrhea were also subject to low adherence. Other studies in low-resource settings also reported low adherence to fluid and zinc reccomendations,[25] This may also be driven by clinical practice culture, stock outs, lack of human resources, and low provider knowledge about zinc treatment for diarrhea.[26]

All guidelines considered in this analysis recommend that clinicians should not provide antibiotics for diarrhea in the absence of dysentery, cholera, or an indicated comorbidity. This recommendation was fully adhered to in most cases. However, for patients with dysentery, providers frequently prescribed a different antibiotic than that specified in guidelines. In contrast, a prior study in two Kenyan facilities reported frequent over-prescription of initial antibiotics in non-dysentery uncomplicated diarrhea and under-prescription in dysentery.[27] Our analysis documented and accounted for the presence of comorbidities indicative of initial antibiotic treatment, which is a novel addition to the literature. This might explain some of the over prescription of antibiotics in uncomplicated diarrhea observed in the literature, which typically do not take comorbidities into account.

For peer review only

In this study, children with diarrhea who presented with wasting at were less likely to receive guideline adherent care, compared to non-wasted children. This could result from over-cautiousness of providers in dehydration management among wasted children because of the potentially narrow safety range of fluids in severe wasting.[28] Additionally, children with diarrhea from poorer households were less likely to receive guideline adherent care. Further investigation is necessary to determine if out-of-pocket costs present a barrier to appropriate care. Within our population of children under 2 years old, older children were also less likely to receive adherent care compared to younger children. Evidence from other diseases (malaria and lower respiratory tract infections), similarly, found older children received less guideline adherent care.[29] Some guideline recommendations are different for younger vs older children, especially less than 6 months vs 6 months or older. For example, breast milk replaces ORS in cases requiring oral rehydration. The latter requires a hospital supply or purchase ability by the family which could lead to lower adherence in older children.

SM guideline adherence

Most patients with SM were treated with partially adherent care in this study. A Kenyan study investigating inpatient SM guideline adherence previously reported low adherence for hypoglycemia (15%), hypothermia (5%), and dehydration (31%) management.[30]

Strengths and limitations

This analysis was based on a strict interpretation of guidelines. If antibiotics for pneumonia or dysentery were prescribed based on local sensitivity patterns not described in guidelines, this was considered antibiotic non-adherence. This is true particularly in Pakistan where study physicians report that they purposefully avoid first line antibiotics due to high pathogen resistance prevalence.

For peer review only

465 We also acknowledge the potential influence of unmeasured confounders such as the clinical
466 experience and expertise of different healthcare professionals who treated children within these
467 hospitals in our study. Future research would benefit from systematically collecting such provider-
468 level data to quantitatively explore these relationships and their impact on patient care outcomes.

469 It is possible that treatment was received without documentation as a prescription. For example,
470 zinc or ORS could have been provided directly to caregivers without a prescription and therefore
471 not recorded in the study database. Furthermore, it is possible that delayed documentation could
472 have resulted in non-adherence overestimation. These issues will be explored in future qualitative
473 research. Additionally, data from this study was collected in the context of a cohort study in
474 hospitals with highly trained and monitored staff and with a patient population with a
475 disproportionately high proportion of wasted children; the frequency of guideline non-adherence
476 observed might not be generalizable to a routine clinical care setting as a result.

477 Importantly, adherence in this study is not a proxy for quality of care. Non-adherence to guidelines
478 may not always mean that care was sub-optimal, rather decisions to deviate from guideline are
479 often intentional and valid, driven by complex factors such as institutional norms, resource
480 shortages, or clinical judgement. Measuring quality of care solely based upon adherence to
481 guidelines may mask important nuances not currently captured by guidelines, or unclarity in the
482 guidelines themselves.

483

484 ## CONCLUSION

485 Pediatric treatment guidelines aim to consolidate evidence-based recommendations into
486 straightforward clinical tools, relevant across cadres and settings. This study examined guideline

For peer review only

adherence for pneumonia, diarrhea, and SM and relevant sub-conditions. We found instances of both unexplained and explained guideline non-adherence. These findings highlight opportunities to improve guidelines by increasing specificity for individual recommendations. For example, clarifying oxygen supplementation recommendations for severe pneumonia, or SM management in children under 6 months of age may help improve adherence and clinical outcomes. However, this study also highlights opportunity for strategic nuance, such as providing core guideline recommendations requiring high fidelity with required resource support in addition to ensuring that recommendations are tailored at the local level (e.g., use of antibiotics based on local sensitivity patterns, end-user perspectives, resources and cost considerations of treatment). This analysis also identified areas of complexity and potential confusion in guideline interpretation especially when a child has multiple conditions, underscoring the importance of addressing comorbidities in guidelines. To improve pediatric guideline adherence in low-resource settings, critical review of existing guidelines is necessary to ensure that they fully support clinicians in complex clinical scenarios.

CONTRIBUTORS

ARM, JLW, DMD, and KBT conceptualized the study. ARM, DMD, KBT, and MA contributed to the methodology. ARM and KBT were responsible for data curation. ARM and RAS accessed and verified the data underlying the study. RAS did the formal analysis and prepared the original draft of the manuscript. ARM, JLW, JAB, DMD, KBT, MA, RHB, EMu, WV, EMb, MJC, TA, AFS, MN, and AHD contributed to critically reviewing and editing the manuscript. All authors had full access to all the data in the study and accept responsibility to submit for publication.

For peer review only

DATA SHARING

Any reasonable requests to share data will be considered by the CHAIN consortium. Data requests should be sent to the corresponding author.

DECLARATION OF INTERESTS

We declare no competing interests.

FUNDING

This secondary analysis of the CHAIN data was funded by the National Institutes of Health (NIH), grant number 5R03HD099270-02. The primary CHAIN study was supported by the Bill & Melinda Gates Foundation, grant number OPP1131320. For the purpose of Open Access, the author has applied a CC-BY public copyright license to any author accepted manuscript version arising from this submission.

ACKNOWLEDGEMENT

We thank the CHAIN Network for providing the data for this secondary analysis, and all CHAIN participants, hospitals, and the CHAIN clinical and data management teams. We are grateful to the CHAIN sites who responded to our requests for study site information. This work was supported, in whole or in part, by the Bill & Melinda Gates Foundation [OPP131320]. Under the grant

For peer review only

conditions of the Foundation, a Creative Commons Attribution 4.0 Generic License has already been assigned to the Author Accepted Manuscript version that might arise from this submission.

ETHICAL APPROVAL

The parent CHAIN cohort study protocol was approved by the Oxford Tropical Research Ethics Committee, and the institutional review boards of all partner sites. This secondary analysis was approved by the University of Washington Human Subjects Division (IRB ID #00006878).

For peer review only

REFERENCE

1 UNICEF. Under-five mortality. 2019.https://data.unicef.org/topic/child-survival/under-five-mortality/ (accessed 12 Dec 2021).

2 Black RE, Victora CG, Walker SP, *et al.* Maternal and child undernutrition and overweight in low-income and middle-income countries. *Lancet (London, England)* 2013;**382**:427–51. doi:10.1016/S0140-6736(13)60937-X

3 Hug L, Sharrow D, You D. Levels & Trends in Child Mortality. 2017. https://reliefweb.int/sites/reliefweb.int/files/resources/Levels-and-trends-in-child-mortality-IGME-English_2021.pdf

4 Kuruvilla S, Bustreo F, Kuo T, *et al.* The Global strategy for women's, children's and adolescents' health (2016-2030): a roadmap based on evidence and country experience. *Bull World Health Organ* 2016;**94**:398–400. doi:10.2471/BLT.16.170431

5 Hossain M, Chisti MJ, Hossain MI, *et al.* Efficacy of World Health Organization guideline in facility-based reduction of mortality in severely malnourished children from low and middle income countries: A systematic review and meta-analysis. *J Paediatr Child Health* 2017;**53**:474–9. doi:10.1111/jpc.13443

6 Turner EL, Nielsen KR, Jamal SM, *et al.* A Review of Pediatric Critical Care in Resource-Limited Settings: A Look at Past, Present, and Future Directions. *Front Pediatr* 2016;**4**:5. doi:10.3389/fped.2016.00005

7 Nolan T, Angos P, Cunha AJ, *et al.* Quality of hospital care for seriously ill children in less-developed countries. *Lancet (London, England)* 2001;**357**:106–10. doi:10.1016/S0140-6736(00)03542-X

For peer review only

8 English M, Ntoburi S, Wagai J, *et al.* An intervention to improve paediatric and newborn care in Kenyan district hospitals: Understanding the context. *Implement Sci* 2009;**4**:1–8. doi:10.1186/1748-5908-4-42

9 Solà I, Carrasco JM, Díaz Del Campo P, *et al.* Attitudes and perceptions about clinical guidelines: A qualitative study with spanish physicians. *PLoS One* 2014;**9**. doi:10.1371/journal.pone.0086065

10 Arts DL, Voncken AG, Medlock S, *et al.* Reasons for intentional guideline non-adherence: A systematic review. *Int J Med Inform* 2016;**89**:55–62. doi:10.1016/j.ijmedinf.2016.02.009

11 Hendaus MA, Alhammadi AH, Razig EA, *et al.* Pediatricians' perceptions of clinical practice guidelines. *J Multidiscip Healthc* 2014;**7**:349–54. doi:10.2147/JMDH.S66147

12 Osterholt DM, Onikpo F, Lama M, *et al.* Improving pneumonia case-management in Benin: a randomized trial of a multi-faceted intervention to support health worker adherence to Integrated Management of Childhood Illness guidelines. *Hum Resour Health* 2009;**7**:77. doi:10.1186/1478-4491-7-77

13 Blacklock C, Gonçalves Bradley DC, Mickan S, *et al.* Impact of Contextual Factors on the Effect of Interventions to Improve Health Worker Performance in Sub-Saharan Africa: Review of Randomised Clinical Trials. *PLoS One* 2016;**11**:e0145206.

14 Childhood Acute Illness and Nutrition Network. Childhood Acute Illness and Nutrition (CHAIN) Network: a protocol for a multi-site prospective cohort study to identify modifiable risk factors for mortality among acutely ill children in Africa and Asia. *BMJ Open* 2019;**9**:e028454. doi:10.1136/bmjopen-2018-028454

For peer review only

For peer review only - http://bmjopen.bmj.com/site/about/guidelines.xhtml

15 Tickell KD, Mangale DI, Tornberg-Belanger SN, *et al.* A mixed method multi-country assessment of barriers to implementing pediatric inpatient care guidelines. *PLoS One* 2019;**14**:1–17. doi:10.1371/journal.pone.0212395

16 Diallo AH, Sayeem Bin Shahid ASM, Khan AF, *et al.* Childhood mortality during and after acute illness in sub-Saharan Africa and South Asia - The CHAIN cohort study. *medRxiv* 2021;:2021.11.24.21266806. doi:10.1101/2021.11.24.21266806

17 Rutstein S. Steps to construct new DHS Wealth Index. 2015.https://dhsprogram.com/programming/wealth index/Steps_to_constructing_the_new_DHS_Wealth_Index.pdf

18 WHO. Integrated Management of newborn and Childhood Illness Module: 1. An Introduction to the Integrated Management of Newborn and Childhood Illness (IMNCI). OpenLearnCreate. 2020;:1–8.

19 Azur MJ, Stuart EA, Frangakis C, *et al.* Multiple imputation by chained equations: what is it and how does it work? *Int J Methods Psychiatr Res* 2011;**20**:40–9. doi:10.1002/mpr.329

20 Salih KEM, Bilal JA, Alfadeel MA, *et al.* Poor adherence to the World Health Organization guidelines of treatment of severe pneumonia in children at Khartoum , Sudan. 2014;:1–4.

21 Teixeira Rodrigues A, Roque F, Falcão A, *et al.* Understanding physician antibiotic prescribing behaviour: A systematic review of qualitative studies. *Int J Antimicrob Agents* 2013;**41**:203–12. doi:10.1016/j.ijantimicag.2012.09.003

22 Dauncey JW, Olupot-Olupot P, Maitland K. Healthcare-provider perceptions of barriers to oxygen therapy for paediatric patients in three government-funded eastern Ugandan

For peer review only

hospitals; A qualitative study. *BMC Health Serv Res* 2019;**19**:1–9. doi:10.1186/s12913-019-4129-7

23 Wen B, Brals D, Bourdon C, *et al.* Predicting the risk of mortality during hospitalization in sick severely malnourished children using daily evaluation of key clinical warning signs. *BMC Med* 2021;**19**:1–14. doi:10.1186/s12916-021-02074-6

24 Mvalo T. Need for routine pulse oximetry and oxygen delivery systems in low-income and middle-income countries. *Lancet Glob Heal* 2022;**10**:e301–2. doi:10.1016/S2214-109X(22)00025-0

25 Gathara D, English M, van Hensbroek MB, *et al.* Exploring sources of variability in adherence to guidelines across hospitals in low-income settings: A multi-level analysis of a cross-sectional survey of 22 hospitals. *Implement Sci* 2015;**10**:1–8. doi:10.1186/s13012-015-0245-x

26 Biswas D, Hossin R, Rahman M, *et al.* An ethnographic exploration of diarrheal disease management in public hospitals in Bangladesh: From problems to solutions. *Soc Sci Med* 2020;**260**:113185. doi:10.1016/j.socscimed.2020.113185

27 Rhee C, Aol G, Ouma A, *et al.* Inappropriate use of antibiotics for childhood diarrhea case management - Kenya, 2009-2016. *BMC Public Health* 2019;**19**:1–12. doi:10.1186/s12889-019-6771-8

28 Houston KA, Gibb JG, Maitland K. Intravenous rehydration of malnourished children with acute gastroenteritis and severe dehydration: A systematic review. *Wellcome open Res* 2017;**2**:65. doi:10.12688/wellcomeopenres.12346.1

29 De Bruycker M, Van den Bergh R, Dahmane A, *et al.* Non-adherence to standard

For peer review only

treatment guidelines in a rural paediatric hospital in Sierra Leone. *Public Heal Action* 2013;**3**:118–24. doi:10.5588/pha.12.0060

30 Warfa O, Njai D, Ahmed L, *et al.* Evaluating the level of adherence to Ministry of Health guidelines in the management of severe acute malnutrition at Garissa Provincial General Hospital, Garissa, Kenya. *Pan Afr Med J* 2014;**17**:214. doi:10.11604/pamj.2014.17.214.3821

For peer review only

SUPPLEMENTAL TABLES

For peer review only

For peer review only - http://bmjopen.bmj.com/site/about/guidelines.xhtml

Prevalence and correlates of pediatric guideline non-adherence across six low and middle-income countries

Supplemental Table 1: Comparison of country/institution/WHO guidelines for diagnosis of pneumonia, diarrhea and SM sub-conditions

Variables	Bangladesh	Burkina Faso	Kenya	Malawi	Pakistan	Uganda	WHO
Guideline used in site	Institutional guideline used for pneumonia[1], Diarrhea[2] and SM[3]	WHO guideline used for pneumonia and diarrhea<br><br>National guideline used for SM[4]	National guideline used for pneumonia, diarrhea,[5] and SM[6]	Institutional guidelines used for pneumonia,[7] diarrhea[8], and SM[9]	WHO guideline used for pneumonia<br><br>Institutional guideline used for diarrhea[10]<br><br>National guideline used for SM[11]	National guidelines used for pneumonia, diarrhea, and SM[12]	Pocket Book of Hospital Care for Children[13]
**Pneumonia**							
Severe Pneumonia	Cough or difficulty breathing PLUS at least one of the following:<br><br>-central cyanosis or oxygen saturation <90%<br><br>-severe respiratory distress (e.g. grunting, very severe chest indrawing)<br><br>-signs of pneumonia with a general danger sign (inability to breastfeed or drink, lethargy or unconscious,<br><br>convulsions)	Cough or difficulty breathing PLUS at least one of the following:<br><br>-central cyanosis or oxygen saturation <90%<br><br>-severe respiratory distress (e.g., grunting, very severe chest indrawing)<br><br>-signs of pneumonia with a general danger sign (inability to breastfeed or drink, lethargy or unconscious, convulsions)	Cough or difficulty breathing<br><br>PLUS, one of the danger signs:<br><br>-oxygen saturation <90%,<br><br>-cyanosis<br><br>-inability to drink/breastfeed- AVPU =V/P/U, -grunting	Cough or difficulty breathing PLUS<br><br>-central cyanosis /oxygen saturation <90%,<br><br>-severe respiratory distress (e.g., grunting, severe indrawing, head bobbing, nasal flaring), -convulsion/ loss of consciousness/ feeding/ lethargy	Cough or difficulty breathing PLUS at least one of the following:<br><br>-central cyanosis or oxygen saturation <90%<br><br>-severe respiratory distress (e.g., grunting, very severe chest indrawing)<br><br>-signs of pneumonia with a general danger sign (inability to breastfeed or drink, lethargy or unconscious,<br><br>convulsions)	Cough, fast breathing, AND at least one of the following-<br><br>-central cyanosis or<br><br>-oxygen saturation <90%<br><br>-inability to feed, vomiting everything*<br><br>-convulsions, lethargy, decreased level of consciousness<br><br>-severe respiratory distress (severe chest indrawing, grunting, nasal flaring)<br><br>-extrapulmonary features, e.g., confusion or disorientation	Cough or difficulty breathing PLUS at least one of the following:<br><br>-central cyanosis or oxygen saturation <90%<br><br>-severe respiratory distress (e.g., grunting, very severe chest indrawing)<br><br>-signs of pneumonia with a general danger sign (inability to breastfeed or drink, lethargy or unconscious, convulsions)

For peer review only - http://bmjopen.bmj.com/site/about/guidelines.xhtml

Prevalence and correlates of pediatric guideline non-adherence across six low and middle-income countries

Non-severe Pneumonia	Cough or difficult breathing plus at least one of the following:<br>-fast breathing<br>-lower chest wall indrawing	Cough or difficult breathing plus at least one of the following:<br>-fast breathing<br>-lower chest wall indrawing	Lower chest wall indrawing OR fast breathing	Fast breathing	Cough or difficult breathing plus at least one of the following:<br>-fast breathing<br>-lower chest wall indrawing	Cough, and fast breathing, and mild chest indrawing	Cough or difficult breathing plus at least one of the following:<br>-fast breathing<br>-lower chest wall indrawing
**Diarrhea**							
Acute watery diarrhea with severe dehydration	Acute watery diarrhea and signs of some dehydration PLUS any one of the following:<br>-lethargic/comatose<br>-unable to drink<br>-skin pinch goes back very slowly<br>-radial pulse uncountable or absent*	Diarrhea PLUS any two signs or symptoms of severe dehydration with diarrhea:<br>-lethargic, drowsy or unconscious<br>-sunken eyes<br>-sunken fontanelle<br>-drinks poorly or not able to drink<br>-skin pinch goes back very slowly<br>(>2 seconds)	Diarrhea PLUS unable to drink or AVPU<A, plus sunken eyes, or return of skin pinch >=2 secs	Diarrhea PLUS two or more of the following signs:<br>-lethargy/ unconsciousness<br>-sunken Eyes<br>-unable to drink/drinks poorly<br>-skin pinch goes back very slowly<br>(≥ 2 seconds)	Diarrhea PLUS any two signs or symptoms of severe dehydration<br>-lethargic, drowsy or unconscious<br>-sunken eyes<br>-sunken fontanelle<br>-drinks poorly or not able to drink<br>-skin pinch goes back very slowly<br>(>2 seconds)	Diarrhea PLUS clinical features of severe dehydration<br>-lethargic, drowsy or unconscious<br>-sunken eyes<br>-sunken fontanelle<br>-drinks poorly or not able to drink<br>-skin pinch goes back very slowly<br>(>2 seconds)	Diarrhea PLUS any two signs or symptoms of severe dehydration<br>-lethargic, drowsy or unconscious<br>-sunken eyes<br>-sunken fontanelle<br>-drinks poorly or not able to drink<br>-skin pinch goes back very slowly<br>(>2 seconds)
Acute watery diarrhea with Some dehydration	Acute watery diarrhea PLUS at least two signs including one (*) sign<br>-irritable/less active*<br>-sunken eyes<br>-dry tongue<br>-thirsty (drinks eagerly)<br>-skin pinch goes back slowly*<br>-reduced radial pulse*	Diarrhea PLUS if the child has two or more of the following signs, he or she has some dehydration:<br>-restlessness or irritability<br>-thirsty and drinks eagerly<br>-sunken eyes<br>-skin pinch goes back slowly.<br>If a child has only one of the above	Diarrhea PLUS able to drink adequately but 2 or more of: Sunken eyes, return of skin pinch 1-2 secs, restlessness/ irritability	Diarrhea PLUS two or more of the following signs:<br>-restlessness/ irritability<br>-sunken eyes<br>-drinks eagerly/thirsty<br>-skin pinch goes back slowly	Diarrhea PLUS if the child has two or more of the following signs, he or she has some dehydration:<br>-restlessness or irritability<br>-thirsty and drinks eagerly<br>-sunken eyes<br>-skin pinch goes back slowly<br>If a child has only one of the above signs and one of the signs of	Diarrhea PLUS clinical features of some dehydration:<br>-restless, irritable<br>-sunken Eyes<br>-sunken fontanelle<br>- drinks eagerly, thirsty<br>-skin pinch goes back slowly; <2 seconds	Diarrhea PLUS if the child has two or more of the following signs, he or she has some dehydration:<br>-restlessness or irritability<br>-thirsty and drinks eagerly<br>-sunken eyes<br>-skin pinch goes back slowly.<br>If a child has only one of the above signs and one of the signs of<br>severe dehydration (e.g. restlessness or irritable and drinking poorly), then the<br>child also has some dehydration

For peer review only

Prevalence and correlates of pediatric guideline non-adherence across six low and middle-income countries

		signs and one of the signs of<br>severe dehydration (e.g., restlessness or irritable and drinking poorly), then the<br>child also has some dehydration			severe dehydration (e.g., restlessness or irritable and drinking poorly), then the<br>child also has some dehydration		
Acute watery diarrhea with no dehydration	Acute watery diarrhea PLUS normal awareness condition, eyes, tongue, thirst, skin pinch, radial pulse	Diarrhea PLUS no sign of dehydration	Diarrhea PLUS fewer than 2 of the signs of dehydration	Diarrhea PLUS not enough signs to classify as some or severe dehydration	Diarrhea PLUS no sign of dehydration	Diarrhea PLUS clinical features of no dehydration:<br>-alert<br>-eyes not sunken<br>-fontanelle not sunken<br>-drinks normally<br>-skin pinch goes back immediately	Diarrhea PLUS no sign of dehydration
Persistent diarrhea	-	Diarrhea, with or without blood, that begins acutely and lasts for ≥ 14 days with no signs of dehydration	Diarrhea, with or without blood, that begins acutely and lasts for ≥ 14 days with no signs of dehydration	Diarrhea, with or without blood, that begins acutely and lasts for ≥ 14 days with no signs of dehydration	Diarrhea, with or without blood, that begins acutely and lasts for ≥ 14 days with no signs of dehydration	Diarrhea, with or without blood, that begins acutely and lasts for ≥ 14 days with no signs of dehydration	Diarrhea, with or without blood, that begins acutely and lasts for ≥ 14 days
Severe Persistent diarrhea	-	When there is some or severe dehydration, persistent diarrhea is classified as 'severe'	When there is some or severe dehydration, persistent diarrhea is classified as 'severe'	When there is some or severe dehydration, persistent diarrhea is classified as 'severe'	When there is some or severe dehydration, persistent diarrhea is classified as 'severe'	When there is some or severe dehydration, persistent diarrhea is classified as 'severe'	When there is some or severe dehydration, persistent diarrhea is classified as 'severe'
Dysentery	Diarrhea PLUS<br>-febrile≥ 38°C<br>-passes bloody or mucoid stools*<br>-has straining or tenesmus.	Blood mixed with the stools	Blood mixed with the stools	Frequent loose stools with visible red blood	Blood mixed with the stools	Bloody diarrhea, visible blood and mucus	Blood mixed with the stools

For peer review only - http://bmjopen.bmj.com/site/about/guidelines.xhtml

**SM**							
SM	Bilateral oedema of both feet OR WFH/L <-3 z scores OR MUAC<125mm for children>=12 months<br>For <12 months, WLZ<-3 OR bipedal oedema	Bilateral pitting oedema OR severe wasting (WHZ< -3 z scores OR MUAC<115mm)<br>For <6 months, WLZ<-3 OR bipedal oedema OR visible wasting	Bilateral pitting oedema OR severe wasting (WHZ< -3 z scores OR MUAC<115mm)<br>For <6 months, WLZ<-3 OR bipedal oedema OR visible wasting	Bilateral pitting oedema OR severe wasting (WHZ< -3 z scores OR MUAC<115mm)<br>For <6 months, WLZ<-3 OR bipedal oedema	Bilateral oedema of both feet OR WFH/L <-3 z scores OR MUAC<115mm OR visible severe wasting<br>For <6 months, WLZ<-3 OR bipedal oedema OR visible wasting	Oedema of both feet OR WFH/L <-3 z scores OR MUAC<115mm OR visible severe wasting<br>For <6 months, WLZ<-3 OR bipedal oedema OR visible wasting	Bilateral oedema of both feet OR WFH/L <-3 z scores OR MUAC<115mm OR visible severe wasting<br>For <6 months, WLZ<-3 OR bipedal oedema OR visible wasting
Hypoglycemia in SM	Blood sugar <3 mmol/L	Blood sugar <3 mmol/L	Blood sugar <3 mmol/L	Blood sugar <3 mmol/L	Blood sugar <3 mmol/L	Blood sugar <3 mmol/L	Blood sugar <3 mmol/L
Hypothermia in SM	Axillary temperature <35c and rectal temperature <35·5c	Axillary temperature <35c and rectal temperature <35·5c	Axillary temperature <35c and rectal temperature <35·5c	Axillary temperature <35c	Axillary temperature <35c and rectal temperature <35·5c	Axillary temperature <35c and rectal temperature <35·5c	Axillary temperature <35c and rectal temperature <35·5c
Dehydration in SM	Signs of some or severe dehydration	Recent fluid loss, recent change in gaze, sunken eye, absence of superficial veins	All children with watery diarrhea or reduced urine output	Signs of some or severe dehydration	All children with watery diarrhea or reduced urine output	All children with watery diarrhea or reduced urine output	All children with watery diarrhea or reduced urine output
Severe Anemia in SM	Hb<5g/dl	Hb<4g/dl	Hb<4g/dl	Hb<4 OR Hb<6 with respiratory distress	Hb<4g/dl OR Hb 4-6g/dl with respiratory distress	Hb<4g/dl OR Hb 4-6g/dl with respiratory distress	Hb<4g/dl OR Hb 4-6g/dl with respiratory distress
Skin lesion in SM	Weeping skin lesions	Skin lesions in kwashiorkor	Dermatosis (patches of skin abnormally light or dark, shedding of skin, ulceration of skin and/or weeping<br>lesions)	No information	Hypo-or hyperpigmentation, Desquamation, Ulceration, Exudative lesions often with secondary infections, including Candida	Skin lesions in kwashiorkor	Hypo-or hyperpigmentation, Desquamation, Ulceration, Exudative lesions often with secondary infections, including Candida
Infection in SM	Most children with SM in in-patient care have infections and all are routinely treated	All children with SM should be treated for infection even in absence of clinical signs	Nearly all children with SM have bacterial infections. (all children should routinely receive antibiotic)	Typical signs of infections are absent. (so, antibiotics recommended in all children)	Most of the children with SM in in-patient care have infections and all are routinely treated	Assume children with SM have infection	Assume children with SM have infection
SM with measles	SM with measles	SM with measles	SM with measles	-	SM with measles	SM with measles	SM with measles

1. Dhaka Hospital. icddr,b Treatment Guidelines for Community Acquired Pneumonia. :1.

2. Nutrition & Clinical Services Division , icddr , b Treatment for Cholera and other Diarrheas.

For peer review only - http://bmjopen.bmj.com/site/about/guidelines.xhtml

Prevalence and correlates of pediatric guideline non-adherence across six low and middle-income countries

3. Ahmed T. Manual for Standardized Management of Severely Malnourished Children. 1–49 p.

4. Ministère de la Santé. Protocole National: Prise En Charge Integree De La Malnutrition Aiguë (Pcima), Burkina Faso. 2014;154.

5. Ministry of Health Kenya. Basic Paediatric Protocols. 2016.

6. Ministry of Health Kenya. National Guidelines for Integrated Management of Acute Malnutrition. 2009.

7. Queen Elizabeth Central Hospital. Electronic protocols for the management of common childhood illnesses in Malawi: Pneumonia. 2018.

8. Queen Elizabeth Central Hospital. Electronic protocols for the management of common childhood illnesses in Malawi: Diarrhea. 2018.

9. Voskuijl W. Queen Elizabeth Central Hospital. Electronic protocols for the management of common childhood illnesses in Malawi: Malnutrition. 2018;

10. Pakistan Pediatric Association. Diarrhea in Children. 2010. p. 1–14.

11. Ministry of Health Pakistan. National Guidelines for the management of acute malnutrition among children under five and pregnant and lactating women Ministry of Health. 2009.

12. Ministry of Health Uganda. Uganda Clinical Guidelines. 2016.

13. WHO. Pocket Book of Hospital Care for Children. World Health Organization; 2013.

* These variables were not available in the dataset. The coding was done with available variables only

Note: All of the diagnosis of conditions and sub-conditions were determined based on signs and symptoms and guideline criteria with the exception of two conditions: diarrhea, pneumonia, and one sub-condition: measles (in SM). These three diagnoses were determined based upon clinician diagnosis

For peer review only

For peer review only - http://bmjopen.bmj.com/site/about/guidelines.xhtml

Supplemental Table 2: Comparison of country/institution/WHO guidelines for management of pneumonia, diarrhea and SM sub-conditions

Variables	Bangladesh	Burkina Faso	Kenya	Malawi	Pakistan	Uganda	WHO
Guideline used in site	Institutional guideline used for pneumonia[1], Diarrhea[2] and SM[3]	WHO guideline used for pneumonia and diarrhea<br><br>National guideline used for SM[4]	National guideline used for pneumonia, diarrhea,[5] and SM[6]	Institutional guidelines used for pneumonia,[7] diarrhea[8], and SM[9]	WHO guideline used for pneumonia<br><br>Institutional guideline used for diarrhea[10]<br><br>National guideline used for SM[11]	National guidelines used for pneumonia, diarrhea, and SM[12]	Pocket Book of Hospital Care for Children[13]
**Pneumonia**							
Severe Pneumonia	Ampicillin + Gentamicin	Ampicillin (or Benzylpenicillin) and Gentamicin<br><br>Give oxygen if saturation <90%<br><br>If fever: paracetamol<br><br>If wheeze: bronchodilator	Penicillin and Gentamicin<br><br>Give oxygen<br><br>If wheeze: bronchodilators	X-pen + Gentamicin<br><br>Give oxygen<br><br>If fever: paracetamol<br><br>If wheeze: salbutamol	Ampicillin (or Benzylpenicillin) and Gentamicin<br><br>Give oxygen if saturation <90%<br><br>If fever: paracetamol<br><br>If wheeze: salbutamol	Ampicillin (or Benzylpenicillin) and Gentamicin<br><br>Give oxygen if saturation <90%<br><br>If wheeze: salbutamol<br><br>If fever- paracetamol<br><br>If convulsion: diazepam or phenobarbital	Ampicillin (or Benzylpenicillin) and Gentamicin<br><br>Give oxygen if saturation <90%<br><br>If fever: paracetamol<br><br>If wheeze: salbutamol
Non-severe Pneumonia	Amoxicillin	Amoxicillin	Amoxicillin<br><br>If wheeze: bronchodilators	Amoxicillin<br><br>If fever: paracetamol<br><br>If wheeze: salbutamol	Amoxicillin	Amoxicillin<br><br>If fever- paracetamol<br><br>If wheeze: salbutamol	Amoxicillin
**Diarrhea**							
Diarrhea with severe dehydration	IV fluid I/V solution containing sodium, potassium, chloride and bicarbonate (e.g. Ringer's Lactate/ cholera saline)<br><br>Zinc	IV Ringers lactate or normal saline<br><br>Zinc (once the child starts to improve sufficiently)	IV Ringers' lactate or NG rehydration<br><br>Zinc<br><br>Antimicrobials are not indicated unless there is	IV Ringers lactate or normal saline<br><br>Zinc (when severe dehydration is corrected)	IV Ringers lactate or normal saline<br><br>Zinc (once the child starts to improve sufficiently)	IV fluid or ORS by NG<br><br>Zinc	IV Ringers lactate or normal saline<br><br>Zinc (once the child starts to improve sufficiently)

For peer review only - http://bmjopen.bmj.com/site/about/guidelines.xhtml

Prevalence and correlates of pediatric guideline non-adherence across six low and middle-income countries

	Antibiotic only if necessary		dysentery or proven amoebiasis or giardiasis.			Avoid unnecessary antibiotic	
Diarrhea with some dehydration	ORS	ORS<br>After reassessment, if there is no dehydration, give zinc supplement	ORS, zinc<br>Antimicrobials are not indicated unless there is<br>dysentery or proven amoebiasis or giardiasis.	ORS, zinc	ORS<br>After reassessment, if there is no dehydration, give zinc supplement	ORS, zinc	ORS<br>After reassessment, if there is no dehydration, give zinc supplement
Diarrhea with no dehydration	ORS	ORS, zinc	ORS, zinc	ORS or clean water, zinc	ORS, zinc	ORS, zinc	ORS, zinc
Persistent diarrhea with no dehydration	-	Fluid, zinc, vitamin A, minerals	ORS, zinc	ORS, zinc, vitamin A, folate, multimineral	Fluid, zinc, vitamin A, minerals	ORS, zinc, vitamin A	Fluid, zinc, vitamin A, minerals
Persistent diarrhea with some or severe dehydration	-	Fluid, zinc, vitamin A, folate, multimineral	Fluid, zinc	Fluid, zinc, vitamin A, folate, multimineral	Fluid, zinc, vitamin A, folate, multimineral	Fluid, zinc, vitamin A	Fluid, zinc, vitamin A, folate, multimineral
Dysentery	Azithromycin if Shigellosis; Metronidazole if Amoebiasis<br>Continue Ampicillin, Ciprofloxacin or Ceftriaxone if indicated for other causes	Ciprofloxacin if antibiotic sensitivity<br>is unknown. If local antimicrobial sensitivity is known, follow<br>local guidelines.<br>ceftriaxone to severely ill children	Ciprofloxacin	Azithromycin or Ciprofloxacin	Ciprofloxacin or Cefixime	Ciprofloxacin	Ciprofloxacin if antibiotic sensitivity<br>is unknown. If local antimicrobial sensitivity is known, follow<br>local guidelines.<br>ceftriaxone to severely ill children
**SM**							
Hypoglycemia in SM	Oral or IV glucose	Feed or 10% glucose/ sucrose	All children with SM are at risk and should be given a feed or 10% glucose/ sucrose immediately on admission	If hypoglycemia, give a feed and oral or 10% IV glucose immediately on admission	Assume all children with SM are hypoglycemic. Give a feed or 10% glucose/ sucrose immediately on admission	All children with SM are at risk and should be given a feed or 10% glucose/ sucrose immediately on admission	All children with SM are at risk and should be given a feed or 10% glucose/ sucrose immediately on admission

For peer review only - http://bmjopen.bmj.com/site/about/guidelines.xhtml

Prevalence and correlates of pediatric guideline non-adherence across six low and middle-income countries

Hypothermia in SM	Keep warm<br>Ensure feeding	Give a feed or 10% glucose/ sucrose, warm the child, give antibiotics	Keep warm	Warm the child	Warm the child, feed 2 hourly	Feed immediately as in hypoglycemia, warm the child, give antibiotics	Feed immediately as in hypoglycemia, warm the child, give antibiotics
Dehydration in SM	ORS/IV fluid	ReSoMal except in case of shock/ unconscious	ReSoMal except in case of shock/ unconscious	ReSoMal/ORS	ReSoMal except in case of shock/ unconscious	ReSoMal except in case of shock/ unconscious	ReSoMal except in case of shock/ unconscious
Severe Anemia in SM	Blood transfusion<br>or<br>packed blood cell*	Blood transfusion + furosemide	Blood transfusion	Blood transfusion	Blood transfusion + furosemide	Blood transfusion + furosemide	Blood transfusion + furosemide
Skin lesion in SM	Potassium permanganate solution*	No zinc	No zinc	Cloxacillin or Flucloxacillin if infected skin lesion	Zinc supplementation	Zinc supplementation + ointments	Zinc supplementation
Infection in SM *(applicable to all inpatients for prevention or management of infection)*	Broad spectrum antibiotic	Ceftriaxone OR Ceftriaxone + Gentamicin OR Ceftriaxone + Metronidazole	Penicillin OR Ampicillin PLUS Gentamicin	Benzylpenicillin PLUS Gentamicin	Penicillin OR Ampicillin PLUS Gentamicin	Benzylpenicillin OR Ampicillin PLUS Gentamicin	Penicillin OR Ampicillin PLUS Gentamicin
Micronutrient deficiency in SM *(applicable to all children with SM)*	Vit A, zinc, folate, multivitamin, potassium*, magnesium*	If clinical anemia, give folic acid	If child not on premixed food the give folic acid	No information	Give folic acid, multivitamin, zinc, copper to all children with SM	If child not on premixed food the give folic acid, multivitamin, zinc, copper	If child not on premixed food the give folic acid, multivitamin, zinc, copper
SM requiring nutritional stabilization *(applicable to all children with SM)*	Liquid diet such as milk suji	F-75,<br>(for children<6 months, F-75 OR diluted F-100 OR breastmilk)	F-75,<br>(for children<6 months, F-75 OR diluted F-100 OR breastmilk)	F-75, to all children	F-75,<br>(for children<6 months, F-75 OR diluted F-100 OR breastmilk)	F-75,<br>(for children<6 months, F-75 OR diluted F-100 OR breastmilk)	F-75,<br>(for children<6 months, F-75 OR diluted F-100 OR breastmilk)
SM with measles	Not mentioned	Vitamin A	Vitamin A	No information	Vitamin A	Vitamin A	Vitamin A

1. Dhaka Hospital. icddr,b Treatment Guidelines for Community Acquired Pneumonia. :1.
2. Nutrition & Clinical Services Division , icddr , b Treatment for Cholera and other Diarrheas.
3. Ahmed T. Manual for Standardized Management of Severely Malnourished Children. 1–49 p.
4. Ministère de la Santé. Protocole National: Prise En Charge Integree De La Malnutrition Aiguë (Pcima), Burkina Faso. 2014;154.
5. Ministry of Health Kenya. Basic Paediatric Protocols. 2016.

For peer review only - http://bmjopen.bmj.com/site/about/guidelines.xhtml

Prevalence and correlates of pediatric guideline non-adherence across six low and middle-income countries

6. Ministry of Health Kenya. National Guidelines for Integrated Management of Acute Malnutrition. 2009.

7. Queen Elizabeth Central Hospital. Electronic protocols for the management of common childhood illnesses in Malawi: Pneumonia. 2018.

8. Queen Elizabeth Central Hospital. Electronic protocols for the management of common childhood illnesses in Malawi: Diarrhea. 2018.

9. Voskuijl W. Queen Elizabeth Central Hospital. Electronic protocols for the management of common childhood illnesses in Malawi: Malnutrition. 2018;

10. Pakistan Pediatric Association. Diarrhea in Children. 2010. p. 1–14.

11. Ministry of Health Pakistan. National Guidelines for the management of acute malnutrition among children under five and pregnant and lactating women Ministry of Health. 2009.

12. Ministry of Health Uganda. Uganda Clinical Guidelines. 2016.

13. WHO. Pocket Book of Hospital Care for Children. World Health Organization; 2013.

* These variables were not available in the dataset. The coding was done with available variables only

For peer review only

Prevalence and correlates of pediatric guideline non-adherence across six low and middle-income countries

Supplemental Table 3: Overlap between pneumonia, diarrhea, SM and other clinical diagnoses

Overlapping diagnoses	Conditions n (%)		
	Pneumonia (N=1095)	Diarrhea (N=1014)	SM (N=1096)
Pneumonia	-	418 (32)	384 (35)
Diarrhea	418 (38)	-	524 (48)
SM	384 (35)	524 (41)	-
Bronchiolitis	77 (7)	14 (1)	21 (2)
Upper respiratory tract infection	11 (1)	59 (5)	58 (5)
Tuberculosis	18 (2)	4 (0·3)	30 (3)
Otitis media	7 (0·6)	4 (0·3)	7 (0·6)
Asthma	4 (0·4)	-	1 (0·1)
Anemia	203 (19)	179 (14)	211 (19)
Sickle cell disease	5 (0·5)	1 (0·1)	4 (0·4)
Thalassemia	1 (0·1)	1 (0·1)	1 (0·1)
Renal impairment	-	1 (0·1)	3 (0·3)
Nephrotic syndrome	-	-	2 (0·2)
Liver disease	-	-	1 (0·1)
Ileus	2 (0·2)	2 (0·2)	1 (0·1)
Congenital cardiac disease	1 (0·1)	-	1 (0·1)
Sepsis	91 (8)	138 (11)	165 (15)
Malaria	108 (10)	96 (7)	137 (13)
Soft tissue infection	2 (0·2)	2 (0·2)	4 (0·4)
Urinary tract infection	3 (0·3)	8 (0·7)	3 (0·3)
Measles	48 (4)	13 (1)	17 (2)
Osteomyelitis	-	-	1 (0·1)
Febrile illness	1 (0·1)	21 (2)	4 (0·4)
Enteric fever	10 (0·9)	6 (0·7)	28 (3)
Epilepsy	1 (0·1)	-	-
Meningitis	32 (3)	14 (1)	25 (2)
Encephalopathy	2 (0·2)	4 (0·3)	2 (0·2)
Hydrocephalous	2 (0·2)	-	2 (0·2)
Developmental delay	2 (0·2)	3 (0·2)	10 (1)
Cerebral Palsy	4 (0·4)	2 (0·2)	5 (0·5)
Note: The denominator is all cases of the syndrome in column for all proportions			

For peer review only

Supplemental Table 4: Guideline adherence for conditions and sub-conditions, by country

Conditions and sub-conditions	Bangladesh n %			Burkina Faso n %			Kenya n %			Malawi n %			Pakistan n %			Uganda n %			Overall n %		
	Full	Partial	None	Full	Partial	None	Full	Partial	None	Full	Partial	None	Full	Partial	None	Full	Partial	None	Full	Partial	None
**PNEUMONIA[1]**	208<br>75	-	71<br>25	36<br>36	50<br>50	15<br>15	40<br>21	147<br>78	1<br>0·5	13<br>18	61<br>82	-	17<br>9	157<br>82	18<br>9	101<br>71	42<br>29	-	415<br>42	457<br>47	105<br>11
Non-severe	101<br>73	-	37<br>27	8<br>35	-	15<br>65	4<br>11	33<br>87	1<br>3	10<br>37	17<br>63	-	17<br>50	-	17<br>50	14<br>64	8<br>36	-	154<br>54	58<br>21	70<br>24
Severe	107<br>76	-	34<br>24	28<br>36	50<br>64	-	36<br>24	114<br>76	-	3<br>6	44<br>94	-	-	158<br>100	1<br>1	87<br>72	34<br>28	-	261<br>38	400<br>57	35<br>5
**DIARRHEA[2]**	259<br>66	132<br>33	4<br>1	36<br>51	9<br>13	26<br>37	2<br>2	49<br>46	56<br>52	16<br>27	24<br>40	20<br>33	6<br>12	18<br>37	25<br>51	4<br>5	23<br>30	49<br>64	323<br>32	255<br>25	435<br>43
AWD w/ severe dehydration	6<br>19	22<br>71	3<br>10	12<br>57	3<br>14	6<br>29	-	2<br>100	-	-	6<br>60	4<br>40	3<br>27	5<br>46	3<br>27	3<br>16	6<br>32	10<br>53	24<br>26	44<br>47	26<br>28
AWD w/ some dehydration	35<br>70	15<br>30	-	3<br>60	-	2<br>40	-	25<br>54	21<br>46	1<br>20	2<br>40	2<br>40	1<br>10	2<br>20	7<br>70	1<br>7	7<br>50	6<br>43	41<br>33	51<br>39	38<br>29
AWD w/ no dehydration	218<br>69	95<br>30	1<br>0·3	21<br>47	6<br>13	18<br>40	-	21<br>39	33<br>61	15<br>38	13<br>33	12<br>30	2<br>13	2<br>13	12<br>75	-	9<br>26	25<br>74	256<br>51	146<br>29	101<br>20
Persistent				-	-	-	2<br>50	-	2<br>50	-	2<br>67	1<br>33	-	4<br>80	1<br>20	-	-	6<br>100	2<br>11	6<br>33	10<br>56
Severe persistent				-	-	-	-	-	-	-	1<br>50	1<br>50	-	3<br>75	1<br>25	-	-	2<br>100	-	4<br>50	4<br>50
Dysentery	-	-	1<br>100	-	-	-	-	1<br>100	-	-	-	-	-	2<br>67	1<br>33	-	1<br>100	-	-	4<br>67	2<br>33
**SM[3]**	136<br>51	133<br>49	-	14<br>8	167<br>92	-	80<br>37	138<br>63	-	49<br>34	34<br>41	-	-	118<br>100	-	23<br>10	204<br>90	-	302<br>28	794<br>72	-
W/ hypoglycemia[4]	-	2<br>100	-	181<br>100	-	-	90<br>41	114<br>52	14<br>6	-	-	-	-	24<br>20	94<br>80	81<br>36	134<br>59	12<br>5	352<br>47	274<br>37	120<br>16
W/ hypothermia	-	-	-	-	-	-	1<br>25	-	3<br>75	1<br>50	-	1<br>50	-	-	-	-	4<br>100	-	2<br>20	4<br>40	4<br>40

For peer review only

Prevalence and correlates of pediatric guideline non-adherence across six low and middle-income countries

SM with dehydration	71<br>*93*	-	5<br>*7*	16<br>*94*	-	1<br>*6*	10<br>*50*	-	10<br>*50*	35<br>*81*	-	8<br>*19*	8<br>*50*	-	8<br>*50*	1<br>50	-	1<br>*50*	141<br>*81*		33<br>*19*
SM with severe anemia	-	-	-	21<br>*78*	-	6<br>*22*	3<br>*10*<br>*0*	-	-	-	-	-	1<br>*100*	-	-	-	-	-	25<br>*81*		6<br>*19*
SM with active measles				-	-	-	-	-	-				2<br>*25*	-	6<br>*75*	1<br>*14*	-	6<br>*86*	3<br>*20*		12<br>*80*
W/ micronutrient deficiency[5]	143<br>*53*	81<br>30	45<br>*17*	1<br>*0·9*	-	113<br>*99*	11<br>4<br>*52*	-	104<br>*48*				22<br>*19*	6<br>*5*	90<br>*76*	72<br>*32*	-	155<br>*68*	352<br>*37*	87<br>*9*	507<br>*54*
W/ infection[5]	238<br>*88*	-	31<br>*12*	41<br>*23*	48<br>*27*	92<br>*51*	16<br>1<br>*74*	36<br>*16*	21<br>*10*	62<br>*75*	-	21<br>*25*	2<br>*2*	1<br>*1*	115<br>*97*	206<br>*91*	7<br>*3*	14<br>*6*	710<br>*65*	92<br>*8*	294<br>*27*
Nutritional stabilization[5]	262<br>*97*	1<br>*0·4*	6<br>*2*	153<br>*85*	-	28<br>*16*	18<br>4<br>*84*	-	34<br>*16*	62<br>*75*	-	21<br>*25*	58<br>*49*	-	60<br>*51*	147<br>*65*	-	80<br>*35*	866<br>*79*	1<br>*0·1*	229<br>*21*
W/ skin lesion	[6]	[6]	[6]										8<br>*16*	-	42<br>*84*	-	-	109<br>*100*	8<br>*5*	-	151<br>*95*

[1] For pneumonia, comorbid SM was excluded as per Kenyan pneumonia treatment guidelines

[2] For diarrhea w/dehydration, comorbid SM was excluded, per guidelines

[3] Total SM cases in any adherence category may not be equal to the summation of all SM sub-conditions in that category since these are not mutually exclusive.

[4] Hypoglycemia treatment applies to all children with SM in Uganda, Kenya, and Pakistan as per guidelines irrespective of blood glucose level.

[5] Per guidelines, all children with SM should be treated for Infection and micronutrient deficiency and all children require nutritional stabilization. Micronutrients include Vit A, Zinc, Multivitamin, & Folic acid if therapeutic feed not given

[6] Variable for skin lesion treatment in Bangladesh not available in the data

Notes: (1) The denominator for all percentages is the total number of children with a specific condition, (2) AWD= Acute Watery Diarrhea, (3) A "-" indicates that there were no cases in the sample with the diagnosis (4) a blank cell indicates there are no relevant recommendation for a condition in the country, (5) In case of a tie, comparatively higher adherence level between the tie categories is assumed to be the majority

For peer review only

Prevalence and correlates of pediatric guideline non-adherence across six low and middle-income countries

Supplemental Table 5: Guideline adherence for specific recommendations, by sub-conditions

Recommendations	Bangladesh n %		Burkina Faso n %		Kenya n %		Malawi n %		Pakistan n %		Uganda n %		Overall n %	
	Full adherence	Not adherent	Full adherence	Not adherent	Full adherence	Not adherent	Full adherence	Not adherent	Full adherence	Not adherent	Full adherence	Not adherent	Full adherence	Not adherent
**PNEUMONIA[1]**														
Antibiotics for severe pneumonia	107<br>*76*	34<br>*24*	36<br>*46*	42<br>*54*	102<br>*68*	48<br>*32*	42<br>*89*	5<br>*11*	-	158<br>*100*	98<br>*81*	23<br>*19*	329<br>*52*	303<br>*48*
Antibiotics for non-severe pneumonia	101<br>*73*	37<br>*27*	8<br>*35*	15<br>*65*	5<br>*13*	33<br>*87*	12<br>*44*	15<br>*56*	17<br>*50*	17<br>*50*	16<br>*73*	6<br>*27*	129<br>*54*	112<br>*46*
Oxygen for severe pneumonia			78<br>*100 [3]*	-	71<br>*47 [4]*	79<br>*53 [4]*	6<br>*13 [4]*	41<br>*87 [4]*	158<br>*100 [3]*	-	121<br>*100 [3]*	-	434<br>*78*	120<br>*22*
Paracetamol for fever (Severe Pneumonia)			74<br>*95*	4<br>*5*			47<br>*100*	-	156<br>*99*	2<br>*1*	119<br>*98*	2<br>*2*	396<br>*98*	8<br>*2*
Paracetamol for fever (non-severe Pneumonia)							27<br>*100*	-			22<br>*100*	-	49<br>*100*	-
Bronchodilator for wheeze (severe pneumonia)			67<br>*86*	11<br>*14*	134<br>*89*	16<br>*11*	39<br>*83*	8<br>*17*	128<br>*81*	30<br>*19*	109<br>*90*	12<br>*10*	477<br>*86*	77<br>*14*
Bronchodilator for wheeze (non-severe pneumonia)					36<br>*95*	2<br>*5*	20<br>*74*	7<br>*26*			20<br>*91*	2<br>*9*	76<br>*87*	11<br>*13*
**DIARRHEA[2]**														
Rehydration for AWD with severe dehydration	9<br>*29*	22<br>*71*	14<br>*67*	7<br>*33*	2<br>*100*	-	3<br>*30*	7<br>*70*	8<br>*73*	3<br>*27*	9<br>*47*	10<br>*53*	45<br>*48*	49<br>*52*
Zinc for AWD with severe dehydration	22<br>*71*	9<br>*29*	13<br>*62*	8<br>*38*	1<br>*50*	1<br>*50*	4<br>*40*	6<br>*60*	3<br>*27*	8<br>*73*	3<br>*16*	16<br>*84*	46<br>*49*	47<br>*51*
Rehydration for AWD with some dehydration	49<br>*98*	1<br>*2*	3<br>*60*	2<br>*40*	17<br>*37*	29<br>*63*	3<br>*60*	2<br>*40*	1<br>*10*	9<br>*90*	8<br>*57*	6<br>*43*	81<br>*80*	20<br>*20*
Zinc for AWD with some dehydration			3<br>*60*	2<br>*40*	22<br>*48*	24<br>*52*	1<br>*20*	4<br>*80*	3<br>*30*	7<br>*70*	1<br>*7*	13<br>*93*	30<br>*28*	76<br>*72*

For peer review only

For peer review only - http://bmjopen.bmj.com/site/about/guidelines.xhtml

Rehydration for AWD with no dehydration	300	14	23	22	15	39	25	15	4	12	8	26	375	89
	96	*4*	*51*	*49*	*28*	*72*	*63*	*38*	*25*	*75*	*24*	*77*	*81*	*19*
No antibiotic in AWD	291	104	70	1	82	20	43	12	33	4	44	23	651	154
	74	*26*	*99*	*1*	*80*	*20*	*78*	*22*	*89*	*11*	*66*	*34*	*81*	*20*
Zinc for AWD with no dehydration			26	19	14	40	21	19	2	14	3	31	66	397
			58	*42*	*26*	*74*	*53*	*47*	*12*	*88*	*9*	*91*	*14*	*86*
Rehydration for persistent diarrhea			-	-	2	2	2	1	3	2	-	6	7	9
					50	*50*	*67*	*33*	*60*	*40*		*100*	*44*	*56*
Zinc for persistent diarrhea			-	-	2	2	1	2	1	4	-	6	4	12
					50	*50*	*33*	*67*	*20*	*80*		*100*	*25*	*75*
Vitamin for persistent diarrhea[5]			-	-			-	3			-	6	-	9
								100				*100*		*100*
Folate for persistent diarrhea			-	-			-	3					-	3
								100						*100*
Microminerals for persistent diarrhea			-	-			-	3					-	3
								100						*100*
Rehydration for severe persistent diarrhea			-	-			1	1	3	1	-	2	4	4
							50	*50*	*75*	*25*		*100*	*50*	*50*
Zinc for severe persistent diarrhea			-	-			-	2	-	4	-	2	-	8
								100		*100*		*100*		*100*
Vitamin for severe persistent diarrhea[5]			-	-			-	2			-	2	-	4
								100				*100*		*100*
Folate for severe persistent diarrhea			-	-			-	2					-	2
								100						*100*
Microminerals for severe persistent diarrhea			-	-			-	2					-	2
								100						*100*
Rehydration for dysentery			-	-	-	1	-	-	2	1	-	1	2	4
						100			*67*	*33*		*100*	*33*	*67*
Zinc for dysentery			-	-	-	1	-	-	1	2	-	1	1	5
						100			*33*	*67*		*100*	*17*	*83*
Antibiotics for dysentery	-	1	-	-	1	-	-	-	-	3	1	-	2	4
		100			*100*					*100*	*100*		*33*	*67*
**SM**														
Antibiotics in SM	236	33	41	140	161	57	62	21	2	116	206	21	710	386
	88	*12*	*23*	*77*	*74*	*26*	*75*	*25*	*2*	*98*	*91*	*9*	*65*	*35*
Fluid for dehydration and SM	71	5	16	1	10	10	35	8	8	8	1	1	141	33
	93	*7*	*94*	*6*	*50*	*50*	*81*	*19*	*50*	*50*	*50*	*50*	*81*	*19*

For peer review only

For peer review only - http://bmjopen.bmj.com/site/about/guidelines.xhtml

Blood transfusion in SM with severe anemia	-	-	21<br>*78*	6<br>*22*	3<br>*100*	-	-	-	1<br>*100*	-	-	-	25<br>*81*	6<br>*19*
Vitamin A in SM with measles			-	-	-	-			2<br>*25*	6<br>*75*	1<br>*14*	6<br>*86*	3<br>*20*	12<br>*80*
Micronutrients in SM	143<br>*53*	126<br>*47*	68<br>*38*	113<br>*62*	114<br>*52*	104<br>*48*			22<br>*19*	96<br>*81*	72<br>*32*	155<br>*68*	467<br>*43*	629<br>*57*
Fortified food in SM	262<br>*97*	7<br>*3*	153<br>*85*	28<br>*16*	184<br>*84*	34<br>*16*	62<br>*74*	21<br>*25*	58<br>*49*	60<br>*51*	147<br>*65*	80<br>*35*	866<br>*79*	230<br>*21*
Zinc for skin lesion in SM									8<br>*16*	42<br>*84*	-	109<br>*100*	8<br>*5*	151<br>*95*

[1] For pneumonia, comorbid SM was excluded as per Kenyan pneumonia treatment guidelines
[2] For diarrhea, SM cases are excluded from all countries
[3] Oxygen is recommended for severe pneumonia cases with SpO2<90% only
[4] Oxygen is recommended for all severe pneumonia cases
[5] Vitamins include vitamin A, folate, both, and/or multivitamin
Notes: (1) The denominator for all percentages is the total number of children with a specific condition, (2) AWD= Acute Watery Diarrhea, (3) A "-" indicates that there were no cases in the sample with the diagnosis (4) a blank cell indicates there are no relevant recommendation for a condition in the country

For peer review only

Supplemental Table 6: Adherence to pneumonia and associated sub-conditions, excluding children with SM (sensitivity analysis)

Pneumonia sub-conditions and specific recommendations	Total N	Full adherence n (%)	Partial adherence n (%)	No adherence n (%)
**Pneumonia**	659	187 (28)	385 (58)	87 (13)
**Non-severe Pneumonia**	169	54 (32)	56 (33)	59 (34)
Antibiotics	169	57 (34)		112 (66)
Paracetamol if fever	33	33 (100)		
Bronchodilator if wheeze	71	62 (87)		9 (13)
**Severe Pneumonia**	490	133 (27)	329 (67)	28 (6)
Antibiotics	490	242 (49)		248 (51)
Oxygen	430[1]	320 (74)		110 (26)
Paracetamol if fever	280	274 (98)		6 (2)
Bronchodilator if wheeze	430	372 (87)		58 (13)

[1] Oxygen is only given when low saturation in sever pneumonia in some countries

For peer review only

Prevalence and correlates of pediatric guideline non-adherence across six low and middle-income countries

Supplemental Table 7: Overall guideline adherence for conditions and sub-conditions without comorbidities

Conditions and sub-conditions	Overall n %		
	Full	Partial	None
**PNEUMONIA**	51 (27)	120 (64)	17 (9)
Non-severe	8 (21)	18 (47)	12 (32)
Severe	43 (29)	102 (68)	5 (3)
**DIARRHEA**	111 (37)	148 (49)	45 (15)
AWD w/ severe dehydration	1 (5)	14 (64)	7 (32)
AWD w/ some dehydration	4 (11)	28 (74)	6 (16)
AWD w/ no dehydration	106 (47)	105 (46)	16 (7)
Persistent	-	1 (33)	2 (67)
Severe persistent	-	-	1 (100)
Dysentery	-	4 (67)	2 (33)
**SM**	22 (22)	78 (78)	
W/ hypoglycemia	2 (67)	1 (33)	-
W/ hypothermia	-	3 (100)	-
SM with dehydration	2 (33)		4 (67)
SM with severe anemia	-	-	-
SM with active measles	-	-	-
W/ micronutrient deficiency	54 (54)	-	46 (46)
W/ infection	75 (75)	9 (9)	16 (16)
SM requiring nutritional stabilization	76 (76)	-	24 (24)
W/ skin lesion	1 (3)	-	34 (97)

Notes: (1) The denominator for all percentages is the total number of children with a specific condition without comorbidities, (2) AWD= Acute Watery Diarrhea, (3) A "-" indicates that there were no cases in the sample with the diagnosis and without comorbidities

For peer review only

STROBE Statement—Checklist of items that should be included in reports of ***cross-sectional studies***

	Item No	Recommendation	Page No
**Title and abstract**	1	(*a*) Indicate the study's design with a commonly used term in the title or the abstract	1
		(*b*) Provide in the abstract an informative and balanced summary of what was done and what was found	6
**Introduction**			
Background/rationale	2	Explain the scientific background and rationale for the investigation being reported	9-10
Objectives	3	State specific objectives, including any prespecified hypotheses	9
**Methods**			
Study design	4	Present key elements of study design early in the paper	10
Setting	5	Describe the setting, locations, and relevant dates, including periods of recruitment, exposure, follow-up, and data collection	10
Participants	6	(*a*) Give the eligibility criteria, and the sources and methods of selection of participants	10
Variables	7	Clearly define all outcomes, exposures, predictors, potential confounders, and effect modifiers. Give diagnostic criteria, if applicable	11-12
Data sources/ measurement	8*	For each variable of interest, give sources of data and details of methods of assessment (measurement). Describe comparability of assessment methods if there is more than one group	11-12
Bias	9	Describe any efforts to address potential sources of bias	13
Study size	10	Explain how the study size was arrived at	15
Quantitative variables	11	Explain how quantitative variables were handled in the analyses. If applicable, describe which groupings were chosen and why	13
Statistical methods	12	(*a*) Describe all statistical methods, including those used to control for confounding	13
		(*b*) Describe any methods used to examine subgroups and interactions	14
		(*c*) Explain how missing data were addressed	13
		(*d*) If applicable, describe analytical methods taking account of sampling strategy	N/A
		(*e*) Describe any sensitivity analyses	14
**Results**			
Participants	13*	(a) Report numbers of individuals at each stage of study—eg numbers potentially eligible, examined for eligibility, confirmed eligible, included in the study, completing follow-up, and analysed	15
		(b) Give reasons for non-participation at each stage	N/A
		(c) Consider use of a flow diagram	N/A
Descriptive data	14*	(a) Give characteristics of study participants (eg demographic, clinical, social) and information on exposures and potential confounders	15
		(b) Indicate number of participants with missing data for each variable of interest	13
Outcome data	15*	Report numbers of outcome events or summary measures	15-20

For peer review only

Main results	16	(*a*) Give unadjusted estimates and, if applicable, confounder-adjusted estimates and their precision (eg, 95% confidence interval). Make clear which confounders were adjusted for and why they were included	22-23
		(*b*) Report category boundaries when continuous variables were categorized	N/A
		(*c*) If relevant, consider translating estimates of relative risk into absolute risk for a meaningful time period	N/A
Other analyses	17	Report other analyses done—eg analyses of subgroups and interactions, and sensitivity analyses	19
**Discussion**			
Key results	18	Summarise key results with reference to study objectives	24
Limitations	19	Discuss limitations of the study, taking into account sources of potential bias or imprecision. Discuss both direction and magnitude of any potential bias	27
Interpretation	20	Give a cautious overall interpretation of results considering objectives, limitations, multiplicity of analyses, results from similar studies, and other relevant evidence	23-27
Generalisability	21	Discuss the generalisability (external validity) of the study results	26
**Other information**			
Funding	22	Give the source of funding and the role of the funders for the present study and, if applicable, for the original study on which the present article is based	29

For peer review only
